# Synthesis and Evaluation of DNA Cross‐linkers by Click Chemistry‐Mediated Heterodimerization of Nor‐Tomaymycins

**DOI:** 10.1002/chem.202501797

**Published:** 2025-08-22

**Authors:** Julia Friederich, Katharina Rox, Hazel L. S. Fuchs, Md. Mahbub Hasan, Patrick Raunft, David E. Thurston, Keith R. Fox, Khondaker Miraz Rahman, Mark Brönstrup

**Affiliations:** ^1^ Department of Chemical Biology Helmholtz Centre for Infection Research Inhoffenstraße 7 38124 Braunschweig Germany; ^2^ German Center for Infection Research (DZIF) Site Hannover‐Braunschweig 38124 Braunschweig Germany; ^3^ Department of Genetic Engineering and Biotechnology Faculty of Biological Sciences University of Chittagong, Chittagong Chattogram 4331 Bangladesh; ^4^ Institute of Pharmaceutical Science School of Cancer & Pharmaceutical Sciences King's College London Franklin‐Wilkins Building 150 Stamford Street London UK; ^5^ School of Biological Sciences, Life Sciences Building 85 University of Southampton Southampton SO17 1BJ UK; ^6^ Institute of Organic Chemistry Leibniz University Hannover 30167 Hannover Germany

**Keywords:** click chemistry, DNA binder, drug discovery, natural products

## Abstract

The covalent cross‐linking of DNA duplex strands by small molecule drugs is a validated mechanism in anticancer therapy. The pyrrolo[2,1‐*c*][1,4]benzodiazepines (PBDs) have been established as potent DNA binders that can achieve inter‐ and intrastrand DNA cross‐links when dimerized, and have been used as cytotoxic payloads in multiple antibody‐drug conjugates (ADCs). In this study, we explore the potential of click chemistry to obtain PBD heterodimers. The heterodimers **D1**–**D4** were prepared by copper catalyzed or strain‐promoted azide‐alkyne cycloadditions (SPAACs) from the corresponding monomers **M1** – **M4** and **MbA**. The interactions of monomers and dimers with DNA were evaluated by DNA thermal denaturation analysis, a newly developed mass spectrometry‐based method for the detection of single stand and double strand modifications, and by DNase I footprinting. All methods demonstrated the DNA binding and cross‐linking capabilities of **D1**. Testing for cytotoxicity in three cell lines revealed that the monomers **M1** and **M4** and the dimers **D1** and **D4** were the most potent. The click chemistry approach opens an easy access to larger libraries of PBD‐based DNA binders, including the option for dimerization in live cells.

## Graphical abstract

Click chemistry promises a simple method to produce pyrrolo[2,1‐*c*][1,4]benzodiazepine (PBD)‐based DNA binders. DNA binding and covalent crosslinking capabilities have been validated for ‘clicked’ PBD heterodimers using thermal denaturation analysis, mass spectrometry and DNase I footprinting. One dimer induced strong DNA cross‐linking and cellular cytotoxicity, indicating the future potential for dimerization in living cells.

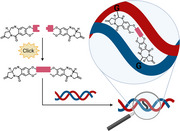

## 1 Introduction

**Figure 1:**
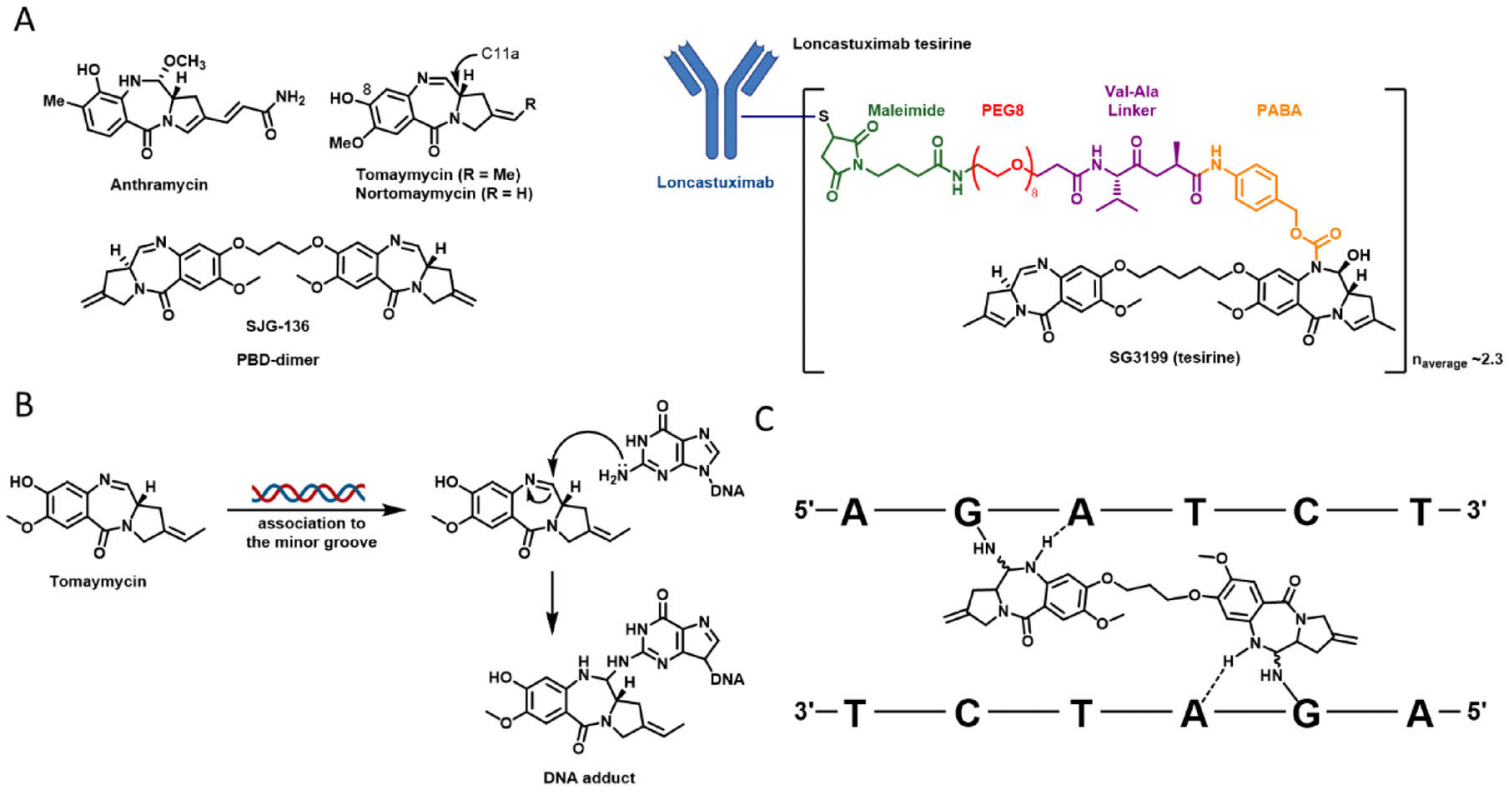
Structures and mechanism of action of selected pyrrolo[2,1‐c][1,4]benzodiazepines. A) Structures of the natural products anthramycin and tomaymycin, the synthetic analog nor‐tomaymycin, the dimer SJG‐136, and the antibody‐drug conjugate loncastuximab tesirine, which contains the PBD dimer SG3199 as its payload. B) Covalent DNA adduct formation by tomaymycin with the guanine base of DNA. C) DNA cross‐linking by SJG‐136.

DNA minor groove binders constitute an important class of chemotherapeutics with potent anticancer and/or antibiotic properties. Among them, the pyrrolo[2,1‐*c*][1,4]benzodiazepines (PBDs) have been explored in depth, due to their ability to bind covalently to DNA in a sequence‐specific manner.^1^ ^]^ The class of PBDs originates from natural products produced by *Streptomyces* sp., such as anthramycin, or tomaymycin (Figure 1A).^[^ ^2^, ^3^ ^]^ Their tricyclic structures are composed of a substituted aromatic A‐ring, a diazepine B‐ring and a pyrrolidine C‐ring. Two features of their structure are essential for bioactivity: The *S*‐configured chiral center at the C11a‐position adopts a right‐handed helical shape that allows the molecule to fit into the DNA minor groove,^[^ ^4^ ^]^ and the electrophilic imine which is necessary for covalent binding to the exocyclic N2‐amino groups of guanine bases (Figure 1B).^[^ ^5^, ^6^, ^7^ ^]^

PBDs have been extensively studied as anticancer agents,^[^ ^8^, ^9^ ^]^ and more recently as antibacterials.^[^ ^10^, ^11^ ^]^ The bioactivity of PBDs is potentiated by several orders of magnitude through dimerization, because two alkylating imine functionalities confer interstrand or intrastrand DNA cross‐linking properties in addition to mono‐alkylation.^[^ ^12^ ^]^ An optimized PBD dimer was obtained by connecting nor‐tomaymycin, a tomaymycin derivative lacking the exocyclic methyl residue, at the 8‐ and 8′‐positions with a 1,3‐propanedioxy ether linker. The resulting drug, SJG‐136, preferentially forms interstrand cross‐links with the sequences R‐GATC‐Y (Figure 1C);^[^ ^12^, ^13^, ^14^, ^15^, ^16^ ^]^ SJG‐136 reached Phase II clinical trials in ovarian cancer and leukaemia.^[^ ^1^ ^]^ The greatest clinical success of PBD dimers has been achieved by incorporating them as payloads in antibody‐drug conjugates (ADCs), with the first marketed drug named loncastuximab tesirine (Figure 1A).^[^ ^17^, ^18^ ^]^

While previous PBD dimers were synthesized by classical, sequential linker chemistry, we decided to explore whether the joining of PBD monomers to form functional dimers could be achieved via click chemistry. It was hypothesized that applying 1,3‐dipolar cycloaddition between an alkyne and an azide, archetypal components of a click reaction, should enable the assembly of a large set of PBD dimers in a modular fashion. It was further hypothesized that, in the future, it may be possible to observe the dimerization of click‐enabled PBD monomers taking place in living cells through noncatalyzed click reactions based on strained alkynes. We demonstrate the feasibility of this concept, using click chemistry to produce four PBD dimers, followed by evaluation of their DNA binding and cellular cytotoxicity.

## 2 Results

### 2.1 Compound Synthesis

**Scheme 1:**
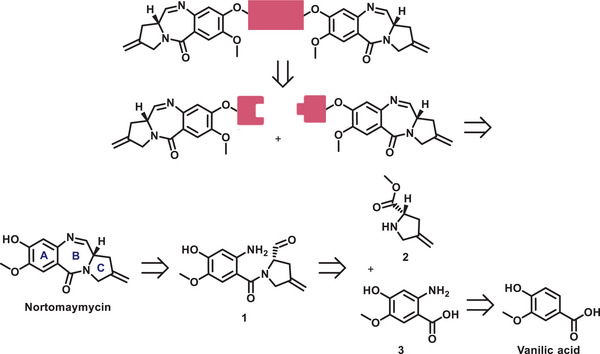
Retrosynthesis of PBD dimers via nor‐tomaymycin as a monomer.

We designed a set of different PBD dimers that could be obtained through monomer coupling via a click reaction as the last synthesis step (Scheme 1). For the monomers, nor‐tomaymycin was chosen as the core structure to retain the favorable properties of the optimized drug SJG‐136. The core structure was then equipped with clickable handles to allow for the final coupling step.

The retrosynthesis of the nor‐tomaymycin core structure was planned based on previously published routes for PBD synthesis.^[^ ^3^, ^19^ ^]^ It involved a final cyclization of the B ring via an intramolecular condensation between an aromatic amine and an aldehyde to form the corresponding imine (Scheme 1). The A‐ and C‐ring fragments originated from vanillic acid and methyl (*S*)‐4‐methylenepyrrolidine‐2‐carboxylate, respectively, as commercially available building blocks.

**Scheme 2:**
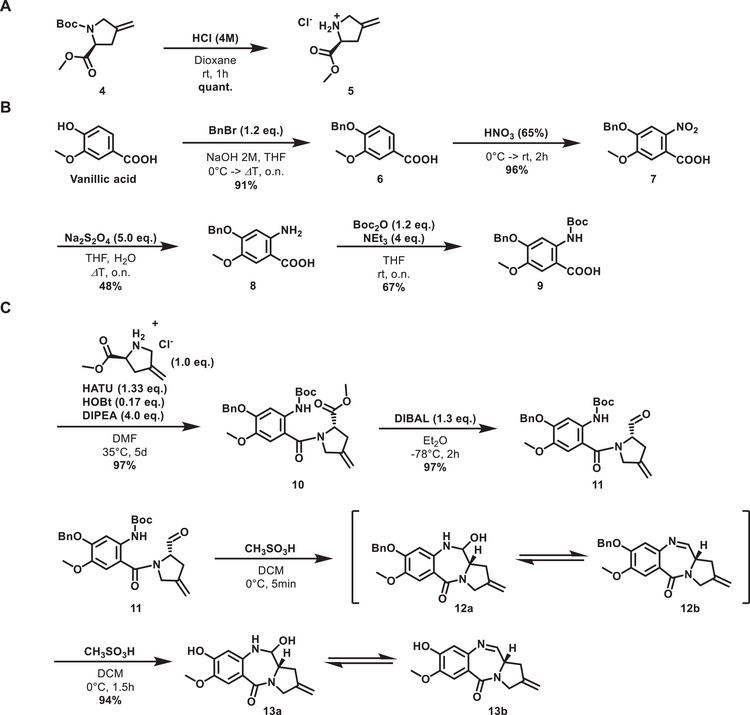
Synthesis of nor‐tomaymycin core. A) Synthesis of C ring fragment 5; B) Synthesis of A‐ring fragment 9; C) Synthesis of B‐ring 13 via connection of ring A and ring C.

In the published route, the nitrobenzoic acid **7** was coupled with the amine **5** to form the amide of ring B. ^[^ ^19^ ^]^ However, all our attempts to reduce the ester to an aldehyde and the nitro group to an aniline to enable the B‐ring condensation failed. Therefore, **7** was first reduced to the anthranilic acid **8** and then Boc‐protected. The reduction with Zn/HCl was completed within 10 minutes and led to high yields of > 90%, but it was detrimental for the Boc protection in the next step. Pícha et al and Wiejak et al reported that bivalent ions can be used as protecting groups for the amine of α‐amino acids through a bidental coordination by two α‐amino acids.^[^ ^20^, ^21^ ^]^ The complete suppression of the Boc protection suggests a similar role for the Zn^2+^ ions present after the Zn/HCl reduction. This was avoided by using sodium dithionite as a reducing agent (48% yield) and enabled the subsequent Boc protection in 67% yield. The resulting intermediate **9** was amidated with **5** using HATU and reduced to the aldehyde by DIBAL with yields of 97% and 97%, respectively. LC‐MS analysis after treatment of **11** with methanesulfonic acid in DCM at 0 °C showed that, within 5 minutes, deprotection of the Boc group and subsequent ring closure to the hemiaminal occurred, which was in equilibrium with the imine due to water elimination. If the reaction was continued with stirring for 1.5 hours, the benzyl protective group cleaved to release nor‐tomaymycin **13b**. Overall, the nor‐tomaymycin core was obtained in 25% yield over 7 steps (Scheme 2).

**Scheme 3:**
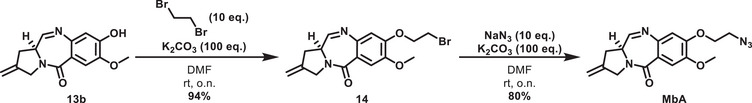
Synthesis of monomer **MbA** containing an azide handle at the phenolic position of the nor‐tomaymycin core.

In the next step, different reactive handles for the final dimerization reaction were introduced at the phenolic position of nor‐tomaymycin. Specifically, monomers with reactive linkers for the azide‐alkyne cycloaddition and the strain‐promoted azide‐alkyne cycloaddition (SPAAC) were prepared. The corresponding azide was obtained in two steps. First, the nor‐tomaymycin core **13b** was treated with 1,2‐dibromoethane under basic conditions to obtain the bromide **14** in 94% yield (Scheme 3). The tenfold excess of 1,2‐dibromoethane was crucial to prevent the formation of a symmetric nor‐tomaymycin‐dimer through double nucleophilic substitution. Subsequently, the bromide **14** was subjected to a second substitution with sodium azide, yielding the desired azide monomer **MbA** (monomer‐based azide) in 80% yield.

**Scheme 4:**
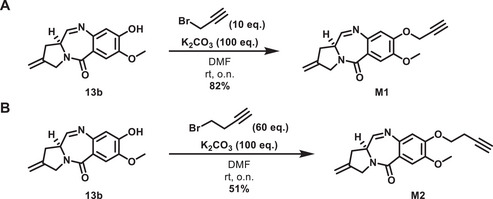
Synthesis of monomers **M1** and **M2** with alkyne handles separated by one‐ or two‐carbon spacers from the nor‐tomaymycin core.

To act as the second reaction partner in the CuAAC reaction, two alkynes of different lengths were synthesized. The first (**M1**) has a methylene unit between the phenol and alkyne functionalities, while the second (**M2**) bears an ethylene unit. Both compounds were obtained through substitution reactions of the respective alkyl bromides under basic conditions (Scheme 4). The resulting yield with homopropargyl bromide was lower than that with propargyl bromide (51% versus 82%) due to a pronounced elimination reaction in the former case that was compensated for by a large excess (60 eq.) of the electrophile.

**Scheme 5:**
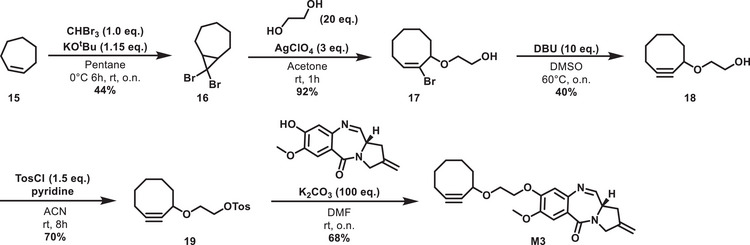
Synthesis of monomer **M3** with a cyclooctyne handle.

To allow also for a SPAAC, two different cyclooctyne linkers were added to the core to provide **M3** and **M4**, respectively. **M3** was obtained in five steps (Scheme 5), starting with the addition of bromoform to the double bond of cycloheptene under basic conditions to yield the bicyclic intermediate **16**. Subsequently, silver perchlorate mediated an electrocyclic ring expansion of **16** to give the cyclooctene **17**, which incorporated the ethylene glycol‐based handle in the same step (yield: 92%). Next, a DBU‐promoted elimination was performed to form the strained cyclooctyne **18**.^[^ ^22^ ^]^ After tosylating the primary alcohol of **18** in 70% yield under basic conditions, nor‐tomaymycin was attached by nucleophilic substitution in 68% yield using potassium carbonate as a base and DMF as a solvent.

**Scheme 6:**
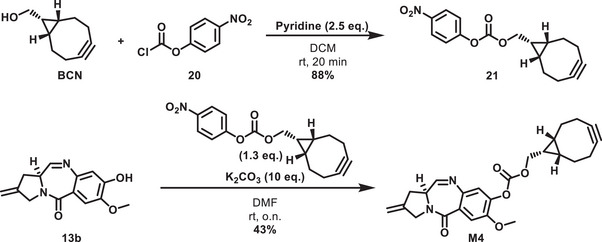
Synthesis of monomer M4 containing a bicyclo[6.1.0]non‐4‐yne (BCN) handle.

Despite the simplicity of this strained cyclooctyne, it potentially suffers from a disadvantage. As the enantiotopic bridgehead carbons of **17** are not differentiated by ethylene glycol, the C1 position of **M3** was obtained as a mixture of enantiomers. After the cycloaddition of azides to **M3**, two regioisomers and two diastereomers (i.e., a total of four products) can be formed. To reduce the number of stereoisomers, the symmetric bicyclo[6.1.0]non‐4‐yne (BCN) was identified as an alternative strained alkyne. However, cycloaddition with substituted azides still resulted in a mixture of two enantiomers. While the connection of nor‐tomaymycin with BCN via simple alkyl linkers failed, a mixed carbonate could be prepared in two steps to give **M4** (Scheme 6).

**Scheme 7:**
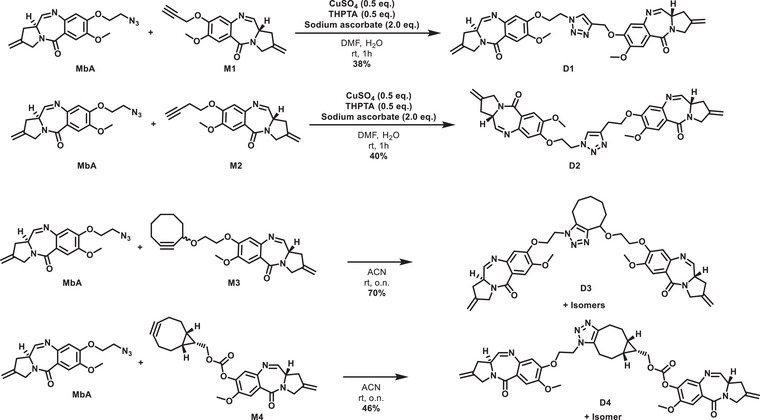
Synthesis of the heterodimers **D1**–**D4**.

The azide **MbA** and the alkynes **M1**‐**M4** constituted the set of monomers that were coupled to the corresponding heterodimers **D1**‐**D4**. The triazoles **D1** and **D2** were prepared by copper(I)‐catalyzed azide‐alkyne cycloadditions (CuAAC) of **MbA** and **M1** and **M2**, respectively (Scheme 7). Dimers **D3** and **D4** on the other hand were synthetized via a SPAAC reaction. **D4** features a carbonate functionality that was fully stable in methanol or under aqueous conditions. In summary, four novel nor‐tomaymycin dimers with asymmetric linkers were obtained through biorthogonal reactions.

### 2.2 Functional Characterization of Nor‐tomaymycin Derivatives

The monomers **MbA** and **M1**‐**M4** along with the corresponding dimers **D1**‐**D4** were studied by three different methods to establish whether they could bind to DNA or, in the case of **D1**‐**D4**, form DNA cross‐links.

### 2.3 DNA Thermal Denaturation Studies

**Figure 2:**
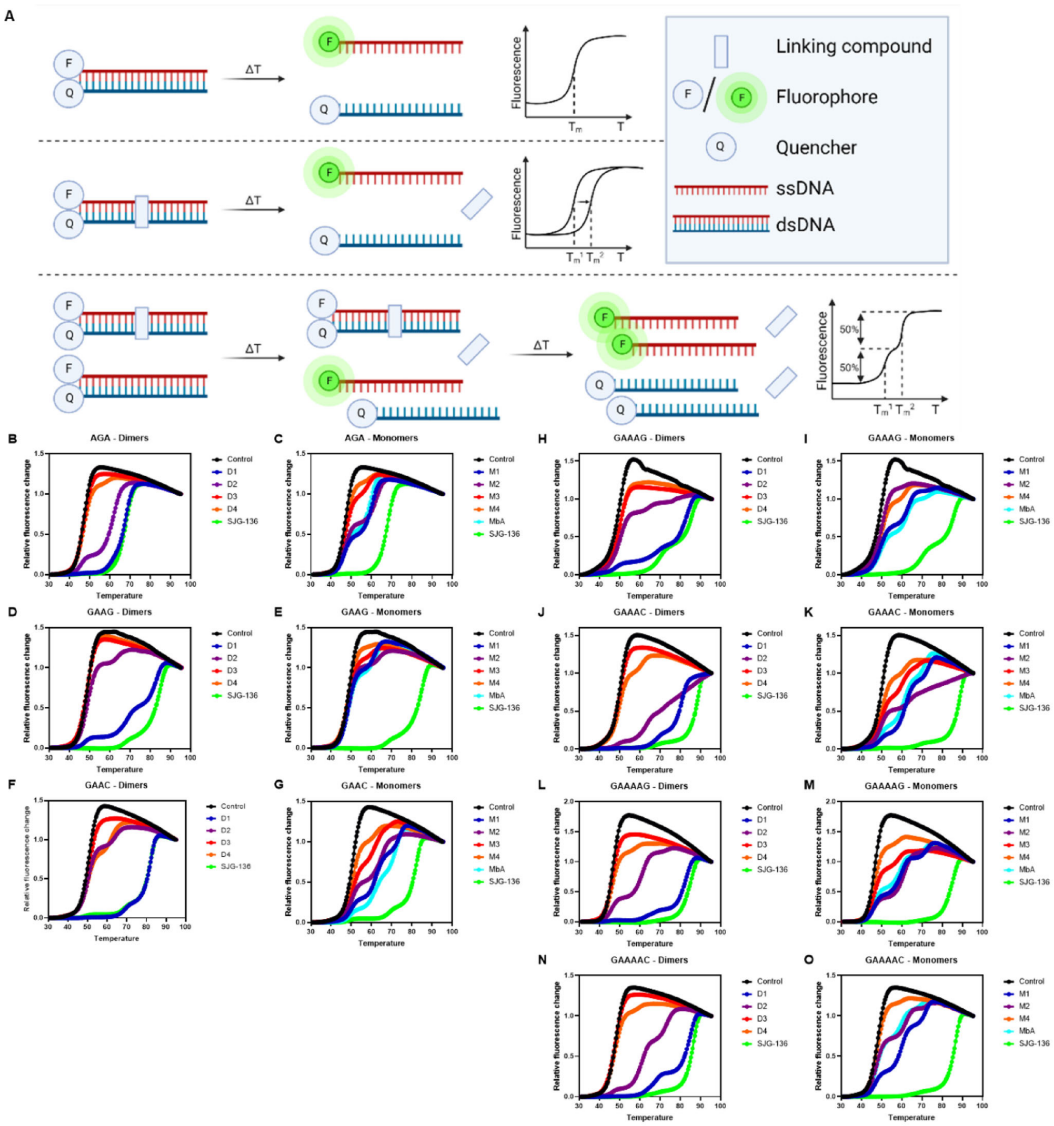
Fluorescence melting curves in the presence of the PBDs. A) Schematic overview of the fluorescence DNA melting assay, created with BioRender.com. B‐O) Melting profiles of different sequences in the presence or absence of 10 µM of the PBDs. B and C), sequence 1 (AGA); D and E), sequence 2 (GAAG); F and G) sequence 3 (GAAC); H and I), sequence 4 (GAAAG); J and K), sequence 5 (GAAAC); L and M), sequence 6 (GAAAAG); N and O), sequence 7 (GAAAAC). Heterodimers **D1**, **D2**, **D3**, and **D4** were added in panels B, D, F, H, J, L, and N) and monomers **M1**, **M2**, **M3**, **M4**, and **MbA** in panels C, E, G, I, K, M, and O). The controls (black) represent melting of the DNA duplex without any added compound. SJG‐136 (green) served as a positive control. The sequences of the central binding motifs are indicated above each panel.

The first method involved DNA thermal denaturation measurements on the effects of the nor‐tomaymycins on the melting temperatures of a series of oligonucleotides. DNA duplexes were designed that contained an anthramycin‐binding motif embedded within an A_n_·T_n_ tract, with a 5′‐fluorescein‐labeled A‐strand and a 3′‐dabcyl‐labeled T‐strand. When these combine to form a duplex, the emission of the fluorescein is quenched by the dabcyl moiety due to their close proximity (Figure 2A). The two strands separate when the temperature is increased, leading to an increase in fluorescence.^[^ ^23^ ^]^ Introduction of DNA‐binding and/or cross‐linking compounds should produce a shift to higher melting temperatures (T_m_^1^ → T_m_^2^) with one or more transitions observed if different states (e.g., covalent and noncovalent adducts, mono – or bis‐adducts) are present simultaneously.

A set of seven sequences with different binding motifs was chosen, representing different distances between the two Gs, as well as allowing the potential formation of inter‐ or intra‐molecular cross‐links (Table 1). The effect of **MbA** and **M1**‐**M4**, the corresponding dimers **D1**‐**D4** and SJG‐136, on the melting curve of these oligonucleotides was investigated, and the results are presented in Figure 2 and Table S1. The untreated controls showed one sigmoidal transition with its inflection point, indicating the sequence's melting temperature (Tm). In all sequences, SJG‐136 induced the largest shifts in Tm of all the new molecules examined.

**Table 1:** Sequences of the fluorescently‐labelled oligonucleotides for the DNA thermal denaturation studies. F = Fluorophore (fluorescein), Q = Quencher (dabcyl).

Name	Binding Motif	Sequence
Sequence 1	AGA	F‐AAAAAAA **G** AAAAAAAAA 3′ Q‐TTTTTTT **C** TTTTTTTTT 5′
Sequence 2	GAAG	F‐AAAAA **GAAG** AAAAAAAA‐3′ Q‐TTTTT **CTTC** TTTTTTTT‐5′
Sequence 3	GAAC	F‐AAAAA **GAAC** AAAAAAAA‐3′ Q‐TTTTT **CTTG** TTTTTTTT‐5′
Sequence 4	GAAAG	F‐AAAAA **GAAAG** AAAAAAA‐3′ Q‐TTTTT **CTTTC** TTTTTTT‐5′
Sequence 5	GAAAC	F‐AAAAA **GAAAC** AAAAAAA‐3′ Q‐TTTTT **CTTTG** TTTTTTT‐5′
Sequence 6	GAAAAG	F‐AAAAA **GAAAAG** AAAAAA‐3′ Q‐TTTTT **CTTTTC** TTTTTT‐5′
Sequence 7	GAAAAC	F‐AAAAA **GAAAAC** AAAAAA‐3′ Q‐TTTTT **CTTTTG** TTTTTT‐5′

Sequence 1, containing the binding motif AGA, provided only one guanine for binding. Therefore, it had no possibility for cross‐linking or multiple drug binding, and thus can only form mono‐adducts with one PBD bound per duplex. PBD dimers are generally capable of engaging with DNA to form a mono‐adduct. SJG‐136, **D1** and **D2**, led to significant shifts in T_m_ to 67.4 °C, 66.7 °C, and 62.0 °C, respectively, compared to 47.0 °C for the free duplex (Figure 2B,C). Interestingly, the melting profile of sequence 1 incubated with **D2** showed two transitions. The first represents melting of the free oligonucleotide, with the same *T*_m_ as the control. The second transition reflects the melting of the duplex stabilized by **D2** binding. The relative amounts of these two transitions indicates the ratio of the free duplex to the ligand‐bound complex. It can be seen that **D1** produced greater stabilization of the double strand than **D2**. In contrast, **D3** and **D4** produced little or no change in *T*_m_, indicating that these ligands do not bind to sequences that contain only one guanine. This may suggest that steric clashes with the sterically demanding cyclooctyne ring appear to prevent binding to the DNA minor groove. The same experiment was conducted with the monomers to investigate their binding abilities. **M3** and **M4** had very little effect on the melting profiles, while **MbA**, **M1**, and **M2** generated profiles in which some of the transition occurred at a higher temperature, though this was much lower than the ΔTm produced by SJG‐136, **D1** or **D2**.

Next, the binding properties of the compounds were tested with oligonucleotides that contained two guanine residues (Figure 2D–O). In these sequences the two guanines were separated by different numbers of As, and were designed to allow the formation of intra‐ or interstrand cross‐links. In sequence 2, the two guanines are separated by two adenines and are positioned on the same strand, potentially enabling intrastrand cross‐links. **D1** caused a significant change in the melting profile with three transitions of increasing relative amounts (Figure 2D): The first corresponds to the free duplex, while the second has a similar melting transition as seen with sequence 1, which is only capable of forming mono‐adducts. The third transition with the greatest stabilization either indicates the formation of a cross‐linked species or of two simultaneous monoadducts. **D2** also produced some smaller shifts in the melting profile, while the effects of **D4** and **D3** were minimal.

The profiles with the monomers also revealed a second melting transition at 61.1 °C, 61.3 °C, 63.5 °C, and 59.9 °C for **MbA** and **M1**‐**M3**, respectively (Figure 2E and Table S1). This is in the same range (i.e., ∼60 °C) as observed for the mono‐adducts with sequence 1. However, the relative amounts of the two transitions reveals that only about 10–20% of the DNA molecules were bound by the monomers. In contrast, approximately 40–60% of the oligonucleotides were bound by the monomers with sequence 1. For **M3** and **M4** the fraction of unbound duplex was even higher.

Sequence 3 contains the sequence GAAC, in which the two guanines are also separated by two bases, but the second guanine is positioned on the opposite strand, allowing for interstrand cross‐links. Introducing this modification changed the melting profiles compared to sequence 2 (Figure 2F). While **D3** did not show any effect, all other compounds affected the melting profile. **D4**, which had no effect on sequence 2 produced about 30% of the melt with a *T*_m_ of 62.1 °C, which is typical of mono adduct formation. Similarly, **D2** showed an increased amount of bound duplex. **D1** produced the greatest change in melting temperature, with a similar effect to that generated with SJG‐136, in which most of the profile corresponds to the highest melting temperature, which is consistent with formation of a cross‐linked species. The change in the ratio of free to bound or cross‐linked DNA implies that **D1** preferentially formed interstrand rather than intrastrand cross‐links.

Figure 2G shows the effect of the monomers on this sequence, in which it can be seen that **M1** produced a triphasic melting profile, though the highest transition is much lower than SJG‐136 or **D1**. This result suggests that two monomer molecules bind independently to each of the two guanines. This is consistent with the observation that the effect is greater with increasing distance between the two guanines, which would facilitate binding of a second PBD monomer.

To further investigate the effect of the distance between the two guanine residues, sequences 4 to 7 were examined, which contain three or four adenines between the two Gs. Positioning of the guanines on the same or opposite strands would allow the formation of intrastrand (sequences 4 and 6) or interstrand (sequences 5 and 7) cross‐links. The binding patterns were similar in all four cases. **D1** was again the best‐performing dimer, followed by **D2**. **D3** and **D4** showed only minor or no effects on the respective melting profiles.

The monomers **M1**, **M2**, and **MbA**, again produced triphasic melting profiles, reflecting the simultaneous binding of the ligands to each of the guanines. In contrast, **M3** and **M4** only produced biphasic melting curves, probably due to the steric clashes with a second monomer.

The cyclooctene **D3** was the poorest binder out of the compounds tested, closely followed by the other cyclooctene **D4**, which bound to only 10% of the duplexes. A larger increase in melting temperature, as well as a greater relative proportion of bound duplex, was achieved by the triazole **D2**. The triazole **D1**, which has a slightly shorter linker than **D2**, gave the best results, both in terms of Δ*T*_m_ and the relative proportion of the duplex that is stabilized. A similar trend was seen for the monomers. The two monomers **M3** and **M4** carrying a cyclooctyne moiety showed poorer binding than the alkynes **M1**, **M2**, and the azide **MbA**, most likely due to the steric bulk of the cyclooctyne rings. The simultaneous incubation with **MbA** + **M1** or **MbA** + **M2** gave no additive effect beyond that of the monomers alone (Table S2).

### 2.4 HPLC‐MS/MS Studies

**Figure 3:**
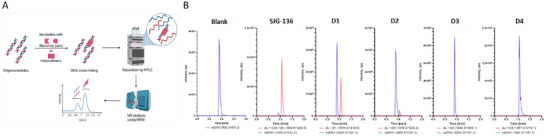
Mass spectrometric detection of DNA interstrand cross‐links by SJG‐136 and the heterodimeric PBDs **D1** and **D2**. A) Schematic overview of the experimental workflow. The chromatographic conditions lead to the separation of dsDNA to ssDNA, unless they are covalently cross‐linked. Created with BioRender.com. B) Chromatographic traces for free DNA (single‐stranded after dissociation) as well as double‐stranded DNA cross‐lined by SJG‐136 and **D1**–**D4** bound to the sequence TATAGATCTATA. Signals are displayed as total ion chromatograms (TIC) of all multiple reaction monitoring (MRM) pairs.

In order to confirm the covalent binding properties of the new PBD molecules to DNA, we developed an assay using high‐pressure liquid chromatography coupled to tandem mass spectrometry (HPLC‐MS/MS). A key advantage of this method is that mass spectrometry can directly detect covalent PBD‐DNA cross‐links rather than inferring them from biophysical methods such as DNA thermal denaturation. Another advantage of employing mass spectrometry is its enhanced sensitivity compared to gel‐based readouts. The assay, whose development was based on previously reported simpler variants,^[^ ^13^, ^24^ ^]^ started by annealing two single‐stranded complementary 12‐mer oligonucleotides containing a TATAGATCTATA sequence. The double‐stranded DNA (dsDNA) obtained was incubated with the monomeric and dimeric PBD molecules for 24 hours and then analyzed by HPLC‐MS/MS (Figure 3A). The denaturing conditions of the HPLC separation due to the high organic solvent content of the mobile phase caused noninterstrand‐cross‐linked dsDNA to dissociate into single‐stranded DNA (ssDNA) species. It is also assumed to disrupt any noncovalent DNA‐ligand complexes. In contrast, if dsDNA oligonucleotides were interstrand‐cross‐linked by PBDs, the two strands remained covalently bound and behaved as a dsDNA‐PBD adduct. In this case, ssDNA and cross‐linked DNA could be separated by HPLC due to their different physiochemical properties. The separated components were then ionized by electrospray in the negative mode and mass‐analyzed in a triple quadrupole mass spectrometer. The latter was operated in a targeted mode using multiple reaction monitoring (MRM) in order to achieve optimal signal‐to‐noise ratios and low limits of detection.

Incubating the ds oligonucleotides with SJG‐136 in a molar ratio of 1:4 as a positive control led to two peaks in the HPLC with retention times of 0.91 minutes and 1.05 minutes, corresponding to ssDNA and the dsDNA‐SJG‐136 adduct, respectively (Figures 3B and S1, Tables S3, S6). The identity of the cross‐linked dsDNA‐SJG‐136 adduct was confirmed by collision‐induced dissociation, which caused its fragmentation into ssDNA in the gas phase (e.g., precursor ion: 1119.0 Da (= [dsDNA + SJG‐136]7^−^) → product ion: 909.6 (= [ssDNA]4^−^). Ions from the interstrand cross‐linked form were far more abundant than ions from ssDNA. This result was consistent with the results from the DNA thermal denaturation studies described above, and underscores the highly effective cross‐linking ability of SJG‐136. Next, the LC‐MS/MS assay was used to investigate the DNA binding of the PBD dimers **D1**‐**D4** (Figure 3B). For **D1**, a pronounced formation of the cross‐linked dimer was detected at 1.1 minutes, although the peak from unbound ssDNA at 0.9 minute was higher (ratio ca 0.46:1). For **D2**, a peak for the cross‐linked dimer was also observed, but it was small compared to the ssDNA peak (ratio ca. 0.07:1). In contrast, the two heterodimers based on SPAAC (*i.e*., **D3** and **D4**, did not produce any DNA cross‐linking. Overall, the assay was useful in providing direct evidence that PBD dimers **D1** and **D2** can form interstrand cross‐links with DNA.

### 2.5 DNase I Footprinting

A third method, DNase I footprinting, was used to evaluate the binding sites of the compounds.^23^, ^25^ While the previous two methods used relatively short oligonucleotide sequences, a longer DNA fragment, which contains all 136 tetranucleotide sequences, was used for the DNase I footprinting experiments.^[^ ^23^, ^25^ ^]^ The basis of the assay is that binding of a PBD molecule to the DNA fragment should result in local protection against DNase I digestion. After separating the DNA fragments by gel electrophoresis, the position of the bound compound can be determined by a gap (footprint) in the DNA fragment pattern.

**Figure 4:**
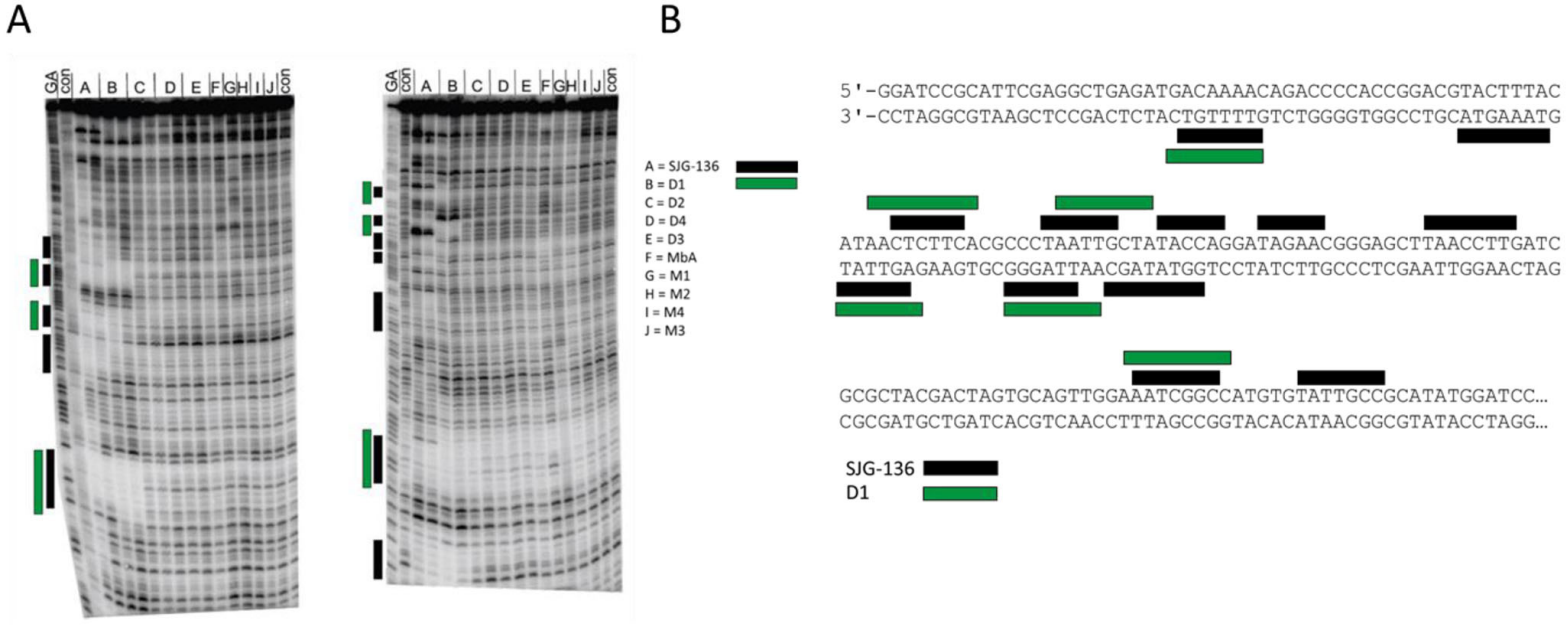
DNase I footprinting of PBDs. A) DNase I cleavage patterns of MS1 (left panel) and MS2 (right panel) in the presence of 1 and 10 µM of each of the dimers and 10 µM for the monomers. A) SJG‐136; B) **D1**; C) **D2**; D) **D4**; E) **D3**; F) **MbA**; G) **M1**; H) **M2**; I) **M4**, and J) **M3**. Lanes labelled “con” show DNase I cleavage without any added compound; lanes labelled “GA” are Maxam‐Gilbert markers specific for purines. The regions protected from DNase I cleavage were identified by visual inspection and are indicated by the boxes on the left‐hand side of the gels (black: SJG‐136 protection; green: **D1** protection. B) Sequences of MS2 (top) and MS1 (bottom) with boxes indicating the regions that are protected from cleavage.

The footprints of the dimers **D1**–**D4**, the monomers **M1**–**M4** and **MbA**, and **SJG‐136** as a control are presented in Figure 4A. The dimers were tested at two different concentrations (1 µm and 10 µm), whereas the monomers were only tested at 10 µm. For all dimers, no difference between the two concentrations was observed. The dimers **D1** and SJG‐136 showed several footprints, as indicated by green and black bars, respectively. Previous studies have shown that different linkers between the two nor‐tomaymycin cores affected the footprinting patterns.^[^ ^26^ ^]^ The footprints of **D1** were similar, but not identical, to those of SJG‐136 (Figure 4B). Even though **D1** produced fewer footprints, all identified regions overlapped with regions protected by SJG‐136, thus showing similar preferences for binding. For the monomers, only the azide **MbA** and the alkyne **M1** showed slight differences compared to the control.

It should be noted that the target duplex concentration in these footprinting reactions is much lower (∼10 nM) than in the DNA melting experiments (200 nM). These compounds bind covalently to their targets, so the observation that some of the duplexes are unaffected (even though there is a large stoichiometric excess of ligand in the melting experiments), must be because of either very slow binding (though the complexes were incubated overnight), or degradation of the ligand at elevated temperatures or detachment of the ligand from DNA during the melting process. In contrast, the concentration of the duplex target is much lower in the footprinting experiments, and do not involve any elevated temperatures.

In summary, three complementary methods, measuring the melting temperature of oligonucleotides, the molecular mass, and DNase I footprinting were used to characterize the binding of the nor‐tomaymycin monomers and dimers that were prepared in this study. All three methods showed that the triazole **D1** acted as a DNA cross‐linker. All three assays showed that the efficiency of cross‐linking decreased in the order SJG‐136 > **D1** > **D2** >> **D3**, **D4**.

### 2.6 Cytotoxicity of the Nor‐tomaymycin Derivatives

The cytotoxicities of the PBD monomers and dimers were evaluated in epithelial‐like mouse melanoma cells (B16F10), mouse colon carcinoma cells (CT26), and in human breast carcinoma epithelial cells (MDA‐MB231) in an 3‐(4,5‐dimethylthiazol‐2‐yl)‐2,5‐diphenyltetrazolium bromide (MTT) assay. For the monomers, the azide **MbA** exhibited a relatively uniform activity with IC_50_ values of 272–570 nM across the three cell lines (Table 2). Among the alkynes, **M1** and **M4** were the most potent, with IC_50_ values of 74 nM and 110 nM in B16F10 cells, respectively. The alkyne **M2**, despite being only one methylene unit longer than **M1**, was less active. Similarly, the cyclooctyne **M3** was less potent than the bicyclic BCN analog, with an IC_50_ of 380 nM in B16F10 cells. Overall, the cytotoxicities of the monomers can be ranked in the order **M1/M4** > **M3 **> **M2**. This trend of cytotoxic activity of the monomers was consistent across the other two cell lines tested (CT26 and MDA‐MB‐231).

**Table 2:** Cellular cytotoxicities of clickable PBD monomers and dimers.

Compound	Cell line; IC_50_ [nM]		
Code	B16‐F10	CT26	MDA‐MB‐231
**MbA**	272 ± 4	570 ± 60	355 ± 1
**M1**	74 ± 50	1200 ± 250	46 ± 14
**M2**	3300 ± 3800	5516 ± >10000	2055 ± 280
**M3**	380 ± 50	2010 ± 10	410 ± 110
**M4**	110 ± 90	312 ± 17	384 ± 36
**D1**	310 ± 240	480 ± 60	5500 ± 3300
**D2**	>10000	2000 ± 140	>10000
**D3**	>10000	700 ± 70	>10000
**D4**	310 ± 90	324 ± 8	260 ± 40
SJG136	0.2 ± 0.04	5.7 ± 1.5	0.1 ± 0.01

The IC_50_ values were derived from sigmoidal dose‐response curves generated from five technical repeats for each dose. For each compound, experiments were performed at least in duplicate. Cells were incubated with compounds for 72 hours at 37 °C before viability was detected.

Among the dimers, **D4** exerted strong and uniform IC_50_ values in the range 260–324 nM across the three cell lines. The dimer **D1** was potent in B16F10 cells and in CT26 cells with IC_50_ values of 310 nM and 480 nM, respectively, but was only weakly active in MDA‐MB231 cells (IC_50_ = 5500 nM). The dimers **D2** and **D3** were found to be less cytotoxic compared to other dimers. All dimers were far less active than the positive control **SJG‐136** which displayed sub‐nM cytotoxic activity across the three cell lines. Notably, the activity ranking **D1/D4** > **D3 **> **D2**, was consistent with that found for the corresponding monomers.

### 2.7 Molecular Modeling Studies

**Figure 5:**
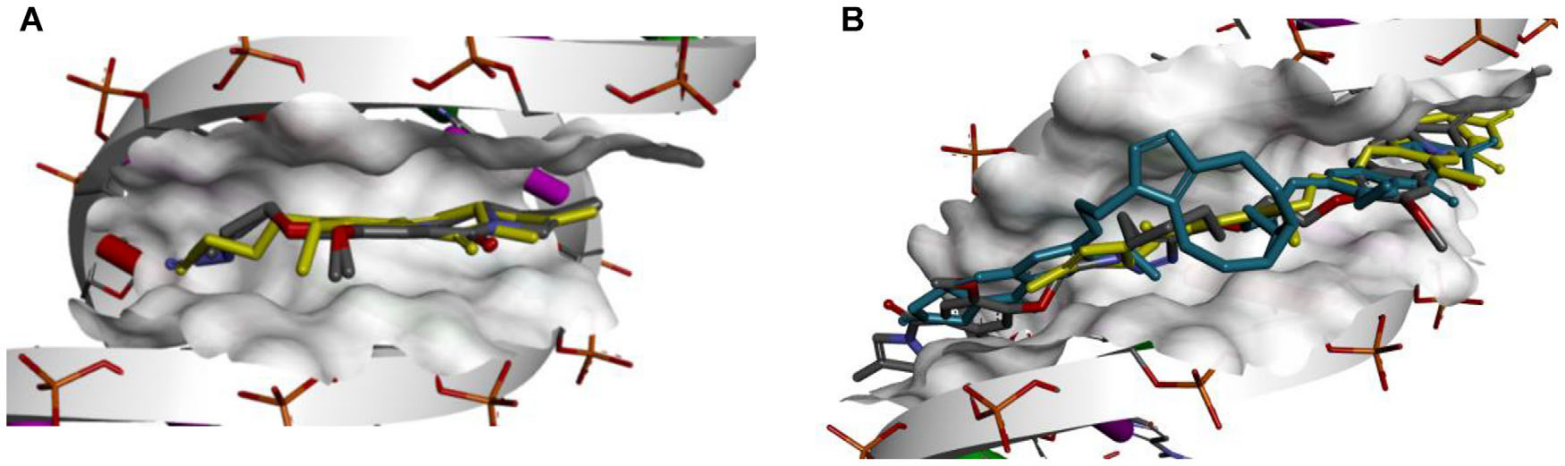
Molecular modeling of binding interactions between nor‐tomaymycin monomers and dimers with DNA (Sequence: 5′‐TATAGGGACAGCGCTATATATAGCGCTGTCCCTATA‐3′). A) Superposition of **M1** (in grey), **M2** (in yellow), and **MbA** (in grey) shows that **M2** folds its alkyne tail moiety within the DNA minor groove differently compared to **M1** and **MbA**. B) Superposition of compounds **D4** (better anticancer activity), **D3** (poor anticancer activity), and reference PBD dimer SJG‐136. **D4** is shown in dark teal, **D3** is shown in grey and SJG‐136 is in yellow.

The interactions between the monomers and dimers with DNA were further studied by molecular modeling with the sequence 5′‐TATAGGGACAGCGCTATATATAGCGCTGTCCCTATA‐3′ to try to rationalize the DNA‐binding characteristics and cytotoxicities observed. The modeling showed that all compounds were able to follow the minor groove of the DNA (Figures 5 and S2) and bind to the DNA sequence with comparable energy values (Table S6). Interestingly, there were subtle differences in the binding interactions of the monomers and the dimers, which help explain the differences in biological activity. For the monomers, the tail of **M2** folds when it interacts with the DNA (Figure 5A), causing a steric clash that may explain the poor activity of **M2** compared to **M1**, which fits snugly within the minor groove. In the case of the dimers, a unique binding event was observed for dimer **D4**. The three‐ring connector system of **D4** remains outside the DNA helices, shortening the distance between the reactive imine groups to 16.9 Å. In contrast, **D3** had an elongated imine‐imine distance of 17.8 Å. This arrangement renders **D4** closer to the optimized dimer SJG‐136 with a distance of 13.8 Å (Figure 5B). The finding is in line with the fact that **D3** was superior to **D4** with respect to its DNA binding capability in the thermal denaturation assay as well as with respect to a higher cytotoxicity. For **D1** and **D2**, mean distances of 14.5 Å and 15.6 Å were calculated.

## 3 Discussion

This study reports synthetic access to PBD dimers through biorthogonal click chemistry of azide‐alkyne pairs. Due to this modular approach, a set of different dimers could be readily obtained from the common nor‐tomaymycin core. In addition, the synthesis of nor‐tomaymycin was modified from the previously published route^[^ ^19^ ^]^ by performing a double deprotection and subsequent cyclization as a one‐pot reaction. This circumvented B‐ring closure via the DIBAL reduction and subsequent nitro group reduction, which proved challenging in our hands.

The DNA‐binding properties of the monomers and dimers were assessed by three independent methods that showed marked differences between the molecules. The DNA thermal denaturation data showed that interaction of the molecules with designed oligonucleotide duplexes led to a mixture of binding events, including mono‐alkylated adducts and, in the case of **D1** and **D2**, DNA cross‐links. The relative proportions of these different species can be estimated from the curve shapes, and it showed that the percentage of compounds bound to the duplex varied between the monomers and dimers. Importantly, both interstrand and intrastrand cross‐links could be identified. Overall, **D1** produced the largest ΔT_m_ and the largest fraction bound to the DNA, and thus the most promising binding and cross‐linking properties, with a preference for the binding motif GAAC suggesting interstrand cross‐linking as the major adduct‐forming mechanism (i.e., similar to the control molecule SJG‐136).

The HPLC‐MS/MS assay provided the most direct, molecular evidence of cross‐linked DNA due to the characteristic and unambiguous molecular mass of two DNA strands covalently bridged by the PBD dimer. The assay provided firm evidence that **D1** produced interstrand cross‐links. These were also formed by **D2** but to a minor extent. However, overall these experiments confirmed that the triazole ring in the center of **D1** as well as **D2** was tolerated within the DNA minor groove. This is consistent with previous reports on PBD dimers with central phenyl‐containing linkers that maintain their biological activity.^[^ ^27^, ^28^ ^]^ On the other hand, the introduction of further steric bulk into the central linker in the form of cyclooctene rings (as in **D3** and **D4**) hindered cross‐linking.

Finally, DNA‐binding was also demonstrated by DNase I footprinting, in which **D1** conferred the clearest protection against enzymatic digestion. For the monomers, the azide **MbA** and the alkyne **M1** only showed slight differences compared to the control, suggesting only weak binding to DNA. Furthermore, the results of the DNA thermal denaturation studies suggested that, for the PBD monomers, a large proportion of duplex was not bound by the compounds even though the ligands were present in a large stoichiometric excess.

The cytotoxicity assays demonstrated that both the clickable monomers and dimers were bioactive in tumor cells. The fact that the activity order of the dimers of **D1**/**D4** > **D3**/**D2** reflected that of the corresponding monomers indicates that the cellular activities, encompassing compound uptake as well as target binding properties, were predetermined by the monomer properties and not significantly perturbed by the triazole formation. A comparison of monomer cytotoxicities with DNA‐binding properties led to two unexpected observations. The cytotoxicity of **M2** was relatively poor despite its significant DNA binding capabilities and close similarity to **M1** in this aspect. The other observation was that the cytotoxicity of **M4** was surprisingly high given its limited DNA‐binding capabilities.

The heterodimerization of **M1**–**M4** to give **D1**–**D4** did not induce a significant increase in cytotoxicity, as observed for SJG‐136 and its component PBD monomer fragments. Instead, the results were highly variable (see Table S5). The very high cytotoxic potency of SJG‐136 is ascribed to its DNA cross‐linking capability, but this correlation does not appear to translate across to the clicked dimers **D1**–**D4**. **D1** showed the clearest cross‐linking capabilities in all three evaluation methods described above, but it was not more cytotoxic compared to its component PBD monomers. On the other hand, **D4** had the poorest DNA cross‐linking characteristics, but its cytotoxicity values across the cell lines examined were among the highest of the set of click dimers investigated. It is conceivable that further targets of PBDs contribute to this lack of correlation, given that SJG‐136 has been reported to also inhibit Src‐associated signaling pathways.^[^ ^29^ ^]^

The molecular modeling studies suggested that the differential folding of the tail of **M2** within the DNA minor groove compared to **M1**, along with the energy penalty from the potential steric clash, could explain the relatively weaker activity of **M2** compared to **M1**. In the case of the dimers, the distance between the reactive groups when the compounds are positioned within the minor groove appears to be more critical than the length of the dimer itself. Thus, the rank order of the distances SJG‐136 < **D1** < **D2** < **D4** < **D3** reflected the inverse order of activity in the thermal denaturation assays, although DNA binding properties could not explain all differences in cellular activities. Overall, the findings from these molecular modeling studies suggest that future research should focus on designing compounds that maintain an optimal distance between key functional groups such as the imines within the minor groove to enhance cytotoxicity. A challenge will be to accommodate optimal linker lengths while retaining the concept of coupling the monomers through click chemistry, which required the incorporation of somewhat longer linkers compared to the reference compound SJG‐136.

While a small set of four heterodimers was prepared in this study to demonstrate feasibility, the respective monomers can serve as building blocks for functionalization with much larger sets of their partners. This modular synthesis approach is not confined to PBD dimers, because it could also facilitate the preparation of mixed dimers with other DNA alkylating agents.^[^ ^30^, ^31^ ^]^ Performing simple CuAAC click reactions with either the azide **MbA** or the alkyne **M1** on the one hand, and another alkylating agent of choice should readily provide access to a larger array of potential cross‐linking agents targeted to different DNA sequence motifs.

The suboptimal cytotoxicity of the molecules produced here indicates that further structural optimization is required. However, because the strained alkynes do not require a catalyst for triazole formation, in principle it should be possible, in principle, to observe the click reaction forming PBD dimers within living cells. This intriguing possibility will form the basis of further studies in this area.^[^ ^32^, ^33^, ^34^ ^]^ If this strategy is successful, PBD monomers which are generally less systemically toxic compared to PBD dimers could be assembled to form highly potent PBD dimers within cancer cells, thus providing a targeting mechanism deployed at the tissue level.

## 4 Conclusion

Dimers of the PBD class belong to one of the most potent families of DNA cross‐linking agents, and these are the active component of a number of antibody drug conjugates, one example of which has already been approved by the FDA and other regulatory authorities. The results of this study demonstrate that PBD homo‐ and heterodimers are accessible through click chemistry, opening an easy modular access to libraries of these compounds. The fact that their DNA‐binding affinity and cytotoxicity is inferior to the PBD dimer SJG‐136 suggests that further structural optimization, including the exploration of novel click partners and lengths between reactive imines, is required. The other intriguing possibility of supplying cells with clickable PBD monomeric molecules and allowing click dimerization to occur intracellularly is presently being investigated, and will be reported elsewhere.

## Author Contributions

JF synthesized the compounds, conducted mass spectrometric experiments, analyzed the data, and wrote the manuscript.

KR contributed to methodology, investigation, and data analysis of mass spectrometric samples.

HLSF analyzed the data and cowrote the manuscript.

MMH conducted the cytotoxicity and molecular modelling experiments.

PR conducted mass spectrometric experiments and analyzed the data.

DET contributed to experimental design, and writing of the manuscript.

KRF conducted the fluorescence melting and DNase I footprinting experiments and analyzed these data.

KMR analyzed the data, supervised the experiments, and cowrote the manuscript.

MB conceptualized and supervised the study, provided funding and wrote the manuscript.

## Conflict of Interest

The authors declare no conflict of interest.

## Supporting information



Supporting Information

## Data Availability

The data that support the findings of this study are available in the supplementary material of this article.
