# Supplementary material for: Synthesis and Evaluation of DNA Cross‐linkers by Click Chemistry‐Mediated Heterodimerization of Nor‐Tomaymycins
Source: Chemistry. 2025 Aug 22;31(53):e01797. doi: 10.1002/chem.202501797 (PMC12451421; doi:10.1002/chem.202501797)

**Synthesis and Evaluation of DNA Cross-linkers by Click Chemistry-Mediated Heterodimerization of Nor-Tomaymycins**

Julia Friederich,^[a]^ Katharina Rox,^[a,b]^ Hazel L. S. Fuchs,^[a]^ Md. Mahbub Hasan,^[c,d]^ Patrick Raunft,^[a]^ David E. Thurston,^[d]^ Keith R. Fox,^[e]^ Khondaker Miraz Rahman,^[d]^ and Mark Brönstrup^*[a,b,f]^

[a] Dr. J. Friederich, Dr. K. Rox, Dr. H.L.S. Fuchs, M. Sc. Patrick Raunft, Prof. Dr. M. Brönstrup

Department of Chemical Biology, Helmholtz Centre for Infection Research, Inhoffenstraße 7, 38124 Braunschweig, Germany

* Email: Mark.Broenstrup@helmholtz-hzi.de

[b] Dr. K. Rox, Prof. Dr. M. Brönstrup

German Center for Infection Research (DZIF), Site Hannover-Braunschweig, Germany

[c] Dr. M.M. Hasan

Department of Genetic Engineering and Biotechnology, Faculty of Biological Sciences, University of Chittagong, Chattogram 4331, Bangladesh

[d] Dr. M.M. Hasan, Prof. Dr. D.E. Thurston, Prof. Dr. K.M. Rahman

Institute of Pharmaceutical Science, School of Cancer & Pharmaceutical Sciences, King's College London, Franklin-Wilkins Building, 150 Stamford Street, London, UK

[e] Prof. Dr. K.R. Fox

School of Biological Sciences, Life Sciences Building 85, University of Southampton, Southampton SO17 1BJ, UK

[f] Prof. Dr. M. Brönstrup

Institute of Organic Chemistry, Leibniz University Hannover, 30167 Hannover, Germany

**Table S1**: Melting temperatures of the fluorescently labelled oligonucleotides incubated with 10 µM of compounds **D1**, **D2**, **D3**, **D4**, **MbA**, **M1**, **M2**, **M3**, **M4** and SJG-136.

|  |  | **SJG-136** | **D1** | **D2** | **D4** | **D3** | **MbA** | **M1** | **M2** | **M3** | **M4** |
| --- | --- | --- | --- | --- | --- | --- | --- | --- | --- | --- | --- |
| **AGA**  T_m_ = 47.0 °C | T_m_^1^ | 67.4 (100) | 66.7 (100) | 45.8 (20) | 47.1 (95) | 47.1 (100) | 46.4 (45) | 46.4 (40) | 46.7 (55) | 47.1 (75) | 47.3 (90) |
|  | T_m_^2^ |  |  | 61.4 (80) | 58.7 (5) |  | 59.2 (55) | 59.9 (60) | 62.0 (45) | 58.0 (25) | 58.8 (10) |
|  | T_m_^3^ |  |  |  |  |  |  |  |  |  |  |
| **GAAG**  T_m_ = 48.8 °C | T_m_^1^ | 68.0 (15) | 47.4 (15) | 48.9 (85) | 48.9 (100) | 48.5 (100) | 48.4 (75) | 48.5 (70) | 48.5 (85) | 48.5 (90) | 48.6 (100) |
|  | T_m_^2^ | 84.1 (85) | 68.2 (35) | 63.9 (15) |  |  | 61.1 (25) | 61.3 (30) | 63.5 (15) | 59.9 (10) |  |
|  | T_m_^3^ |  | 82.4 (50) |  |  |  |  |  |  |  |  |
| **GAAAG**  T_m_ =49.7 °C | T_m_^1^ | 70.2 (40) | 47.7 (15) | 49.8 (80) | 49.8 (100) | 49.7 (100) | 49.3 (50) | 49.3 (55) | 49.9 (95) | 50.0 (80) | 49.8 (80) |
|  | T_m_^2^ | 85.3 (60) | 68.4 (25) | 64.4 (10) |  |  | 62.5 (40) | 63.0 (40) | 61.6  (5) | 61.6 (20) | 61.6 (20) |
|  | T_m_^3^ |  | 82.7 (60) | 78.1 (10) |  |  | 73.5 (10) | 74.6 (5) |  |  |  |
| **GAAAAG**  T_m_ = 46.6 °C | T_m_^1^ | 85.5 (100) | 44.4 (2) | 45.2 (10) | 46.4 (90) | 46.4 (100) | 46.2 (45) | 46.2 (30) | 45.9 (30) | 46.5 (75) | 46.6 (90) |
|  | T_m_^2^ |  | 65.0 (18)* | 60.7 (70) | 57.7 (10) |  | 58.9 (40) | 59.3 (50) | 61.0 (65) | 58.2 (20) | 57.7 (10) |
|  | T_m_^3^ |  | 82.4 (80) | 73.1 (20) |  |  | 71.0 (15) | 72.1 (20) | 73.3 (5) |  |  |
| **GAAC**  T_m_ = 50.6 °C | T_m_^1^ | 48.7 (5) | 69.9 (20) | 50.2 (75) | 50.0 (65) | 50.7 (100) | 49.6 (15) | 49.8 (25) | 50.0 (45) | 50.8 (55) | 50.5 (85) |
|  | T_m_^2^ | 68.9 (15) | 81.6 (80) | 64.0 (25) | 62.1 (35) |  | 62.0 (35) | 62.4 (45) | 64.5 (55) | 61.8 (45) | 61.6 (15) |
|  | T_m_^3^ | 81.5 (80) |  |  |  |  | 71.8 (50) | 73.3 (30) |  |  |  |
| **GAAAC**  T_m_ = 50.0 °C | T_m_^1^ | 48.3 (1) | 47.4 (1) | 48.9 (20) | 49.9 (80) | 49.8 (100) | 49.7 (25) | 49.7 (20) | 49.4 (70)* | 50.3 (60) | 50.3 (80) |
|  | T_m_^2^ | 68.8 (9) | 68.4 (29) | 63.0 (80) | 60.7 (20) |  | 61.2 (50) | 61.8 (55) | 63.8 (30) | 60.5 (30) | 61.4 (20) |
|  | T_m_^3^ | 88.2 (90) | 80.5 (79) |  |  |  | 71.1 (30) | 72.9 (25) |  | 68.4 (10) |  |
| **GAAAAC**  T_m_ = 48.1 °C | T_m_^1^ | 67.8 (10) | 66.5 (30) | 46.3 (10) | 47.6 (90) | 47.7 (100) | 47.6 (60) | 46.9 (25) | 47.3 (65) | 47.7 (100) | 48.0 (95) |
|  | T_m_^2^ | 86.1 (90) | 84.2 (70) | 60.8 (55) | 59.0 (10) |  | 59.6 (30) | 59.8 (50) | 61.9 (30) |  | 59.6 (5) |
|  | T_m_^3^ |  |  | 73.1 (35) |  |  | 69.3 (10) | 71.4 (25) | 72.5 (5) |  |  |

T_m_^1^, T_m_^2^ and T_m_^3^ correspond to the melting temperatures of the first, second and third transition. Values in parentheses correspond to the proportion (in %) of the transition by the free DNA and the first and second melting transition respectively. Percentages are rounded in 5% increments to the nearest value. * indicates that the transition is broad and could therefore not be precisely resolved.

**Table S2**: Melting temperatures of the fluorescently labelled oligonucleotides incubated with compounds **D1**, **D2**, **M1**, **M2**, **MbA** as well as the two combinations **MbA** + **M1** and **MbA** + **M2**.

|  |  | **D1** | **MbA** | **M1** | **MbA**  **+ M1** | **D2** | **MbA** | **M2** | **MbA**  **+ M2** |
| --- | --- | --- | --- | --- | --- | --- | --- | --- | --- |
| **GAAG**  T_m_ =48.8 °C | T_m_^1^ | 47.4 (15) | 48.4 (75) | 48.5 (70) | 46.2 (72) | 48.9 (85) | 48.4 (75) | 48.5 (85) | 45.4 (80) |
|  | T_m_^2^ | 68.2 (35) | 61.1 (25) | 61.3 (30) | 59.3 (18) | 63.9 (15) | 61.1 (25) | 63.5 (15) | 58.9 (20) |
|  | T_m_^3^ | 82.4 (50) |  |  |  |  |  |  |  |
| **GAAAG**  T_m_ =49.7 °C | T_m_^1^ | 47.7 (15) | 49.3 (50) | 49.3 (55) | 47.1 (55) | 49.8 (80) | 49.3 (50) | 49.9 (95) | 47.4 (75) |
|  | T_m_^2^ | 68.4 (25) | 62.5 (40) | 63.0 (40) | 60.4 (35) | 64.4 (10) | 62.5 (40) | 61.6  (5) | 60.5 (25) |
|  | T_m_^3^ | 82.7 (60) | 73.5 (10) | 74.6 (5) | 71.3 (10) | 78.1 (10) | 73.5 (10) |  |  |
| **GAAC**  T_m_ =50.6 °C | T_m_^1^ | 69.9 (20) | 49.6 (15) | 49.8 (25) | 48.3 (70) | 50.2 (75) | 49.6 (15) | 50.0 (45) | 48.4 (85) |
|  | T_m_^2^ | 81.6 (80) | 62.0 (35) | 62.4 (45) | 60.8 (20) | 64.0 (25) | 62.0 (35) | 64.5 (55) | 60.9 (15) |
|  | T_m_^3^ |  | 71.8 (50) | 73.3 (30) | 68.9 (10) |  | 71.8 (50) |  |  |
| **GAAAC**  T_m_ =50.0 °C | T_m_^1^ | 47.4 (1) | 49.7 (25) | 49.7 (15) | 48.0 (70) | 48.9 (20) | 49.7 (25) | 49.4 (70)* | 47.8 (85) |
|  | T_m_^2^ | 68.4 (20) | 61.2 (50) | 61.8 (60) | 60.7 (25) | 63.0 (80) | 61.2 (50) | 63.8 (30) | 60.0 (15) |
|  | T_m_^3^ | 80.5 (79) | 71.1 (25) | 72.9 (25) | 68.4 (5) |  | 71.1 (25) |  |  |

T_m_^1^, T_m_^2^ and T_m_^3^ correspond to the melting temperatures of the first, second and third transition. Values in parentheses correspond to the proportion (in percent) of the transition that is represented by the free DNA, first and second melting transition respectively. Percentages are rounded in 5% increments to the nearest value. * indicates that the transition is broad and could therefore not be precisely resolved.

**Fluorescence melting**

In these studies, one DNA strand contained an attached 5’-fluorescein (F), while its complement contained 3’-dabcyl (Q) as a fluorescence quencher. All oligonucleotides were purchased from atdbio, Southampton, UK. The F- and Q-labelled oligonucleotide pairs were annealed at a concentration of 0.25 µM in 50 mM sodium phosphate buffer pH 7.4 containing 0.2 M NaCl. The mixture was heated at 90 °C for 1 min and slowly cooled to room temperature. 4 µL of the ligand was then added to 16 µL of the annealed oligonucleotides to achieve a final ligand concentration of 0, 10 µM, and the mixture was incubated for 18 hours at room temperature. Fluorescence melting profiles were measured using a Roche LightCycler. The samples were denatured by heating to 95 °C at a rate of 1 °C min^-1^ and the changes in fluorescence were measured at 520 nm. Tm values were obtained from the first derivates of the melting profiles using the Roche LightCycler software.

Data analysis:

The fluorescence melting data were normalized, with 1 representing the fluorescence measured at 95°C and 0 the fluorescence at 31°C. The normalized data were plotted as shown in Figure 2. The inflection points were determined mathematically by fitting a trend line using GraphPadPrism with the equation below (R^2^ >0.999). Data points beyond the maximum fluorescence were excluded from the analysis.


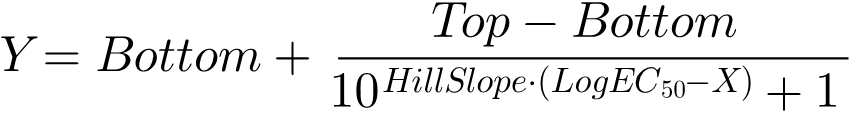


To analyze multiple transitions, the data set was manually divided to give several single sigmoidal curves, and the curve fitting was performed for each single sigmoidal curve. The heights of the individual curves were used to determine the approximate proportions of the various transitions in %.

**LC-MS-Assay for the detection of DNA adducts**

**Sample incubation**

Equal volumes of dsDNA working stock (50 μm) and the investigated compound (200 μm) were mixed and incubated at 37 °C for 16 h. For subsequent LC-MS measurements, the samples were diluted to a final dsDNA concentration of 500 nm.

**Liquid chromatography**

| Agilent Integrated System: | Binary Pump G7120A  Binary Pump G7120A  Multisampler G7167B  Column Comp. G7116B |
| --- | --- |
| Column: | Zorbax eclipse plus C18 RRHD 1.8 µm 2.1 x 50 mm |
| Injection volume | 10 µL |
| Solvent A: | 100.0 % Water  + 15 mm TEAB + 100 mm HFIP |
| Solvent B: | 90% ACN 10% Water  + 15 mm TEAB + 100 mm HFIP |

Gradient:

| **Time (min)** | **A (%)** | **B (%)** | **Flow (mL/min)** |
| --- | --- | --- | --- |
| 1 | 99 | 1 | 0.7 |
| 2 | 99 | 1 | 0.7 |
| 5 | 40 | 60 | 0.7 |
| 7 | 5 | 95 | 0.7 |
| 8 | 99 | 1 | 0.7 |

**Mass Spectrometry**

General:

| Mass spectrometer: | Triple Quad 7500 HighMass (AB Sciex) |
| --- | --- |
| Software: | SCIEX OS 2.1.6.59781 |
| Method duration (minutes): | 8 |
| Total scan time (s): | 1.079 |
| Estimated cycles: | 445 |
| Actual method duration (minutes): | 8.87 |

Ion source:

| Source name: | E-ANLYT 200+ µL |
| --- | --- |
| Curtain gas (psi): | 55 |
| Ion source gas 1 (psi): | 80 |
| Ion source gas 2 (psi): | 70 |
| Temperature (°C): | 400 |

Experiment:

| Scan type: | MRM |
| --- | --- |
| Polarity: | Negative |
| Ionspray voltage (V): | 3500 |
| Q1 resolution: | Unit |
| CAD gas: | N_2_ |
| Q3 resolution: | Unit |
| Pause time (ms): | 2 |
| Settling time (ms): | 5 |
| High mass cooling time (ms): | 0 |
| Q0 dissociation: | False |

**Table S3**: List of most prominent^a)^ multiple reaction monitoring (MRM) transitions showing compound binding and cross-link formation for **SJG-136**, **D1** and **D2**.

|  | Precursor ion | Description | Product ion | Description |
| --- | --- | --- | --- | --- |
| SJG-136 | 1119.0 | [dsDNA+SJG-136]^7-^ | 909.6 | [ssDNA]^4-^ |
|  | 1568.0 | [dsDNA+SJG-136]^5-^ | 1213.2 | [ssDNA]^3-^ |
|  | 1959.5 | [dsDNA+SJG-136]^4-^ | 1820.6 | [ssDNA]^2-^ |
| D1 | 1128.9 | [dsDNA+D1]^7-^ | 909.6 | [ssDNA]^4-^ |
|  | 1421.0 | [ssDNA+D1]^3-^ | 1213.1 | [ssDNA]^3-^ |
|  | 1580.0 | [dsDNA+D1]^5-^ | 1213.2 | [ssDNA]^3-^ |
|  | 1976.0 | [dsDNA+D1]^4-^ | 1212.9 | [ssDNA]^3-^ |
| D2 | 1425.6 | [ssDNA+D2]^3-^ | 1213.1 | [ssDNA]^3-^ |
|  | 1583.9 | [dsDNA+D2]^5-^ | 1213.2 | [ssDNA]^3-^ |
|  | 1979.2 | [dsDNA+D2]^4-^ | 1820.2 | [ssDNA]^2-^ |

a) Complete list of MRMs used for detection provided in Table S4.


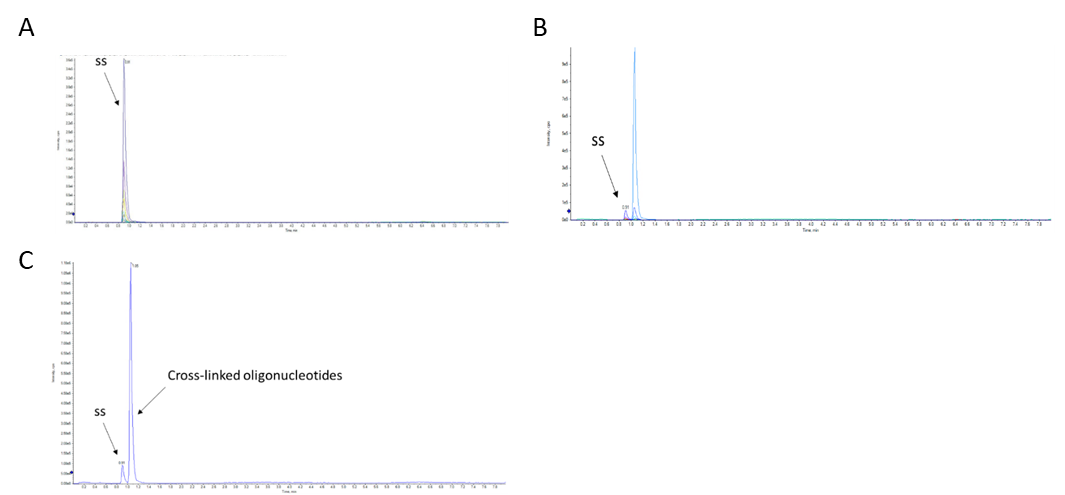


**Figure S1**: LC-MS/MS assay showing DNA cross-linking by SJG-136. Different MRM transitions are depicted in different colors. A: Individual MRMs of untreated double strand (ds) DNA, serving as a negative control; here only single strand DNA (ss) and no dsDNA was detected due to the denaturing conditions during the HPLC separation. B and C: oligonucleotides cross-linked with SJG-136 (right peak) and free oligonucleotides (left peak), displayed as individual MRMs (B) and as Total ion count (TIC) of all MRMs (C).

**Table S4**: List of all MRM transitions used for detection.

| **Group ID** | **Q1 mass** | **Q3 mass** | **Dwell time** | **EP** | **CE** | **CXP** |
| --- | --- | --- | --- | --- | --- | --- |
| **JF-Seq-1** | 1820.22 | 1331.227 | 47.556 | -10 | -72 | -24 |
|  | 1820.22 | 1017.968 | 47.556 | -10 | -71 | -34 |
|  | 1454.52 | 1213.26 | 47.556 | -10 | -35 | -22 |
|  | 1454.52 | 1256.727 | 47.556 | -10 | -11 | -7 |
|  | 1213.36 | 634.059 | 47.556 | -10 | -55 | -18 |
|  | 1213.36 | 922.028 | 47.556 | -10 | -46 | -16 |
|  | 1213.36 | 921.656 | 47.556 | -10 | -42 | -16 |
|  | 1213.36 | 1167.775 | 47.556 | -10 | -32 | -12 |
|  | 1213.36 | 480.988 | 47.556 | -10 | -61 | -13 |
|  | 909.9 | 78.946 | 47.556 | -10 | -179 | -11 |
|  | 909.9 | 820.3 | 47.556 | -10 | -35 | -26 |
|  | 909.9 | 481.056 | 47.556 | -10 | -44 | -19 |
|  | 728.0 | 700.767 | 47.556 | -10 | -20 | -8 |
|  | 728.0 | 78.955 | 47.556 | -10 | -175 | -6 |
|  | 728.0 | 303.107 | 47.556 | -10 | -41 | -6 |
| **SJG-136** | 1119.0 | 909.63 | 40 | -10 | -21 | -13 |
|  | 1119.0 | 451.916 | 40 | -10 | -36 | -23 |
|  | 1305.0 | 1242.93 | 40 | -10 | -11 | -15 |
|  | 1305.0 | 357.333 | 40 | -10 | -55 | -6 |
|  | 1568.0 | 1213.259 | 40 | -10 | -36 | -22 |
|  | 1568.0 | 363.116 | 40 | -10 | -65 | -16 |
|  | 1959.5 | 1820.608 | 40 | -10 | -38 | -32 |
|  | 1959.5 | 1332.116 | 40 | -10 | -82 | -21 |
| **D1** | 1128.9 | 909.567 | 40 | -10 | -23 | -21 |
|  | 1128.9 | 880.2 | 40 | -10 | -44 | -22 |
|  | 1421.0 | 1213.074 | 40 | -10 | -34 | -24 |
|  | 1421.0 | 1212.472 | 40 | -10 | -31 | -28 |
|  | 1580.0 | 1213.193 | 40 | -10 | -37 | -20 |
|  | 1580.0 | 1212.407 | 40 | -10 | -39 | -28 |
|  | 1976.0 | 1212.973 | 40 | -10 | -58 | -47 |
|  | 1976.0 | 1819.869 | 40 | -10 | -43 | -28 |
| **D2** | 1068.5 | 992.265 | 40 | -10 | -13 | -53 |
|  | 1068.5 | 97.052 | 40 | -10 | -148 | -9 |
|  | 1425.6 | 1213.113 | 40 | -10 | -33 | -26 |
|  | 1425.6 | 679.276 | 40 | -10 | -43 | -20 |
|  | 1583.9 | 1214.174 | 40 | -10 | -36 | -17 |
|  | 1583.9 | 1213.293 | 40 | -10 | -32 | -19 |
|  | 1979.2 | 1820.179 | 40 | -10 | -41 | -28 |
|  | 1979.2 | 1196.307 | 40 | -10 | -82 | -23 |
| **D4** | 733.4 | 566.256 | 20 | -10 | -17 | -10 |
|  | 733.4 | 56.952 | 20 | -10 | -109 | -4 |
|  | 733.4 | 86.844 | 20 | -10 | -94 | -7 |
|  | 733.4 | 58.924 | 20 | -10 | -100 | -6 |
|  | 733.4 | 68.889 | 20 | -10 | -112 | -10 |
|  | 1467.4 | 1213.147 | 20 | -10 | -28 | -20 |
|  | 1467.4 | 1214.694 | 20 | -10 | -28 | -53 |
|  | 1467.4 | 1338.3 | 20 | -10 | -13 | -10 |
|  | 1467.4 | 923.314 | 20 | -10 | -56 | -15 |
|  | 1467.4 | 489.46 | 20 | -10 | -73 | -14 |
| **D3** | 1884.3 | 1819.997 | 40 | -10 | -32 | -18 |
|  | 1884.3 | 1213.337 | 40 | -10 | -57 | -22 |
|  | 1949.5 | 1820.418 | 40 | -10 | -34 | -25 |
|  | 1949.5 | 1331.135 | 40 | -10 | -78 | -20 |
|  | 1957.3 | 1820.319 | 40 | -10 | -38 | -26 |
|  | 1957.3 | 1893.065 | 40 | -10 | -12 | -12 |
|  | 1892.6 | 1820.103 | 40 | -10 | -34 | -25 |
|  | 1892.6 | 1251.054 | 40 | -10 | -84 | -23 |
| **MbA + M1** | 1312.9 | 1213.701 | 40 | -10 | -24 | -21 |
|  | 1312.9 | 207.119 | 40 | -10 | -127 | -10 |
|  | 1322.1 | 1213.174 | 40 | -10 | -25 | -20 |
|  | 1322.1 | 1259.635 | 40 | -10 | -15 | -16 |
|  | 1831.5 | 1213.07 | 40 | -10 | -51 | -40 |
|  | 1831.5 | 506.088 | 40 | -10 | -128 | -19 |
|  | 1893.1 | 1820.454 | 40 | -10 | -34 | -27 |
|  | 1893.1 | 1331.136 | 40 | -10 | -74 | -48 |
|  | 1903.7 | 1831.272 | 40 | -10 | -40 | -25 |
|  | 1903.7 | 1213.163 | 40 | -10 | -54 | -18 |
|  | 1968.5 | 1831.375 | 40 | -10 | -38 | -28 |
|  | 1968.5 | 1331.151 | 40 | -10 | -83 | -30 |
|  | 1983.9 | 1820.344 | 40 | -10 | -38 | -12 |
|  | 1983.9 | 1212.929 | 40 | -10 | -52 | -38 |
| **MbA + M2** | 1884.6 | 1820.193 | 40 | -10 | -35 | -25 |
|  | 1884.6 | 1331.13 | 40 | -10 | -75 | -22 |
|  | 1892.5 | 1820.01 | 40 | -10 | -31 | -27 |
|  | 1892.5 | 793.465 | 40 | -10 | -100 | -12 |
|  | 1949.6 | 1820.463 | 40 | -10 | -36 | -23 |
|  | 1949.6 | 1331.154 | 40 | -10 | -84 | -23 |
|  | 1957.5 | 1892.967 | 40 | -10 | -11 | -22 |
|  | 1957.5 | 1820.294 | 40 | -10 | -34 | -26 |
| **MbA + M4** | 1983.7 | 1212.842 | 100 | -10 | -55 | -20 |
|  | 1983.7 | 1213.55 | 100 | -10 | -58 | -41 |
|  | 1983.7 | 1830.657 | 100 | -10 | -46 | -42 |
|  | 1983.7 | 401.508 | 100 | -10 | -105 | -31 |
|  | 1983.7 | 1227.194 | 100 | -10 | -65 | -49 |
| **MbA + M3** | 1983.9 | 1213.602 | 100 | -10 | -53 | -55 |
|  | 1983.9 | 1819.731 | 100 | -10 | -41 | -34 |
|  | 1983.9 | 385.3 | 100 | -10 | -95 | -28 |
|  | 1983.9 | 1169.82 | 100 | -10 | -51 | -31 |
|  | 1983.9 | 551.643 | 100 | -10 | -82 | -23 |
|  | 1459.1 | 1213.341 | 100 | -10 | -27 | -17 |
|  | 1459.1 | 1212.66 | 100 | -10 | -31 | -25 |
|  | 1459.1 | 1078.15 | 100 | -10 | -54 | -30 |
|  | 1459.1 | 1169.708 | 100 | -10 | -17 | -21 |
|  | 1459.1 | 1395.312 | 100 | -10 | -11 | -19 |
|  | 1322.3 | 1213.235 | 100 | -10 | -20 | -21 |
|  | 1322.3 | 1212.478 | 100 | -10 | -21 | -55 |
|  | 1322.3 | 1116.091 | 100 | -10 | -71 | -34 |
|  | 1322.3 | 94.909 | 100 | -10 | -164 | -7 |
|  | 1322.3 | 243.2 | 100 | -10 | -114 | -16 |
|  | 1349.6 | 1220.096 | 100 | -10 | -17 | -17 |
|  | 1349.6 | 1212.744 | 100 | -10 | -21 | -47 |
|  | 1349.6 | 1017.824 | 100 | -10 | -61 | -41 |
|  | 1349.6 | 1213.417 | 100 | -10 | -21 | -14 |
|  | 1349.6 | 305.295 | 100 | -10 | -83 | -12 |

**DNase I footprinting**

DNase I footprinting was performed using the DNA fragments MS1 and MS2 that contain all possible 136 tetranucleotide sequences. These two fragments contain the same sequence, but were cloned in opposite orientations, so that sequences at the top of the gel for MS1 are near the bottom for MS2, and vice versa. The DNA fragments were obtained by cutting the parent plasmids with HindIII and SacI and were labelled at the 3'-end of the HindIII site with [α-^32^P]dATP using exo- Klenow fragment. After gel purification, the radiolabelled DNA was dissolved in 10 mM Tris-HCl pH 7.5 containing 0.1 mM EDTA, at a concentration of about 10 c.p.s per μL as determined on a handheld Geiger counter. 1.5 μL of radiolabelled DNA was mixed with 1.5 μL ligand that had been freshly diluted at the desired concentrations in Tris-HCl pH 7.5, containing 10 mM NaCl. The complexes were left to equilibrate overnight before digesting with 2 μL DNase I (final concentration about 0.01 units/mL in a solution containing 20 mM NaCl, 2 mM MgCl_2_ and 2 mM MnCl_2_). The reactions were stopped after 1 minute by adding 4 μL of formamide containing 10 mM EDTA and bromophenol blue (0.1% w/v). The samples were then heated at 100 °C for 3 minutes before loading onto 8% denaturing polyacrylamide gels containing 8 M urea. Gels were fixed in 10% acetic acid, transferred to 3MM paper, dried and exposed to a phosphor screen overnight, before analysing with a Typhoon phosphorimager.

**Cytotoxicity Assay**

Cytotoxicity screening of the synthesized drugs was conducted using three different cell lines: B16-F10, CT26, and MDA-MB-231. For culturing cells, RPMI media (Gibco™ 31870025) supplemented with 10% v/v Newborn Calf Serum (Gibco™ 16010159), 1% v/v GlutaMAX™ (Gibco™ 35050061), and 1% v/v Penicillin-Streptomycin (10,000 U/mL) (Gibco™ 15140122) was used for B16-F10 and CT26. For MDA-MB-231, we used Advanced RPMI 1640 (Gibco™ 12633012), while the other supplements remained the same. We seeded 5,000 live cells per well. The seeded cells were allowed to settle into the plates overnight at 37 °C with 5% CO₂. The next day, the media was discarded and refilled with fresh media for the respective cell lines containing different drug concentrations.

For each drug concentration, five wells were treated as technical repeats. The treated plates were incubated for 72 hours at 37 °C with 5% CO₂. After incubation, the drug-containing media was discarded, and the cells in the wells were treated with 0.1 mg 3-(4,5-dimethylthiazol-2-yl)-2,5-diphenyltetrazolium bromide (MTT) per well in 100 µL of the respective media for the cell line. The plates were incubated for 4 hours at 37 °C with 5% CO₂. The media containing MTT was carefully removed. Formazan, the reduced form of MTT converted by cellular mitochondrial reductase, was dissolved in 200 µL of DMSO and shaken for 5 minutes on a rotary shaker to dissolve completely. Finally, the absorbance of the plates was measured in a plate reader at 570 nm.

From the absorbance data, we calculated the mean percentage of cell viability for each concentration of the tested drug and the standard deviation (SD). The computed mean and SD values were then plotted in GraphPad Prism 9.5.1. The IC_50_ value for each tested compound was calculated by transforming the data into log concentrations, followed by the nonlinear regression model: log(inhibitor) vs. response (three parameters).

**Table S5**: Relative cytotoxicities of PBD dimers compared to their monomers. Values <1 indicate more potent cytotoxicities of the dimer.^a)^

| **Dimer** vs. **Monomer pair** | **Relative potencies** | | |
| --- | --- | --- | --- |
|  | **B16F10** | **CT26** | **MDA-MB231** |
| **D1** vs **M1/MbA** | 1,8 | 0,5 | 27,4 |
| **D2** vs **M2/MbA** | >5,5 | 0,7 | >8,3 |
| **D3** vs **M3/MbA** | >30,8 | 0,5 | >26,0 |
| **D4** vs **M4/MbA** | 1,6 | 0,7 | 0,7 |

a) Calculated as EC_50_ of the dimer divided by the average EC_50_ of the monomers in three different cell lines.

**Molecular Docking**

The 3D structures of the desired B-form DNA sequence (5'-TATAGGGACAGCGCTATATATAGCGCTGTCCCTATA-3') were generated using the PyMOL 2.5 Structure Builder. For the small molecule ligands used in this study, 3D structures were generated using the Chem3D 20.0 program. The DNA structures were processed (energy minimization and addition of polar hydrogens) using MGLTools v1.5.7 (https://autodock.scripps.edu/). The grid box was configured for each DNA macromolecule to cover the entire length of the structure so that the ligand could find the best possible binding sites along the DNA structures, including both the major and minor grooves. The small molecule ligands were also processed with the same tools. Finally, molecular docking was performed using the open-source AutoDock Vina v.1.2.0 (https://vina.scripps.edu/). The default flexible docking parameters were used (exhaustiveness 100). The post-processing of the output files was curated using PyMOL 2.5, and the molecular interactions were visualized using BIOVIA Discovery Studio Visualizer.

The PDB files with the key coordinates of binding interactions are deposited as supplementary files.

**Table S6:** Binding energies of nortomaymycin monomers and dimers to DNA.^a^

| **Compound** | **Binding affinity (kcal/mol)** |
| --- | --- |
| MbA | -8.9 |
| M1 | -8.5 |
| M2 | -8.3 |
| M3 | -9.7 |
| M4 | -9.4 |
| D1 | -12.4 |
| D2 | -12.6 |
| D3 | -14 |
| D4 | -11.7 |

a) DNA sequence (5'-TATAGGGACAGCGCTATATATAGCGCTGTCCCTATA-3')

**Figure S2:** Molecular modeling of binding interactions between nor-tomaymycin monomers and dimers with DNA. DNA minor groove fitting of monomers M3 (A) and M4 (B) is presented superposed with MbA, while dimers **D1** (C) and **D2** (D) are presented superposed with SJG-136. **M3**, **M4**, **D1** and **D2** are shown in yellow, and **MbA** and SJG-136 are kept in grey colour. The monomers **M3** and **M4** fitted within the DNA minor groove, with their C8 substituents positioned as overhangs. The dimer **D1** fitted within the groove by adjusting its linker between the two PBD core components, while **D2** was found to have positional variation to ensure its fit within the DNA minor groove.

**Chemical synthesis**

**Materials and Methods**

Unless stated otherwise, all reagents were purchased from commercial suppliers and used without further purification. All solvents used for workup and purification were of HPLC grade. Anhydrous solvents were used for all reactions in which water was not also used as a solvent and in which the total amount of organic solvent did not exceed 100 mL. All anhydrous solvents were purchased from commercial suppliers. Moisture-sensitive reactions were performed under argon atmosphere in dried glassware. Reactions were monitored by TLC, LC-MS or NMR. The removal of organic solvents took place using rotary evaporators at 30 °C, the removal of water at 40 °C. For lyophilization of substances, the solutions in question were frozen with liquid nitrogen and freeze-dried on an Alpha 2-4 LSCbasic (Christ) instrument. Centrifugations were performed using a Universal 32 R centrifuge (Hettich).

Column chromatographic purifications were carried out on silica gel (Si 60, 40 - 63 µm particle size) from the producer Merck under elevated pressure (flash chromatography). The eluents used are listed after the indicated retention factors.

Automatic preparative column chromatography was carried out on a Grace Reveleris® X2 instrument (Büchi).

Automatic preparative column chromatography with C18 Silica was performed on a Pure C-850 instrument (Büchi).

Purifications by HPLC were performed on a Dionex Ultimate instrument (Thermo Fisher Scientific). Unless stated otherwise, the following C18 separation columns (Phenomenex) were used:

- Luna 5 μm, 100 Å, 00G-4252-PO-AX.
- Gemini 10 μm, 110 Å, 00G-4436-PO.
- Gemini 10 μm, 110 Å, 00G-4436-NO.

Product containing fractions were combined, diluted with milliQ H_2_O (min. 1:1/solvent:H_2_O), frozen and lyophilized.

Preparative thin layer chromatography was performed on pre-coated glass plates (Merck TLC Silicagel 60 F254, 1.05715.0001, 20×20 cm, max. 10-15 mg/plate and Analtech Uniplate Silica gel GF Z51305-9, 20×20 cm × 2 mm, max 100-150 mg/plate). Eluent or eluent-mixtures used are reported in parentheses. Compounds were visualized by observation under UV light (λ = 254 or 366 nm). Compound containing silica gel fractions were scratched from the plate with a scalpel, crushed to small pieces and compounds were dissolved by appropriate solvent mixtures.

Nuclear Magnetic Resonance (NMR) spectra were recorded on a Bruker Avance III 500 with the probe head PABBO BB/^19^F-^1^H/D Z-GRD (500 MHz for ^1^H, 125 MHz for ^13^C) or a Bruker Avance III HD 700 with the probe head CPTCI ^1^H-^13^C/^15^N/D Z-GRD (700 MHz for ^1^H, 176 MHz for ^13^C) at room temperature. Samples were measured as solutions in deuterated solvents. Chemical shifts are reported in ppm relative to solvent signal. Multiplicity is indicated as follows: s (singlet); bs (broad singlet); d (doublet); t (triplet); q (quartet); quin (quintet), m (multiplet); as well as combination of those e.g. dd (doublet of doublets), etc.

Low resolution mass spectrometry (LRMS) data were recorded using an Agilent 1100 HPLC system equipped with DAD detector and connected to an Agilent 6130 quadrupole mass detector with electrospray ionization (ESI) (ACN-H_2_O + 0.05 % TFA)

High resolution mass spectrometry (HRMS) data were recorded using a Dionex Dionex Ultimate 3000 HPLC system equipped with a DAD detector and a Bruker maXis HD QTOF mass detector with electrospray ionization (ESI). Samples were directly injected via an Ultimate 3000RS autosampler (Thermo Fisher Scientific). The mass-to-charge ratio *m/z* is being reported.

**Synthetic procedures**

**(*S*)-2-(methoxycarbonyl)-4-methylenepyrrolidiniumchloride (5)**

To 1-(*tert*-butyl) 2-methyl (*S*)-4-methylenepyrrolidine-1,2-dicarboxylate (169 mg, 0.70 mmol, 1.0 eq.) was added 0.79 mL of HCl (4 m in dioxane). The solution was stirred at RT for 1 h. Et_2_O was added and the precipitate was separated by centrifugation of the mixture. The resulting pellet was washed twice with Et_2_O. The remaining solvent residues were removed under reduced pressure to obtain the analytically pure product as a colorless solid (124 mg, 0.70 mmol, 99.7%).**^1^H NMR** (500 MHz, DMSO): δ [ppm] = 5.16 (sx, *J* = 2.1 Hz, 2H), 4.60 (t, *J* = 8.4 Hz, 1H), 3.86 (td, *J* = 30.9, 1.5 Hz, 2H), 3.77 (s, 3H), 3.57 (s, 1H), 2.99 – 2.69 (m, 2H). **^13^C NMR** (126 MHz, DMSO): δ [ppm] = 168.5, 139.8, 109.8, 66.3, 58.3, 53.0, 48.6, 33.2.

**4-(benzyloxy)-3-methoxybenzoic acid (6)**

Vanillic acid (25.0 g, 148 mmol, 1.0 eq.) was dissolved in THF (75 mL), NaOH (188 mL, 2 m) was added and the stirred mixture was cooled down to 0 °C. A solution of benzyl bromide in THF (75 mL) was added dropwise at 0 °C over 1 h. The mixture was allowed to warm up to RT and was subsequently stirred under reflux overnight. The reaction was monitored by LC-MS. When no starting material remained, the mixture was allowed to cool to RT and was washed with hexane (2 × 50 mL). Remaining THF was removed under reduced pressure. The remaining aqueous phase was acidified to pH = 1 with HCl (6 m). The white participate was collected by centrifugation. The pellet was washed with water. After removing remaining water by lyophilization of the pellet, the product was obtained as an analytically pure colorless solid (35.1 g, 135.8 mmol, 91%).**TLC**: *R*_f_ = 0.59 (PE:EtOAc 5:1, HOAc) [UV] **^1^H NMR** (500 MHz, DMSO): δ [ppm] = 12.68 (s, 1H), 7.54 (dd, *J* = 8.3, 2.0 Hz, 1H), 7.46 – 7.45 (m, 3H), 7.40 (t, *J* = 7.5 Hz, 2H), 7.35 (t, *J* = 7.3 Hz, 1H), 7.14 (d, *J* = 8.5 Hz, 1H), 5.16 (s, 2H), 3.81 (s, 3H). **^13^C NMR** (126 MHz, DMSO): δ [ppm] = 167.0, 151.6, 148.6, 136.5, 128.4, 128.0, 127.9, 123.2, 123.0, 112.4, 112.1, 69.8, 55.5. **HRMS** (ESI) *m/z*: (C_15_H_13_O_4_^-^ [M-H]^-^) calc.:257.0819, found: 257.0813.

**4-(benzyloxy)-5-methoxy-2-nitrobenzoic acid (7)**

4-(benzyloxy)-3-methoxybenzoic acid (7.64 g, 29.6 mmol, 1.0 eq.) was dissolved in HNO_3_ (65%, 100 mL) at 0 °C. The mixture was allowed to warm to room temperature and stirred for 2 h. The reaction mixture was poured on ice followed by extraction with DCM. The combined organic layers were dried over Na_2_SO_4_ and concentrated under reduced pressure. 4-(benzyloxy)-5-methoxy-2-nitrobenzoic acid was obtained as an analytically pure orange solid (7.35 g, 28.5 mmol, 96%). **TLC**: *R*_f_ = 0.33 (20% MeOH/DCM, HOAc) [UV] **^1^H NMR** (500 MHz, DMSO): δ [ppm] = 13.59 (s, 1H), 7.69 (s, 1H), 7.47 – 7.45 (m, 2H), 7.43 – 7.40 (m, 2H), 7.38 – 7.35 (m, 2H), 7.31 (s, 1H), 5.24 (s, 2H), 3.91 (s, 3H). **^13^C NMR** (126 MHz, DMSO): δ [ppm] = 166.0, 152.0, 149.0, 141.1, 135.8, 128.5, 128.2, 128.1, 121.4, 111.4, 108.5, 70.5, 56.4. **HRMS** (ESI) *m/z*: (C_15_H_14_NO_6_^+^ [M-H]^+^) calc.: 304.0816, found: 304.0815.

**2-amino-4-(benzyloxy)-5-methoxybenzoic acid (8)**

4-(benzyloxy)-5-methoxy-2-nitrobenzoic acid (5.68 g, 18.8 mmol, 1.0 eq.) was dissolved in 60 mL THF and 50 mL H_2_O. Na_2_S_2_O_4_ (16.3 g, 93.8 mmol, 5.0 eq.) was added. The reaction mixture was stirred under reflux overnight. The phases formed were separated and the aqueous phase was extracted with EtOAc. The combined organic layers were concentrated under reduced pressure. The crude product was loaded onto C18 silica and purified by reversed-phase flash chromatography. The title compound was obtained as golden needles (2.47 g, 9.01 mmol, 48%). **TLC**: *R*_f_ = 0.22 (5% MeOH/DCM) [UV] **^1^H NMR** (500 MHz, DMSO): δ [ppm] = 7.46 – 7.42 (m, 1H), 7.42 – 7.39 (m, 1H), 7.36 – 7.33 (m, 1H), 7.17 (s, 1H), 6.44 (s, 1H), 5.05 (s, 2H), 3.65 (s, 3H). **^13^C NMR** (126 MHz, DMSO): δ [ppm] = 169.0, 153.6, 148.1, 139.3, 136.5, 128.4, 128.0, 127.9, 113.7, 100.9, 100.4, 69.4, 56.1. **HRMS** (ESI) *m/z*: (C_15_H_16_NO_4_^+^ [M+H]^+^) calc.: 274.1074, found: 274.1072.

**4-(benzyloxy)-2-((*tert*-butoxycarbonyl)amino)-5-methoxybenzoic acid (9)**

2-amino-4-(benzyloxy)-5-methoxybenzoic acid (2.96 g, 10.8 mmol, 1.0 eq.) was dissolved in THF (60 mL). NEt_3_ (6 mL) was added. Boc_2_O (2.79 mL, 2.83 g, 1.2 eq.) was added. The mixture was stirred overnight at room temperature. The solvent was removed under reduced pressure. The residue was dissolved in EtOAc and washed successively with HCl (0.1 m, 10 × 10 mL) and brine (2 × 10 mL). The organic phase was dried over Na_2_SO_4_ and the solvent was removed under reduced pressure. The oily residue was filtered through a cake of sand using PE as a solvent. The solid was further washed with DCM. The mother solution was concentrated under reduced pressure. The procedure was repeated three times, yielding the title compound as a beige solid (2.69 g, 7.21 mmol, 67%). **TLC**: *R*_f_ = 0.79 (PE:EtOAc, HOAc 5:1) [UV] **^1^H NMR** (500 MHz, DMSO): δ [ppm] = 13.34 (s, 1H), 10.57 (s, 1H), 8.14 (s, 1H), 7.72 – 7.28 (m, 5H), 5.12 (d, *J* = 8.6 Hz, 2H), 3.74 (s, 3H), 1.48 (s, 9H). **^13^C NMR** (126 MHz, DMSO): δ [ppm] = 169.3, 152.6, 152.1, 142.9, 137.3, 136.1, 128.4, 128.3, 128.2, 113.2, 106.4, 102.5, 80.1, 69.9, 55.6, 28.0. **HRMS** (ESI) *m/z*: (C_20_H_24_NO_6_^+^ [M+H]^+^) calc.: 374.1598, found: 374.1596.

**methyl(*S*)-1-(4-(benzyloxy)-2-((*tert*-butoxycarbonyl)amino)-5-methoxybenzoyl)-4-methylenepyrrolidine-2-carboxylate (10)**

To a solution of 4-(benzyloxy)-2-((*tert*-butoxycarbonyl)amino)-5-methoxybenzoic acid (2.20 g, 5.88 mmol, 1.3 eq.) in dry DMF (28 mL) and dry ACN (28 mL) HATU (2.23 g, 5.88 mmol, 1.3 eq.), HOBt (101 mg, 0.75 mmol, 0.2 eq.) and DIPEA (1.50 mL, 1.14 g, 8.85 mmol, 2.0 eq.) were added. After stirring for 30 min a solution of (*S*)-2-(methoxycarbonyl)-4-methylenepyrrolidiniumchloride (783 mg, 4.42 mmol, 1.0 eq.) in DMF (28 mL), Acetonitrile (28 mL) and DIPEA (1.50 mL, 1.14 g, 8.85 mmol, 2.0 eq.) was added. The mixture was stirred for 5 days at 35 °C. The amber-colored solution was diluted with EtOAc, washed with 0.1 m HCl and sat. NaCl aq.. The organic phase was dried over Na_2_SO_4_, and concentrated under reduced pressure. The crude product was purified by column chromatography (silica, PE:EtOAc, 10% 🡪 100% EtOAc) to obtain the product as a light yellow foam (2.12 g, 4.27 mmol, 96%). **TLC**: *R*_f_ = 0.55 (PE:EE 1:1) [UV] **^1^H NMR** (700 MHz, DMSO)^*^: δ [ppm] = 8.69/8.57 (s, 1H, CH_arom_), 7.50 – 7.47 (m, 2H, CH_arom_ (OBn)), 7.42 – 7.40 (m, 2H, CH_arom_ (OBn)) , 7.36 – 7.34 (m, 1H, CH_arom_ (OBn)), 6.91/6.71 (s, 1H, CH_arom_), 5.08 – 4.98 (m, 4H, CH_2_ (OBn), CH_2exo_), 4.71 – 4.59 (m, 1H, C*H*COOMe), 4.33 – 3.97 (m, 2H, C*H_2_*N), 3.78/3.68 (s, 3H, CH_3_), 3.68/3.59 (s, 3H, CH_3_), 3.05 – 2.60 (m, 1H, CC*H*_2_CHN), 2.64 – 2.50 (m, 1H, CC*H*_2_CHN), 1.43 (s, 9H). **^13^C NMR** (176 MHz, DMSO)^*^: δ [ppm] = 172.5, 171.9, 170.3, 168.1, 168.0, 153.3, 152.8, 149.0, 148.6, 145.0, 144.5, 143.1, 142.6, 136.6, 130.0, 129.1, 128.4, 128.0, 121.0, 118.3, 110.6, 110.0, 109.2, 108.0, 107.4, 79.3, 79.2, 70.0, 61.2, 58.3, 56.0, 55.6, 52.9, 52.3, 52.1, 49.9, 36.9, 34.7, 28.0. *Splitting of signals due to a 2:3 mixture of rotamers. **HRMS** (ESI) *m/z*: (C_27_H_33_N_2_O_7_^+^ [M+H]^+^) calc.: 497.2282, found: 497.2283.

***tert*-butyl(*S*)-(5-(benzyloxy)-2-(2-formyl-4-methylenepyrrolidine-1-carbonyl)-4-methoxyphenyl)carbamate (11)**

1.60 mL DIBAL (1 m in toluene, 1.60 mmol, 1.3 eq.) were added dropwise over a period of 10 min to a solution of methyl(S)-1-(4-(benzyloxy)-2-((*tert*-butoxycarbonyl)amino)-5-methoxybenzoyl)-4-methylenepyrrolidine-2-carboxylate (610 mg, 1.23 mmol, 1.0 eq.) in Et_2_O at -78 °C. After stirring for 2 h at the same temperature, MeOH (6 mL) and H_2_O (6 mL) were added dropwise. The mixture was allowed to warm up to room temperatureovernight. The formed layers were separated. Citric acid was added to the aqueous layer followed by extraction with EtOAc. The combined organic layers were dried over Na_2_SO_4_ and the solvent was removed under reduced pressure. The aldehyde was precipitated by dissolving in a small amount of EtOAc followed by the addition of PE. Alternatively, the product can be purified by column chromatography (silica, PE:EtOAc 2:1 🡪 0:1). The desired compound was obtained as a colorless solid (555 g, 1.19 mmol, 97%). **TLC**: *R*_f_ = 0.35 (PE:EtOAc 1:1) [UV] **^1^H NMR** (500 MHz, DMSO): δ [ppm] = 7.46 (d, *J* = 7.4 Hz, 2H, CH_arom_ (OBn)), 7.41 (t, *J* = 7.4 Hz, 2H, CH_arom_ (OBn)), 7.35 (t, *J* = 7.2 Hz, 1H, CH_arom_ (OBn)), 7.07 (s, 1H, CH_arom_), 6.77 (s, 1H, CH_arom_), 6.53 (s, 1H, OH), 5.33 (dd, *J* = 9.3, 5.9 Hz, 1H, CHC*H*OHN), 5.18 (d, *J* = 11.9 Hz, 1H, CH_2_ (OBn)), 5.13 (d, *J* = 11.9 Hz, 1H, CH_2exo_), 5.06 (d, *J* = 11.9 Hz, 1H, CH_2_ (OBn)), 4.09 (d, *J* = 15.8 Hz, 1H, CH*_2_*N), 3.97 (d, *J* = 15.8 Hz, 1H, CH*_2_*N), 3.82 (s, 3H, OCH_3_), 3.41 (t, *J* = 9.1 Hz, 1H, CH_2_C*H*N), 2.87 (dd, *J* = 15.6, 9.2 Hz, 1H, CC*H*_2_CHN), 2.52 (s, 1H, CC*H*_2_CHN), 1.20 (s, 9H, Boc). **^13^C NMR** (126 MHz, DMSO): δ [ppm] = 165.9, 153.7, 149.2, 147.9, 142.9, 136.6, 129.3, 128.5, 128.0, 127.5, 125.6, 114.8, 110.1, 109.0, 84.6, 80.1, 70.1, 60.2, 55.6, 50.2, 40.0, 39.9, 39.8, 39.7, 39.6, 39.6, 39.5, 39.3, 39.1, 38.0, 34.7, 27.8. **HRMS** (ESI) *m/z*: (C_26_H_31_N_2_O_6_^+^ [M+H]^+^) calc.: 467.2177; found: 467.2177.

**(*S*)-8-hydroxy-7-methoxy-2-methylene-1,2,3,11a-tetrahydro-5H-benzo[e]pyrrolo[1,2-a][1,4]diazepin-5-one (13b)**

*tert*-butyl(S)-(5-(benzyloxy)-2-(2-formyl-4-methylenepyrrolidine-1-carbonyl)-4-methoxyphenyl)carbamate (100 mg, 0.21 mmol, 1.0 eq.) was dissolved in DCM and cooled to 0 °C. Methanesulfonic acid (0.7 mL) was added dropwise. After stirring the mixture for 1.5 h at 0 °C, sat. NaHCO_3_ and AcOH were added and the pH was adjusted to pH = 4. The resulting phases were separated and the aqueous phase was thoroughly extracted with DCM. The combined organic layers were dried over Na_2_SO_4_ and directly purified, without a concentration step, by column chromatography (silica, DCM 🡪 5% *^t^*BuOH/DCM 🡪 7% *^t^*BuOH/DCM 🡪 10% *^i^*PrOH/DCM 🡪 22% *^i^*PrOH/DCM 🡪100% *^i^*PrOH). The product could be obtained as a colorless to light yellow solid (44.0 mg, 0.17 mmol, 81%). **TLC**: *R*_f_ = 0.48(MeOH/DCM 10%) [UV] **^1^H NMR** (500 MHz, DMSO)^*^: δ [ppm] = 7.72 (d, *J* = 4.4 Hz, 1H, CHN), 7.31 (s, 1H, CH_arom_), 7.17 (s, 1H, CH_arom_), 7.02 (s, 1H, CH_arom_), 6.68 (s, 1H, CH_arom_), 6.37 (s, 1H, CH_arom_), 6.17 (s, 1H, CH_arom_), 5.13 (d, *J* = 6.6 Hz, 2H, CH_2exo_), 5.08 (d, *J* = 17.6 Hz, 2H, CH_2exo_), 4.94 (d, *J* = 14.9 Hz, 2H, CH_2exo_), 4.83 (t, *J* = 6.1 Hz, 1H, C*H*OHN), 4.48 (dd, *J* = 8.7, 4.9 Hz, 1H, C*H*OHN), 4.26 – 3.94 (m, 6H, CH_2_N), 3.85 (quin, *J* = 4.2 Hz, 1H, CH_2_C*H*N), 3.82 (s, 3H, CH_3_), 3.81 – 3.77 (m, 1H, CH_2_C*H*N), 3.70 (s, 3H, CH_3_), 3.66 (s, 3H, CH_3_), 3.51 (td, *J* = 9.1, 2.1 Hz, 1H, CH_2_C*H*N), 3.08 – 2.52 (m, 6H, CC*H*_2_CHN). **^13^C NMR** (126 MHz, DMSO)*: δ [ppm] = 166.9, 165.9, 164.1, 163.8, 150.5, 149.9, 149.5, 146.2, 144.4, 143.4, 142.8, 142.0, 140.8, 140.1, 140.1, 139.0, 118.2, 116.2, 114.7, 112.9, 112.8, 111.6, 109.6, 108.5, 108.4, 108.2, 105.7, 104.1, 85.9, 81.5, 59.6, 59.1, 56.0, 55.9, 55.7, 53.5, 53.1, 51.1, 51.0, 35.6, 35.1, 34.7. **HRMS** (ESI) *m/z*: (C_14_H_15_N_2_O_3_^+^ [M+H]^+^) calc.: 259.1077, found: 259.1078.

* Due to residual water in the DMSO-*d_6_* the NMR corresponds to a mixture of the imine form and the two hemiaminal forms (diastereomers).

**(*S*)-8-(2-bromoethoxy)-7-methoxy-2-methylene-1,2,3,11a-tetrahydro-5H-benzo[e]pyrrolo[1,2-a][1,4]diazepin-5-one (14)**

(*S*)-8-hydroxy-7-methoxy-2-methylene-1,2,3,11a-tetrahydro-5H-benzo[e]pyrrolo[1,2-a][1,4]diazepin-5-one (30.0 mg, 116 µmol, 1.0 eq.) and K_2_CO_3_ (1.60 g, 11.6 mmol, 100 eq.) were dissolved in 2 mL dry DMF. 0.10 mL 1,2-dibromoethane (217 mg, 1.16 mmol, 10 eq.) were added. The mixture was stirred at RT overnight. After the addition of H_2_O and DCM, the aqueous phase was thoroughly extracted with DCM. The organic layers were dried over Na_2_SO_4_, filtered and evaporated under reduced pressure. The crude product was purified by column chromatography (silica, 100% EtOAc) yielding the title compound as a yellow film (32.8 mg, 89.8 µmol, 80%). **TLC**: *R*_f_ = 0.30 (100% EtOAc) [UV] **^1^H NMR** (500 MHz, CDCl_3_): δ [ppm] = 7.68 (d, *J* = 4.4 Hz, 1H), 7.51 (s, 1H), 6.81 (s, 1H), 5.17 (d, *J* = 14.1 Hz, 2H), 4.37 (ddt, *J* = 32.0, 10.7, 6.7 Hz, 2H), 4.27 (s, 2H), 3.94 (s, 3H), 3.89 – 3.84 (m, 1H), 3.68 (t, *J* = 6.7 Hz, 2H), 3.16 – 2.90 (m, 3H). **^13^C NMR** (126 MHz, CDCl_3_): δ [ppm] = 164.7, 162.9, 162.7, 150.0, 148.1, 141.6, 140.6, 120.9, 112.1, 111.4, 109.6, 77.4, 77.2, 76.9, 68.8, 56.4, 53.9, 51.5, 35.6, 28.3. **HRMS** (ESI) *m/z*: (C_16_H_18_BrN_2_O_3_^+^ [M+H]^+^) calc.: 365.0495, found: 365.0497.

**(*S*)-8-(2-azidoethoxy)-7-methoxy-2-methylene-1,2,3,11a-tetrahydro-5H-benzo[e]pyrrolo[1,2-a][1,4]diazepin-5-one (MbA)**

(*S*)-8-(2-bromoethoxy)-7-methoxy-2-methylene-1,2,3,11a-tetrahydro-5H-benzo[e]pyrrolo[1,2-a][1,4]diazepin-5-one (36.0 mg, 98.6 µmol, 1.0 eq.) was dissolved in DMF (0.66 mL). NaN_3_ (64.1 mg, 986 µmol, 10.0 eq.) was added and the mixture was stirred over night at 50 °C. After DCM and water were added, the aqueous phase was thoroughly extracted with DCM. The organic phase was dried over Na_2_SO_4_, filtered and evaporated under reduced pressure. The crude product was purified by column chromatography (silica, MeOH/DCM, 1% 🡪 5%). The pure product was obtained as a colorless solid (25.7 mg, 78.6 µmol, 80%). **TLC**: *R*_f_ = 0.31(EtOAc 100%) [UV] **^1^H NMR** (700 MHz, DMSO)^*^: δ [ppm] = 7.78 (d, *J* = 4.4 Hz, 1H, CHN), 7.22 (s, 1H, CH_arom_), 7.07 (s, 1H, CH_arom_), 6.88 (s, 1H, CH_arom_), 6.84 (d, *J* = 6.1 Hz, 1H, OH), 6.58 (s, 1H, CH_arom_), 6.36 (s, 1H, CH_arom_), 5.99 (d, *J* = 6.9 Hz, 1H, OH), 5.50 (d, *J* = 5.7 Hz, 2H, NH), 5.14 (d, *J* = 9.1 Hz, 2H, CH_2exo_) 5.09 (d, *J* = 25.0 Hz, 2H, CH_2exo_), 4.95 (d, *J* = 21.5 Hz, 2H, CH_2exo_), 4.87 (t, *J* = 6.2 Hz, 1H, C*H*OHN), 4.53 (t, *J* = 7.9 Hz, 1H, C*H*OHN), 4.25 – 4.14 (m, 4H, CH_2_N and OC*H*_2_CH_2_), 4.13 – 4.08 (m, 2H, OC*H*_2_CH_2_), 4.06 (t, *J* = 4.9 Hz, 2H, OC*H*_2_CH_2_), 3.97 (d, *J* = 15.7 Hz, 1H, CH_2_N), 3.87 (quin, *J* = 6.1 Hz, 1H, CH_2_C*H*N), 3.83 – 3.80 (m, 1H, CH_2_C*H*N), 3.82 – 3.81 (m, 2H, CH_2_C*H*_2_N_3_), 3.70 (s, 3H, CH_3_), 3.68 (m, 2H, CH_2_C*H*_2_N_3_), 3.68 (s, 3H, CH_3_), 3.66 (s, 3H, CH_3_), 3.54 (td, *J* = 9.0, 1.9 Hz, 1H, CH_2_C*H*N), 3.06 – 3.04 (m, 2H, CC*H*_2_CHN), 2.94 – 2.86 (m, 2H, CC*H*_2_CHN), 2.66 (d, *J* = 16.3 Hz, 1H CC*H*_2_CHN), 2.55 (d, *J* = 15.8 Hz, 1H, CC*H*_2_CHN). **^13^C NMR** (176 MHz, DMSO)^*^: δ [ppm] = 166.6, 165.7, 164.5, 163.5, 151.1, 150.4, 149.9, 147.1, 144.2, 143.2, 142.9, 142.7, 140.8, 140.5, 139.9, 138.9, 119.8, 117.2, 114.8, 112.9, 111.4, 110.5, 108.5, 108.4, 106.3, 105.9, 102.2, 85.8, 81.4, 67.5, 67.3, 67.0, 59.5, 59.1, 56.0, 56.0, 55.8, 53.5, 53.2, 51.2, 51.0, 49.6, 35.6, 35.1, 34.7. **HRMS** (ESI) *m/z*: (C_16_H_18_N_5_O_3_^+^ [M+H]^+^) calc.: 328.1404, found: 328.1405.

* Due to residual water in the DMSO-*d_6_* the NMR corresponds to a mixture of the imine form and the two hemiaminal forms (diastereomers). The two hemiaminals are the predominant forms in the mixture, the imine is only found in minor quantities (ca. 7-10%).

**(*S*)-7-methoxy-2-methylene-8-(prop-2-yn-1-yloxy)-1,2,3,11a-tetrahydro-5H-benzo[e]pyrrolo[1,2-a][1,4]diazepin-5-one (M1)**

(*S*)-8-hydroxy-7-methoxy-2-methylene-1,2,3,11a-tetrahydro-5H-benzo[e]pyrrolo[1,2-a][1,4]diazepin-5-one (20.0 mg, 78.0 µmol, 1.0 eq.) and K_2_CO_3_ (1.08 g, 7.80 mmol, 100 eq.) were dissolved in dry DMF (1.00 mL). 100 µL Propargyl bromide (80w% in toluene, 107 mg, 0.78 mmol, 10 eq.) was added. The reaction was stirred at RT overnight. H_2_O was added to the reaction and the mixture was extracted thoroughly with DCM. The combined organic phase was washed with water and brine. The solvent was removed under reduced pressure. The crude product was purified by column chromatography (silica, PE:EtOAc 1:1 🡪 1:3) yielding the title compound as a light yellow highly viscous oil (15.0 mg, 50.6 µmol, 65%). **TLC**: *R*_f_ = 0.35 (PE:EtOAc 1:4) [UV] **^1^H NMR** (700 MHz, DMSO)*: δ [ppm] = 7.78 (d, *J* = 4.5 Hz, 1H, CHN), 7.35 (s, 1H, CH_arom_), 7.22 (s, 1H, CH_arom_), 7.07 (s, 1H, CH_arom_), 6.93 (s, 1H, CH_arom_), 6.91 (d, *J* = 6.2 Hz, 1H, OH), 6.61 (s, 1H, CH_arom_), 6.39 (s, 1H, CH_arom_), 5.53 (s, 1H, NH), 5.50 (d, *J* = 4.8 Hz, 1H, NH), 5.14 (d, *J* = 9.2 Hz, 2H, CH_2exo_), 5.08 (d, *J* = 24.8 Hz, 2H, CH_2exo_), 4.94 (dt, *J* = 21.9, 1.7 Hz, 2H, CH_2exo_), 4.90 (d, *J* = 2.3 Hz, 2H, OC*H*_2_CCH), 4.75 – 4.73 (m, 2H, OC*H*_2_CCH), 4.72 (d, *J* = 2.4 Hz, 2H, OC*H*_2_CCH), 4.24 – 4.11 (m, 5H, CH_2_N), 3.97 (dd, *J* = 15.7, 1.2 Hz, 1H, CH_2_N), 3.88 (quin, *J* = 4.3 Hz, 1H, CH_2_C*H*N), 3.83 (s, 3H, CH_3_), 3.81 (dd, *J* = 9.2, 4.6 Hz, 1H, CH_2_C*H*N), 3.70 (s, 3H, CH_3_), 3.66 (s, 3H, CH_3_), 3.63 (t, *J* = 2.4 Hz, 1H, CH), 3.61 (t, *J* = 2.4 Hz, 1H, CH), 3.60 (t, *J* = 2.4 Hz, 1H, CH), 3.55 (td, *J* = 9.1, 2.2 Hz, 1H, CH_2_C*H*N), 3.10 – 3.00 (m, 2H, CC*H*_2_CHN), 2.93 – 2.87 (m, 2H, CC*H*_2_CHN), 2.66 (dd, *J* = 16.4, 2.2 Hz, 1H, CC*H*_2_CHN), 2.55 (d, *J* = 15.2 Hz, 1H, CC*H*_2_CHN). **^13^C NMR** (176 MHz, DMSO)*: δ [ppm] = 166.5, 165.6, 164.6, 163.5, 149.9, 149.5, 148.8, 147.1, 144.2, 143.2, 142.9, 142.7, 140.8, 140.3, 139.6, 138.6, 120.1, 117.4, 114.5, 112.6, 111.3, 110.9, 110.6, 108.5, 108.3, 106.6, 105.9, 102.6, 85.7, 81.1, 78.9, 78.9, 78.8, 78.7, 78.5, 78.5, 59.4, 59.1, 56.0, 55.9, 55.8, 55.7, 55.6, 53.5, 53.2, 51.1, 51.0, 35.7, 35.1, 34.7. **HRMS** (ESI) *m/z*: (C_17_H_17_N_2_O_3_^+^ [M+H]^+^) calc.: 297.1234, found: 297.1235

* Due to residual water in the DMSO-*d_6_* the NMR corresponds to a mixture of the imine form and the two hemiaminal forms (diastereomers).

**(*S*)-8-(but-3-yn-1-yloxy)-7-methoxy-2-methylene-1,2,3,11a-tetrahydro-5H-benzo[e]pyrrolo[1,2-a][1,4]diazepin-5-one (M2)**

(*S*)-8-hydroxy-7-methoxy-2-methylene-1,2,3,11a-tetrahydro-5H-benzo[e]pyrrolo[1,2-a][1,4]diazepin-5-one (20.0 mg, 78.0 µmol, 1.0 eq.) and K_2_CO_3_ (1.08 g, 7.80 mmol, 100 eq.) were dissolved in dry DMF (1.00 mL). 4-bromobut-1-yne (439 µL, 4.68 mmol, 60 eq.) was added in portions throughout the reaction. The mixture was stirred at room temperature over the weekend. H_2_O was added to the reaction and the mixture was extracted thoroughly with DCM. The combined organic phases were washed with water and brine. The solvent was removed under reduced pressure. The crude product was purified by column chromatography (silica, PE:EtOAc 1:4) yielding the title compound as a light yellow highly viscous oil (12.5 mg, 40.0 µmol, 51%) **TLC**: *R*_f_ = 0.39 (PE:EtOAc 1:4) [UV] **^1^H NMR** (700 MHz, DMSO)*: δ [ppm] = 7.77 (d, *J* = 4.4 Hz, 1H, CHN), 7.34 (s, 1H, CH_arom_), 7.20 (s, 1H, CH_arom_), 7.05 (s, 1H, CH_arom_), 6.84 (s, 1H, CH_arom_), 6.82 (d, *J* = 6.2 Hz, 1H, OH), 6.58 (s, 1H, CH_arom_), 6.35 (s, 1H, CH_arom_), 6.07 – 5.94 (m, 1H, OH), 5.52 – 5.50 (m, 2H, NH), 5.14 (d, *J* = 9.5 Hz, 2H, CH_2exo_), 5.08 (d, *J* = 24.7 Hz, 2H, CH_2exo_), 4.95 (d, *J* = 21.1 Hz, 2H, CH_2exo_), 4.87 (t, *J* = 5.2 Hz, 1H, C*H*OHN), 4.53 – 4.52 (m, 1H, C*H*OHN) 4.27 – 3.95 (m, 12H, CH_2_N (3x), OC*H*_2_CH_2_ (3x)), 3.86 (quin, *J* = 4.2 Hz, 1H, CH_2_C*H*N), 3.83 (s, 3H, OCH_3_), 3.82 – 3.80 (m, 1H, CH_2_C*H*N), 3.70 (s, 3H, OCH_3_), 3.66 (s, 3H, OCH_3_), 3.53 (dt, *J* = 9.1, 1.9 Hz, 1H, CH_2_C*H*N), 3.08 – 3.00 (m, 1H, CC*H*_2_CHN), 2.95 – 2.86 (m, 4H, CC*H*_2_CHN), 2.69 – 2.63 (m, 6H, CC*H*_2_CHN), 2.54 (d, *J* = 15.8 Hz, 1H, CC*H*_2_CHN). **^13^C NMR** (176 MHz, DMSO)*: δ [ppm] = 166.6, 165.7, 164.5, 151.1, 150.5, 144.2, 143.2, 142.7, 140.6, 139.9, 138.9, 116.8, 114.6, 112.7, 111.4, 110.2, 108.5, 108.4, 105.9, 101.9, 85.8, 81.3, 81.2, 72.6, 66.3, 66.1, 59.5, 59.1, 55.9, 55.8, 55.7, 53.5, 53.2, 51.1, 51.0, 39.5, 35.6, 35.1, 18.8. **HRMS** (ESI) *m/z*: (C_18_H_19_O_3_^+^ [M+H]^+^) calc.: 311.1390, found: 311.1392

* Due to residual water in the DMSO-*d_6_* the NMR corresponds to a mixture of the imine form and the two hemiaminal forms (diastereomers). The imine form is only percent in minor quantities (1:1:0.22).

**8,8-dibromobicyclo[5.1.0]octane (16)**

A stirred suspension of *cis*-cycloheptene (3.00 g, 31.3 mmol, 1.0 eq.) and potassium *tert*-butoxide (4.02 g, 35.9 mmol, 1.15 eq) in anhydrous pentane (100 ml) was cooled to 0 °C. A solution of bromoform (2.72 mL, 7.87 g, 31.3 mmol, 1.0 eq.) in anhydrous pentane (120 mL) was added dropwise over a period of 6 h at 0 °C. After complete addition, the resulting brown mixture was warmed to room temperature and stirred overnight. After addition of water (200 mL), the mixture was acidified with HCl (6 m) and the aqueous layer was extracted with Et_2_O. The combined organic layers were dried over Na_2_SO_4_. The product (4.50 g, 16.8 mmol, 53%) was obtained as a brown oil and used without further purification. **TLC**: *R*_f_ = 0.65(PE) [UV] **^1^H NMR** (500 MHz, CDCl_3_): δ [ppm] = 2.27 – 2.23 (m, 2H), 1.93 – 1.86 (m, 1H), 1.85 – 1.80 (m, 2H), 1.74 – 1.67 (m, 2H), 1.32 – 1.28 (m, 2H), 1.22 – 1.15 (m, 3H). **^13^C NMR** (126 MHz, CDCl_3_): δ [ppm] = 40.8, 34.8, 32.4, 29.0, 28.1.

**(*E*)-2-((2-bromocyclooct-2-en-1-yl)oxy)ethan-1-ol (17)**

8,8-dibromobicyclo[5.1.0]octane (4.50 g, 16.8 mmol, 1.0 eq.) and ethane-1,2-diol (18.7 mL, 20.8 g, 63.1 mmol, 20.0 eq.) were dissolved in acetone (30 mL). Anhydrous AgClO_4_ (10.4 g, 50.4 mmol, 3.0 eq.) was added in small portions under the exclusion of light and stirred at room temperaturefor 4 h. After the addition of EtOAc (100 mL) and filtration, 1 m HCl (100 mL) was added and the aqueous layer was extracted with EtOAc. The combined organic layers were washed with 1 m HCl, H_2_O, sat. NaCl solution and dried over Na_2_SO_4_. The solvent was evaporated under reduced pressure and the (crude) product (3.84 g, 15.4 mmol, 92%) was obtained as a brown oil and used without further purification, due to light sensitivity. **TLC**: *R*_f_ = 0.36(PE:EtOAc 2:1) [UV, KMnO_4_]

**2-(cyclooct-2-yn-1-yloxy)ethan-1-ol (18)**

DBU (23 mL, 23.4 g, 154 mmol, 10.0 eq.) was added in portions to a solution of (*E*)-2-((2-bromocyclooct-2-en-1-yl)oxy)ethan-1-ol (3.84 g, 15.4 mmol, 1.0 eq.) in DMSO (30 mL) at 60 °C. After stirring overnight, the solution was allowed to cool to room temperatureand EtOAc and H_2_O were added. After acidification to pH = 1 with conc. HCl, the aqueous phase was extracted with EtOAc. The combined organic layers were washed with 1 m HCl, sat. NaCl, dried over Na_2_SO_4_ and concentrated under reduced pressure. The crude product was purified by flash column chromatography (silica, PE:EtOAc 1:0 🡪 2:3) yielding 2-(cyclooct-2-yn-1-yloxy)ethan-1-ol as a light yellow oil (1.03 g, 6.12 mmol, 40%). **TLC**: *R*_f_ = 0.39(PE:EtOAc 2:1) [UV, Iodine] **^1^H NMR** (500 MHz, CDCl_3_): δ [ppm] = 4.23 – 4.20 (m, 1H), 3.78 – 3.72 (m, 2H), 3.70 – 3.65 (m, 1H), 3.48 – 3.44 (m, 1H), 2.28 – 2.10 (m, 3H), 2.01 – 1.90 (m, 2H), 1.86 – 1.78 (m, 2H), 1.71 – 1.60 (m, 2H), 1.48 – 1.42 (m, 1H). **^13^C NMR** (126 MHz, CDCl_3_): δ [ppm] = 100.6, 92.7, 62.0, 42.4, 34.4, 29.9, 26.5, 20.8. **HRMS** (ESI) *m/z*: (C_10_H_17_O_2_^+^ [M+H]^+^) calc.: 169.1223, found: 169.1223

**2-(cyclooct-2-yn-1-yloxy)ethyl 4-methylbenzenesulfonate (19)**

2-(cyclooct-2-yn-1-yloxy)ethan-1-ol was dissolved in 16 mL ACN. Pyridine (2.58 mL, 2.53 g, 32.0 mmol, 11.1 eq.) and TosCl (809 mg, 4.26 mmol, 1.5 eq.) were added in portions. The mixture was stirred for 8 h at room temperature. The reaction was monitored by TLC. The solution was filtered. PE was added to the solution and the mixture was washed with NaCl aq. and NH_4_Cl aq.. The organic phase was separated and the solvent was removed under reduced pressure. The crude product was purified by column chromatography (silica, PE:EtOAc 2:3). The product was obtained as a colorless oil (648 mg, 2.01 mmol, 70%). **TLC**: *R*_f_ = 0.73 (PE:EtOAc 2:1) [Iodine] *R*_f_ = 0.28 (PE:EtOAc 4:1) [K_2_MnO_4_] **^1^H NMR** (500 MHz, CD_2_Cl_2_): δ [ppm] = 7.37 (d, *J* = 8.05 Hz, 1H), 7.78 (d, *J* = 8.30 Hz, 1H), 4.13 –4.11 (m, 1H), 3.71 –3.67 (m, 1H), 3.50 –3.46 (m, 1H), 2.45 (s, 3H), 2.25 –2.19 (m, 1H), 2.17 –2.10 (m, 1H), 2.06 –2.00 (m, 1H), 1.93 – 1.71 (m, 5H), 1.67 – 1.55 (m, 2H). **^13^C NMR** (126 MHz, CD_2_Cl_2_): δ [ppm] = 145.1, 133.0, 129.9, 127.9, 100.6, 92.0, 72.7, 69.5, 66.4, 42.2, 34.3, 29.7, 26.3, 21.4, 20.6. **HRMS** (ESI) *m/z*: (C_17_H_26_NO_4_S^+^ [M+H]^+^) calc.: 340.1577, found: 340.1578

**(11a*S*)-8-(2-(cyclooct-2-yn-1-yloxy)ethoxy)-7-methoxy-2-methylene-1,2,3,11a-tetrahydro-5H-benzo[e]pyrrolo[1,2-a][1,4]diazepin-5-one (M3)**

A flask equipped with K_2_CO_3_ (1.00 g, 7.24 mmol, 75 eq.) was dried under vacuum using a heat gun. After cooling to room temperature, (*S*)-8-hydroxy-7-methoxy-2-methylene-1,2,3,11a-tetrahydro-5H-benzo[e]pyrrolo[1,2-a][1,4]diazepin-5-one (25.0 mg, 96.9 µmol, 1.0 eq.) and 2-(cyclooct-2-yn-1-yloxy)ethyl 4-methylbenzenesulfonate (94.0 mg, 291 µmol, 3.0 eq.) were added. Upon addition of DMF (9.7 mL) the reaction mixture was stirred at RT overnight. The reaction was monitored by LC‒MS. H_2_O and DMF were added. The aqueous phase was thoroughly extracted with DCM. The combined organic layers were dried over Na_2_SO_4_ and concentrated under vacuum. The crude product was purified by column chromatography (1. column: DCM:MeOH 5%, 2. column: DCM:MeOH 0.5%) to yield the title compound (26.7 mg, 65.4 µmol, 68%). **TLC**: *R*_f_ = 0.40 (EtOAc 100%) [UV] **^1^H NMR** (700 MHz, DMSO)*: δ [ppm] = 7.77 (d, *J* = 4.4 Hz, 1H, CHN), 7.33 (s, 1H, CH_arom_), 7.19 (s, 1H, CH_arom_), 7.05 (s, 1H, CH_arom_), 6.85 (s, 1H, CH_arom_), 6.83 (d, *J* = 5.8 Hz, 1H, OH), 6.55 (s, 1H, CH_arom_), 6.33 (s, 1H, CH_arom_), 5.58 (d, *J* = 4.4 Hz, 1H, NH), 5.14 (d, *J* = 8.8 Hz, 2H, CH_2exo_), 5.08 (d, *J* = 24.3 Hz, 2H, CH_2exo_), 4.95 (d, *J* = 21.2 Hz, 2H, CH_2exo_), 4.87 (d, *J* = 6.2 Hz, 1H, CHC*H*OHNH), 4.53 (d, *J* = 9.0 Hz, 1H, CHC*H*OHNH), 4.30 (brs, 3H, CH_propargyl_), 4.24 – 3.94 (m, 12H, CH_2ethyl_, CH_2_N), 3.85 (quin, *J* = 4.2 Hz, 1H, CH_2_C*H*N), 3.82 (s, 3H, OCH_3_), 3.82 – 3.74 (m, 4H, CH_2ethyl_, CH_2_C*H*N), 3.70 (s, 3H, OCH_3_), 3.66 (s, 3H, OCH_3_), 3.64 – 3.60 (m, 3H, CH_2ethyl_), 3.52 (td, *J* = 9.0, 2.0 Hz, 1H, CH_2_C*H*N), 3.09 – 3.00 (m, 2H, CC*H*_2_CHN), 2.95 – 2.85 (m, 2H, CC*H*_2_CHN), 2.66 (dd, *J* = 16.3, 2.0 Hz, 1H, CC*H*_2_CHN), 2.54 (d, *J* = 15.6 Hz, 1H, CC*H*_2_CHN), 2.26 – 2.20 (m, 3H, CH_2octyne_), 2.14 (dtd, *J* = 16.7, 5.8, 2.2 Hz, 3H, CH_2octyne_), 2.09 – 2.03 (m, 3H, CH_2octyne_), 1.86 (dt, *J* = 13.4, 6.8 Hz, 6H, CH_2octyne_), 1.80 – 1.70 (m, 6H, CH_2octyne_), 1.66 – 1.59 (m, 3H, CH_2octyne_), 1.52 (dt, *J* = 27.2, 10.0 Hz, 3H, CH_2octyne_), 1.41 – 1.34 (m, 3H, CH_2octyne_). **^13^C NMR** (176 MHz, DMSO)*: δ [ppm] = 166.6, 165.7, 165.3, 164.3, 163.6, 151.4, 150.8, 150.3, 147.0, 144.2, 143.3, 142.7, 140.7, 140.6, 139.9, 139.0, 119.4, 116.6, 114.6, 112.7, 111.3, 110.3, 110.0, 108.5, 108.3, 105.8, 101.9, 100.0, 93.0, 85.8, 81.3, 71.9, 67.9, 67.9, 67.6, 67.3, 66.9, 59.5, 59.1, 55.8, 55.6, 53.5, 53.2, 51.1, 51.0, 41.9, 35.6, 35.1, 34.7, 33.9, 29.3, 25.9, 20.0. **HRMS** (ESI) *m/z*: (C_24_H_29_N_2_O_4_^+^ [M+H]^+^) calc.: 409.2122, found: 409.2121.

* Due to residual water in the DMSO-*d_6_* the NMR corresponds to a mixture of the imine form and the two hemiaminal forms (diastereomers).

**bicyclo[6.1.0]non-4-yn-9-ylmethyl (4-nitrophenyl) carbonate (21)**

To a solution of 10 mg (0.07 mmol, 1.0 eq.) of ((1*R*,8*S*,9*S*)- bicyclo[6.1.0]non-4-yn-9-yl)methanol in 1.66 mL dry DCM was added 13.4 μL (0.17 mmol, 2.5 eq.) pyridine and 16.8 mg (0.08 mmol, 1.25 eq.) 4-nitrophenyl chloroformate and the mixture was stirred for 20 min at room temperature, before it was quenched by addition of 20 mL saturated ammonium chloride solution. The mixture was extracted with DCM, the combined organic layers were dried over Na_2_SO_4_ and concentrated under reduced pressure. The crude product was purified by column chromatography (silica, PE:EtOAc 95:5 🡪 9:1). The product was obtained as a colorless solid (18.4 mg, 58.5 µmol, 88%). **TLC**: *R*_f_ = 0.50 (PE:EtOAc 9:1) [Iodine] **^1^H NMR** (500 MHz, CD_2_Cl_2_): δ [ppm] = 8.27 (d, *J* = 9.2 Hz, 2H), 7.40 (d, *J* = 9.2 Hz, 2H), 4.40 (d, *J* = 8.3 Hz, 2H), 2.33 – 2.28 (m, 4H), 2.24 – 2.20 (m, 2H), 1.65 – 1.57 (m, 2H), 1.54 – 1.48 (m, 1H), 1.07 – 1.03 (m, 1H). **^13^C NMR** (126 MHz, CD_2_Cl_2_): δ [ppm] = 155.8, 152.6, 145.4, 125.3, 122.0, 98.6, 68.1, 29.1, 21.3, 20.5, 17.3. **HRMS** (ESI) *m/z*: (C_17_H_18_NO_5_^+^ [M+H]^+^) calc.: 316.1179, found: 316.1173.

**bicyclo[6.1.0]non-4-yn-9-ylmethyl((*S*)-7-methoxy-2-methylene-5-oxo-2,3,5,11a-tetrahydro-1H-benzo[e]pyrrolo[1,2-a][1,4]diazepin-8-yl) carbonate (M4)**

A flask equipped with K_2_CO_3_ (1.00 g, 7.24 mmol, 75 eq.) was dried under vacuum using a heat gun. After cooling to rt, (*S*)-8-hydroxy-7-methoxy-2-methylene-1,2,3,11a-tetrahydro-5H-benzo[e]pyrrolo[1,2-a][1,4]diazepin-5-one (25 mg, 96.9 µmol, 1.0 eq.) and bicyclo[6.1.0]non-4-yn-9-ylmethyl (4-nitrophenyl) carbonate (40 mg, 125 µmol, 1.3 eq.) were added. Upon addition of DMF (9.70 mL) the reaction mixture, which immediately turned bright yellow, was stirred at RT over night. DCM was added and the mixture was filtered (pore 5 filter). The filtrate was concentrated under reduced pressure and purified by column chromatography (silica, PE:EtOAc 1:0 🡪1:3; alternatively 10% MeOH/DCM). The product was obtained as a yellow film (18.1 mg, 41.8 µmol, 43%). **TLC**: *R*_f_ = 0.34 (PE:EtOAc 1:2) **^1^H NMR** (700 MHz, DMSO)*: δ [ppm] = 7.82 (d, *J* = 4.5 Hz, 1H, CHN), 7.51 (s, 1H, CH_arom_), 7.20 (s, 1H, CH_arom_), 5.15 (d, *J* = 8.8 Hz, 2H, CH_2exo_), 4.36 (d, *J* = 8.3 Hz, 2H, OC*H*_2_CH), 4.15 (dd, *J* = 41.7, 15.8 Hz, 2H, CH_2_N), 3.95 (quin, *J* = 4.22 Hz, 1H, CH_2_C*H*N), 3.87 (s, 3H, CH_3_), 3.09 – 3.01 (m, 2H, CC*H*_2_CHN), 2.27 – 2.24 (m, *J* = 13.5 Hz, 2H, CH_2_C*H*_2_CC_octyne_), 2.17 – 2.14 (m, 4H, CH_2_C*H*_2_CC_octyne_, C*H*_2_CH_2_CC_octyne_), 1.63 – 1.52 (m, 2H, , C*H*_2_CH_2_CC_octyne_), 1.40 (q, *J* = 8.6 Hz, 1H, OCH_2_C*H*_cyclopropane_), 1.00 – 0.92 (m, 2H, C*H*CH_2_CH_2cyclopropane_). **^13^C NMR** (176 MHz, DMSO)*: δ [ppm] = 165.7, 163.0, 152.1, 148.9, 142.5, 141.6, 139.7, 125.7, 120.8, 112.6, 108.7, 98.9, 67.3, 56.2, 54.9, 53.5, 51.2, 49.8, 34.7, 28.5, 20.8, 19.9, 17.1. * Due to residual water in the DMSO-*d_6_* the NMR corresponds to a mixture of the imine form and the two hemiaminal forms (diastereomers). **HRMS** (ESI) *m/z*: (C_25_H_27_N_2_O_5_^+^ [M+H]^+^) calc.: 435.1914, found: 435.1914.

**(S)-7-methoxy-8-((1-(2-(((S)-7-methoxy-2-methylene-5-oxo-2,3,5,11a-tetrahydro-1H-benzo[e]pyrrolo[1,2-a][1,4]diazepin-8-yl)oxy)ethyl)-1H-1,2,3-triazol-4-yl)methoxy)-2-methylene-1,2,3,11a-tetrahydro-5H-benzo[e]pyrrolo[1,2-a][1,4]diazepin-5-one (D1)**

(*S*)-8-(2-azidoethoxy)-7-methoxy-2-methylene-1,2,3,11a-tetrahydro-5H-benzo[e]pyrrolo [1,2-a][1,4]diazepin-5-one (8.0 mg, 24.0 µmol, 1.0 eq.) and (*S*)-8-(but-3-yn-1-yloxy)-7-methoxy-2-methylene-1,2,3,11a-tetrahydro-5H-benzo[e]pyrrolo[1,2-a][1,4]diazepin-5-one (7.0 mg, 24.0 µmol, 1.0 eq.) were dissolved in DMF (0.4 mL) and stirred at room temperature.

480 µL of a sodium ascorbate solution (100 mm, 48.0 µmol, 2.0 eq.) and 120 µL of a CuSO_4_ solution (100 mm, 12.0 µmol, 0.5 eq.) were mixed, 120 µL of a THPTA solution (100 mm, 12.0 µmol, 0.5 eq.) was added to the solution. The mixture was added to the previously prepared solution. After 1 h EtOAc and H_2_O were added to the mixture. The phases were separated and the aqueous phase was extracted with DCM and EtOAc. The combined organic layers were dried over Na_2_SO_4_, filtered, and concentrated under reduced pressure. Purification by preparative TLC (4% MeOH/DCM) gave the title compound as a colorless solid (5.7 mg, 9.0 µmol, 38%). **TLC**: *R*_f_ = 0.36(MeOH/DCM, 1%) [UV] **^1^H NMR** (700 MHz, DMSO)*: δ [ppm] = 8.31 – 8.29 (m, 1H), 8.06 (s, 0.2H), 7.78 – 7.77 (m, 0.3H), 7.33 – 7.32 (m, 0.3 H), 7.29 – 7.25 (m, 1H), 7.22 – 7.20 (m, 1H), 7.08 (s, 0.2H), 7.05 (d, *J* = 1.7 Hz, 2H), 6.90 (t, *J* = 2.6 Hz, 0.2H), 6.80 (s, 0.5H), 6.63 (s, 0.5H), 6.54 (s, 0.3H), 6.40 (s, 0.3H), 6.13 (d, *J* = 9.3 Hz, 1H), 5.32 (t, *J* = 4.7 Hz, 0.2H), 5.27 (d, *J* = 12.1 Hz, 0.2H), 5.20 – 5.07 (m, 6H), 4.96 (dd, *J* = 17.7, 2.0 Hz, 2H), 4.82 (brs, 2H), 4.66 (s, 0.3H), 4.53 (t, 6.0 Hz, 1H), 4.48 – 4.43 (m, 1H), 4.40 – 4.24 (m, 2H), 4.26 (td, *J* = 9.2, 1.3 Hz, 1H), 4.19 – 4.11 (m, 4H), 3.97 (dd, *J* = 15.6, 2.9 Hz, 1H), 3.87 – 3.85 (m, 1H), 3.79 – 3.76 (m, 2H), 3.69 – 3.59 (m, 7H), 3.51 (s, 0.3H), 3.36 (d, *J* = 4.0 Hz, 3H), 3.21 (d, *J* = 4.8 Hz, 2H), 3.00 – 2.91 (m, 2H), 2.68 (d, *J* = 16.3 Hz, 1H), 2.46 (d, *J* = 15.9 Hz), 2.02 – 1.97 (m, 1H), 1.23 (brs, 6H). **^13^C NMR** (176 MHz, DMSO)*: δ [ppm] = 174.2, 166.8, 166.7, 165.1, 165.0, 164.7, 164.5, 164.4, 163.5, 163.4, 151.0, 151.0, 150.2, 150.1, 149.8, 149.6, 147.1, 147.0, 143.8, 143.7, 143.7, 143.2, 143.1, 142.7, 142.6, 142.4, 142.3, 140.8, 140.8, 140.5, 140.5, 139.3, 139.2, 138.1, 138.0, 133.5, 129.6, 129.6, 125.4, 125.3, 125.3, 120.3, 120.0, 119.8, 119.6, 115.4, 114.6, 112.9, 112.2, 111.6, 111.1, 110.9, 110.6, 110.2, 109.7, 108.5, 108.4, 107.8, 107.5, 106.0, 102.3, 102.0, 94.5, 88.4, 88.3, 69.8, 67.1, 67.0, 66.6, 62.8, 61.7, 61.6, 61.4, 58.3, 58.2, 56.1, 56.0, 55.8, 55.7, 55.6, 55.6, 54.9, 54.2, 54.1, 53.9, 53.9, 53.5, 53.5, 53.4, 51.1, 50.8, 49.0, 49.0, 36.2, 35.3, 35.3, 35.1, 34.7, 31.3, 29.1, 29.0, 28.8, 28.7, 28.7, 28.6, 28.5, 26.6, 26.5, 25.1, 22.1, 15.0, 13.9. **HRMS** (ESI) *m/z*: (C_33_H_34_N_7_O_6_^+^ [M+H]^+^) calc.: 624.2565, found: 624.2564.

* Due to residual water in the DMSO-*d_6_* the NMR corresponds to a complex mixture of the imine form, the hemiaminal forms (diastereomers) and the mixed forms.

**(11aS,11a'S)-8,8'-(((1H-1,2,3-triazole-1,4-diyl)bis(ethane-2,1-diyl))bis(oxy))bis(7-methoxy-2-methylene-1,2,3,11a-tetrahydro-5H-benzo[e]pyrrolo[1,2-a][1,4]diazepin-5-one) (D2)**

(*S*)-8-(2-azidoethoxy)-7-methoxy-2-methylene-1,2,3,11a-tetrahydro-5H-benzo[e]pyrrolo [1,2-a][1,4]diazepin-5-one (7.0 mg, 24.0 µmol, 1.0 eq.) and (*S*)-7-methoxy-2-methylene-8-(prop-2-yn-1-yloxy)-1,2,3,11a-tetrahydro-5H-benzo[e]pyrrolo[1,2-a][1,4]diazepin-5-one (8.0 mg, 24.0 µmol, 1.0 eq.) were dissolved in DMF (0.4 mL) and stirred at room temperature.

480 µL of a sodium ascorbate solution (100 mm, 48.0 µmol, 2.0 eq.) and 120 µL of a CuSO_4_ solution (100 mm, 12.0 µmol, 0.5 eq.) were mixed, 120 µL of a THPTA solution (100 mm, 12.0 µmol, 0.5 eq.) was added to the solution. The mixture was added to the previously prepared solution. After 1 h EtOAc and H_2_O were added to the mixture. The phases were separated and the aqueous phase was extracted with DCM and EtOAc. The combined organic layers were dried over Na_2_SO_4_, filtered, and concentrated under reduced pressure. Purification by preparative TLC (4% MeOH/DCM) gave the title compound as a colorless solid (3.2 mg, 5.0 µmol, 21%). **TLC**: *R*_f_ = 0.40 (MeOH/DCM, 1%) [UV] **^1^H NMR** (700 MHz, DMSO)*: δ [ppm] = 8.04 – 8.02 (m, 1H), 7.95 (s, 1H), 7.77 – 7.76 (m, 1H), 7.31 – 7.29 (m, 1H), 7.25 – 7.22 (m, 1H), 7.20 – 7.17 (m, 1H), 7.04 – 7.00 (m, 1H), 6.87 – 6.84 (m, 1H), 6.83 – 6.81 (m, 0.4H), 6.62 – 6.56 (m, 1H), 6.40 – 6.34 (m, 2H), 6.11 (s, 0.2H), 5.98 – 5.96 (m, 0.4H), 5.75 (s, 0.2H), 5.50 – 5.47 (m, 0.8H), 5.14 (d, *J* = 9.1 Hz, 2H), 5.10 (d, *J* = 8.3 Hz, 1H), 5.07 (d, *J* = 12.1 Hz, 1H), 4.97 – 4.93 (m, 3H), 4.86 (t, *J* = 6.1 Hz, 0.6H), 4.76 (brs, 4H), 4.54 – 4.50 (m, 2H), 4.44 – 4.29 (m, 5H), 4.25 – 4.08 (m, 11H), 3.96 (d, *J* = 15.6 Hz, 1H), 3.88 – 3.82 (m, 2H), 3.80 – 3.73 (m, 4H), 3.67 – 3.55 (m, 9H), 3.35 (s, 0.6H), 3.29 (s, 1H), 3.20 (s, 3H), 3.14 – 3.12 (m, 3H), 3.06 – 2.97 (m, 3H), 2.69 – 2.64 (m, 2H), 2.55 – 2.51 (m, 0.6H), 2.47 – 2.40 (m, 0.8H), 1.71 (d, *J* = 39.7 Hz, 0.6H), 1.23 (brs, 1H). **^13^C NMR** (176 MHz, DMSO)*: δ [ppm] = 164.5, 164.4, 163.5, 163.5, 162.3, 151.5, 151.0, 150.1, 147.0, 1.43.8, 143.3, 142.7, 142.6, 140.7, 140.6, 140.5, 139.3, 123.3, 120.7, 120.0, 119.4, 111.6, 111.3, 110.4, 110.1, 109.5, 108.5, 106.0, 102.2, 101.6, 88.4, 88.3, 67.4, 67.1, 66.9, 66.7, 59.1, 58.3, 56.1, 55.9, 55.7, 55.6, 54.9, 54.2, 53.9, 53.5, 53.4, 53.1, 51.1, 48.8, 36.2, 34.7, 30.7, 25.4. **HRMS** (ESI) *m/z*: (C_34_H_36_N_7_O_6_^+^ [M+H]^+^) calc.: 638.2722, found: 638.2723.

* Due to residual water in the DMSO-*d_6_* the NMR corresponds to a complex mixture of the imine form, the hemiaminal forms (diastereomers) and the mixed forms.

**(11aS)-7-methoxy-8-(2-(4-(2-(((S)-7-methoxy-2-methylene-5-oxo-2,3,5,11a-tetrahydro-1H-benzo[e]pyrrolo[1,2-a][1,4]diazepin-8-yl)oxy)ethoxy)-4,5,6,7,8,9-hexahydro-1H-cycloocta[d][1,2,3]triazol-1-yl)ethoxy)-2-methylene-1,2,3,11a-tetrahydro-5H-benzo[e]pyrrolo[1,2-a][1,4]diazepin-5-one (D3)**

(*S*)-8-(2-azidoethoxy)-7-methoxy-2-methylene-1,2,3,11a-tetrahydro-5H-benzo[e]pyrrolo[1,2-a][1,4]diazepin-5-one (5.0 mg, 15.0 µmol, 1.0 eq.) and (11a*S*)-8-(2-(cyclooct-2-yn-1-yloxy)ethoxy)-7-methoxy-2-methylene-1,2,3,11a-tetrahydro-5H-benzo[e]pyrrolo[1,2-a][1,4]diazepin-5-one (6.1 mg, 15.0 µmol, 1.0 eq.) were dissolved in 0.5 mL ACN. The mixture was stirred at room temperature over night. The solvent was removed under reduced pressure. Purification by column chromatography (silica, MeOH/DCM 0% 🡪 5%) yielded the title compound as a colorless solid (7.74 mg, 10.5 µmol, 70%). **TLC**: *R*_f_ = 0.47 (MeOH/DCM, 1%) [UV] **^1^H NMR** (700 MHz, DMSO)*: δ [ppm] = 7.76 – 7.68 (m, 1H), 7.32 – 7.22 (m, 1H), 7.11 – 7.04 (m, 1H), 6.82 – 6.76 (m, 1H), 6.56 – 6.45 (m, 1H), 6.18 – 6.00 (m, 1H), 5.48 – 5.40 (m, 1H), 5.14 – 4.99 (m, 6H), 4.86 – 4.62 (m, 3H), 4.54 – 4.22 (m, 5H), 4.14 – 4.09 (m, 5H), 3.84 – 3.56 (m, 14H), 3.10 – 2.78 (m, 8H), 2.28 – 1.99 (m, 3H), 1.81 – 1.67 (m, 3H), 1.56 – 1.48 (m, 7H), 1.22 – 1.03 (m, 4H). **^13^C NMR** (176 MHz, DMSO)*^,^**: δ [ppm] = 1.74.4, 165.2, 164.3, 161.8, 1.61.3, 151.8, 151.5, 151.2, 150.6, 147.0, 145.9, 145.3, 143.4, 142.9, 142.2, 141.9, 140.4, 137.5, 135.2, 130.7, 127.9, 121.05, 120.7, 120.6, 118.2, 113.2, 112.7, 102.4, 73.9, 66.9, 66.1, 62.4, 55.8, 55.6, 54.9, 54.7, 51.9, 48.1, 46.2, 39.4, 35.1, 34.9, 34.5, 34.2, 31.0, 30.3, 29.7, 28.9, 28.7, 27.0, 25.5, 24.4, 22.0, 20.7, 18.2, 7.0. **HRMS** (ESI) *m/z*: (C_40_H_46_N_7_O_7_^+^ [M+H]^+^) calc.: 736.3453, found: 736.3454.

* Due to residual water in the DMSO-*d_6_* the NMR corresponds to a complex mixture of the imine form, the hemiaminal forms (diastereomers) and the mixed forms.

**Determined by HMBC.

**(S)-7-methoxy-2-methylene-5-oxo-2,3,5,11a-tetrahydro-1H-benzo[e]pyrrolo[1,2-a] [1,4]diazepin-8-yl (((5aS,6R,6aR)-1-(2-(((S)-7-methoxy-2-methylene-5-oxo-2,3,5,11a-tetrahydro-1H-benzo[e]pyrrolo[1,2-a][1,4]diazepin-8-yl)oxy)ethyl)-1,4,5,5a,6,6a,7,8-octahydrocyclopropa[5,6]cycloocta[1,2-d][1,2,3]triazol-6-yl)methyl) carbonate (D4)**

(*S*)-8-(2-azidoethoxy)-7-methoxy-2-methylene-1,2,3,11a-tetrahydro-5H-benzo[e]pyrrolo[1,2-a][1,4]diazepin-5-one (5.0 mg, 15.0 µmol, 1.0 eq.) and bicyclo[6.1.0]non-4-yn-9-ylmethyl((*S*)-7-methoxy-2-methylene-5-oxo-2,3,5,11a-tetrahydro-1H-benzo[e]pyrrolo[1,2-a][1,4]diazepin-8-yl) carbonate (6.5 mg, 15.0 µmol, 1.0 eq.) were dissolved in 0.5 mL MeOH. The mixture was stirred at room temperature over night. The solvent was removed under reduced pressure. Purification by column chromatography (silica, MeOH/DCM 0% 🡪 10%) yielded the title compound as a colorless solid (5.3 mg, 6.97 µmol, 46%). **TLC**: *R*_f_ = 0.31 (MeOH/DCM, 1%) [UV] **^1^H NMR** (700 MHz, DMSO)*: δ [ppm] = 7.83 (d, *J* = 4.4 Hz, 1H), 7.76 (d, *J* = 2.9 Hz, 1H), 7.51 (s, 1H), 7.31 (d, *J* = 2.60, 1H), 7.21 (s, 1H), 6.82 (d, *J* = 11.0 Hz, 1H), 5.16 – 5.12 (m, 4H), 5.06 – 4.89 (m, 2H), 4.68 (brs, 3H), 4.47 – 4.45 (m, 1H), 4.38 – 4.34 (m, 4H), 4.23 (t, *J* = 5.2 Hz, 1H), 4.20 – 4.17 (m, 2H), 4.12 – 4.08 (m, 4H), 3.96 – 3.94 (m, 1H), 3.87 (s, 3H), 3.84 – 3.83 (m, 1H), 3.79 (s, 3H), 3.69 (brs, 1H), 3.63 – 3.60 (m, 1H), 3.13 – 2.98 (m, 8H), 2.90 – 2.76 (m, 4H), 2.19 – 2.06 (m, 3H), 1.67 – 1.58 (m, 3H), 1.28 – 1.22 (m, 3H), 1.09 – 0.98 (m, 3H). **^13^C NMR** (176 MHz, DMSO)*: δ [ppm] = 165.7, 165.3, 152.6, 151.8, 146.5, 145.9, 144.7, 143.5, 142.5, 140.9, 140.5, 134.6, 128.5, 126.0, 121.7, 121.2, 120.4, 116.7, 115.9, 115.2, 113.6, 112.6, 107.8, 107.6, 102.9, 67.8, 56.5, 56.2, 55.4, 53.5, 53.4, 49.1, 47.0, 35.7, 35.6, 33.6, 32.5, 31.0, 27.4, 25.9, 22.7, 22.1, 21.4, 19.9, 19.4, 17.1. **HRMS** (ESI) *m/z*: (C_41_H_44_N_7_O_8_^+^ [M+H]^+^) calc.: 762.3246, found: 762.3245.

* Due to residual water in the DMSO-*d_6_* the NMR corresponds to a complex mixture of the imine form, the hemiaminal forms (diastereomers) and the mixed forms.

**NMR Spectra:**

**(*S*)-2-(methoxycarbonyl)-4-methylenepyrrolidiniumchloride (5)**


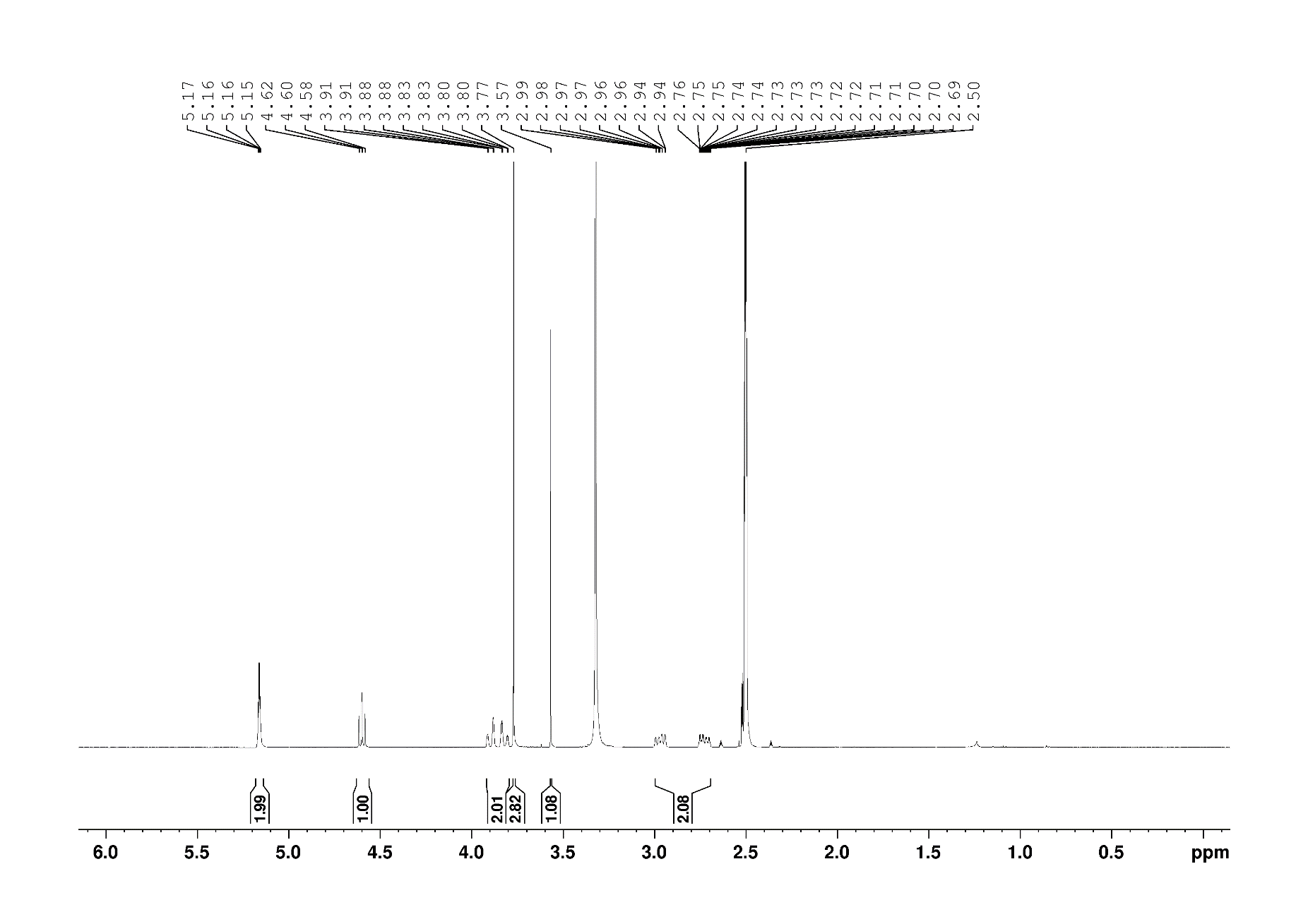


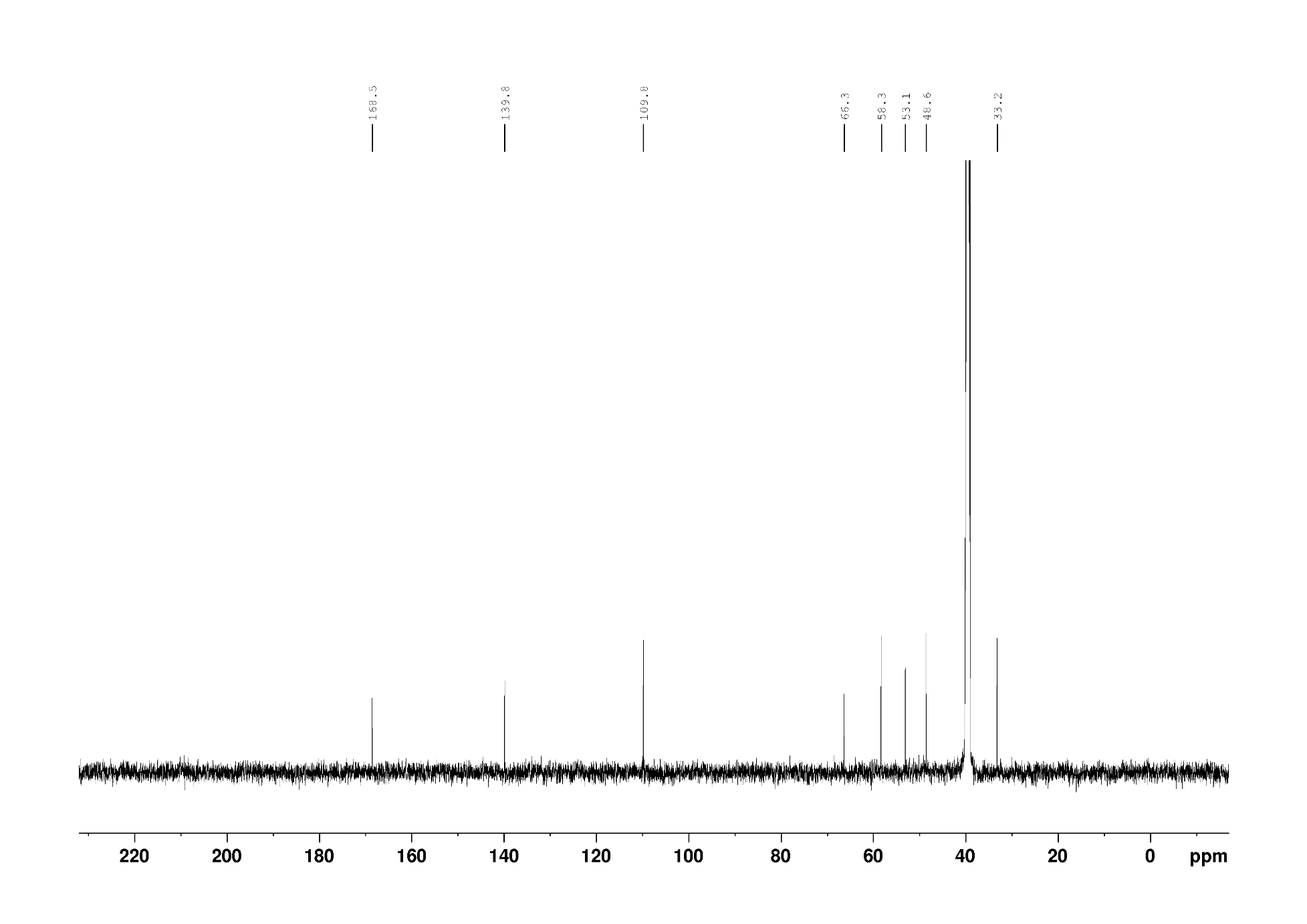


**4-(benzyloxy)-3-methoxybenzoic acid (6)**


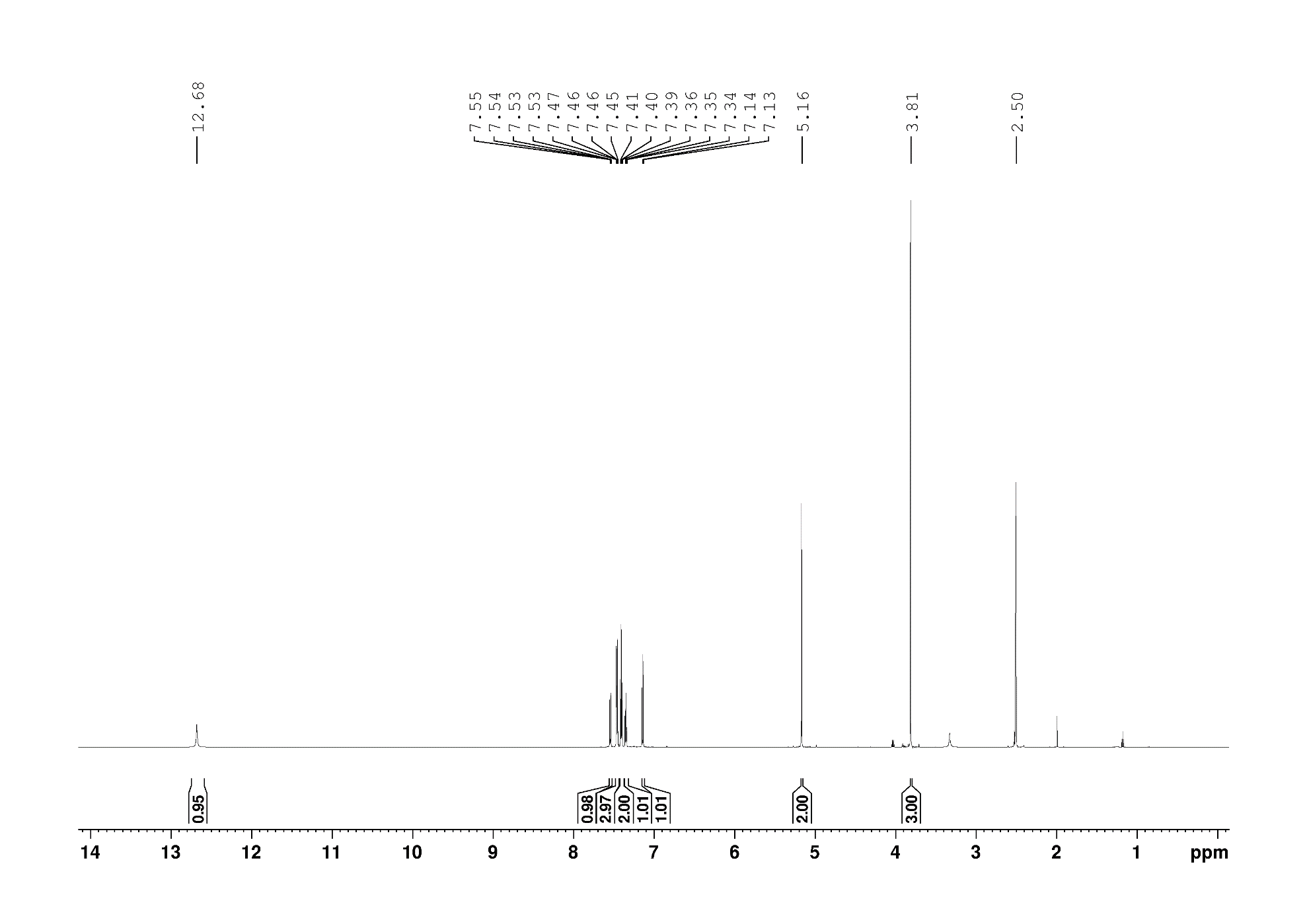


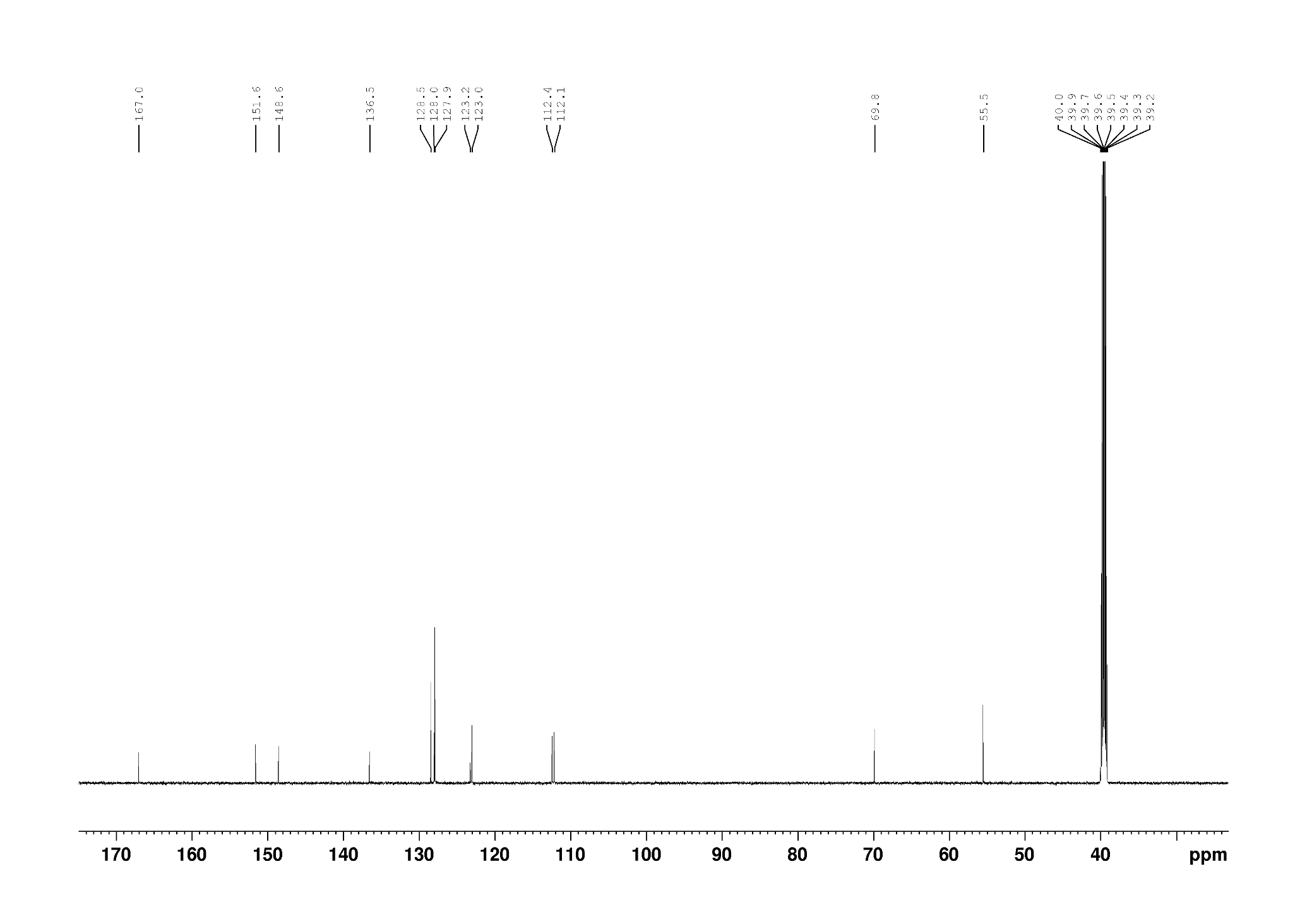


**4-(benzyloxy)-5-methoxy-2-nitrobenzoic acid (7)**


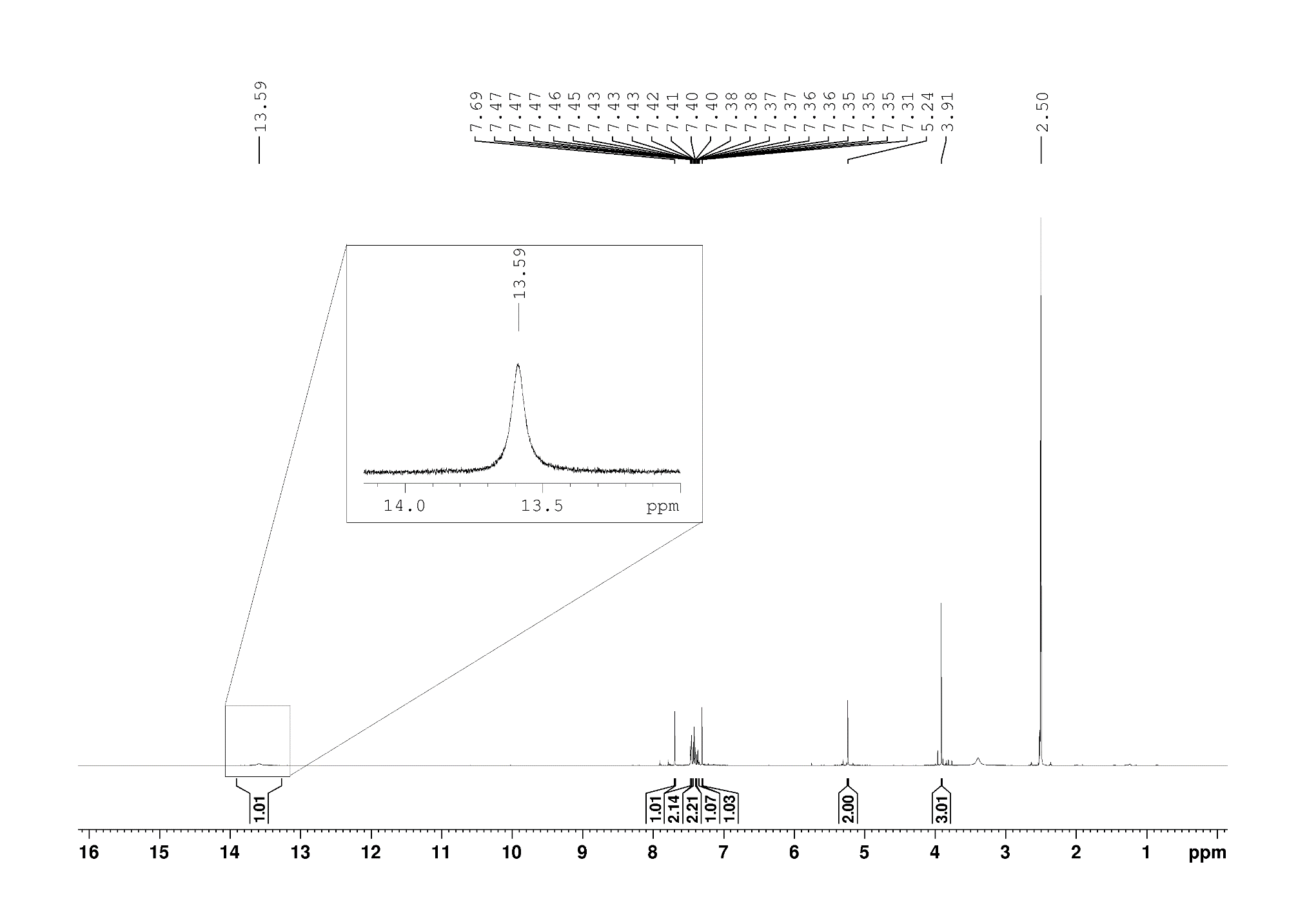


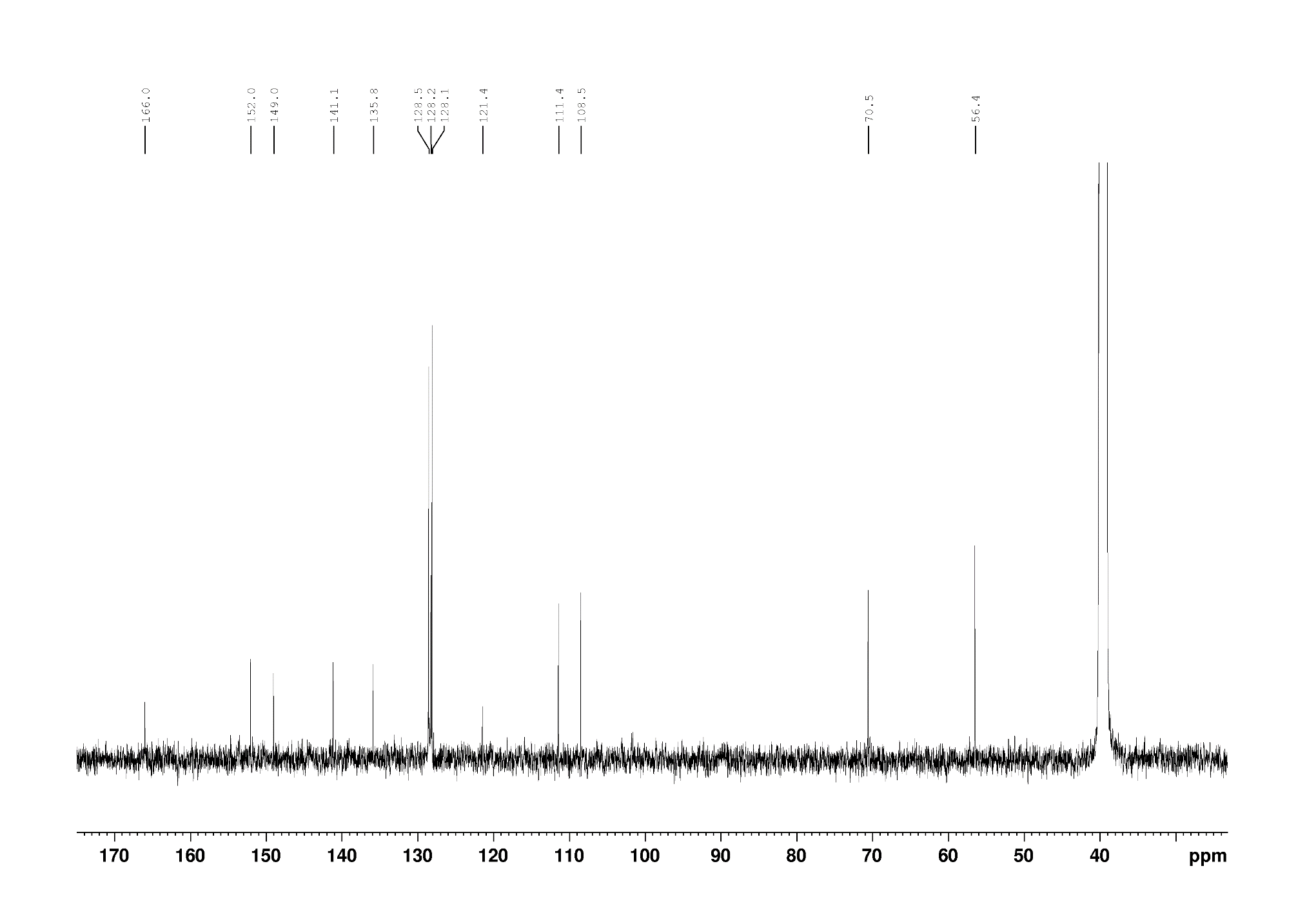


**2-amino-4-(benzyloxy)-5-methoxybenzoic acid (8)**


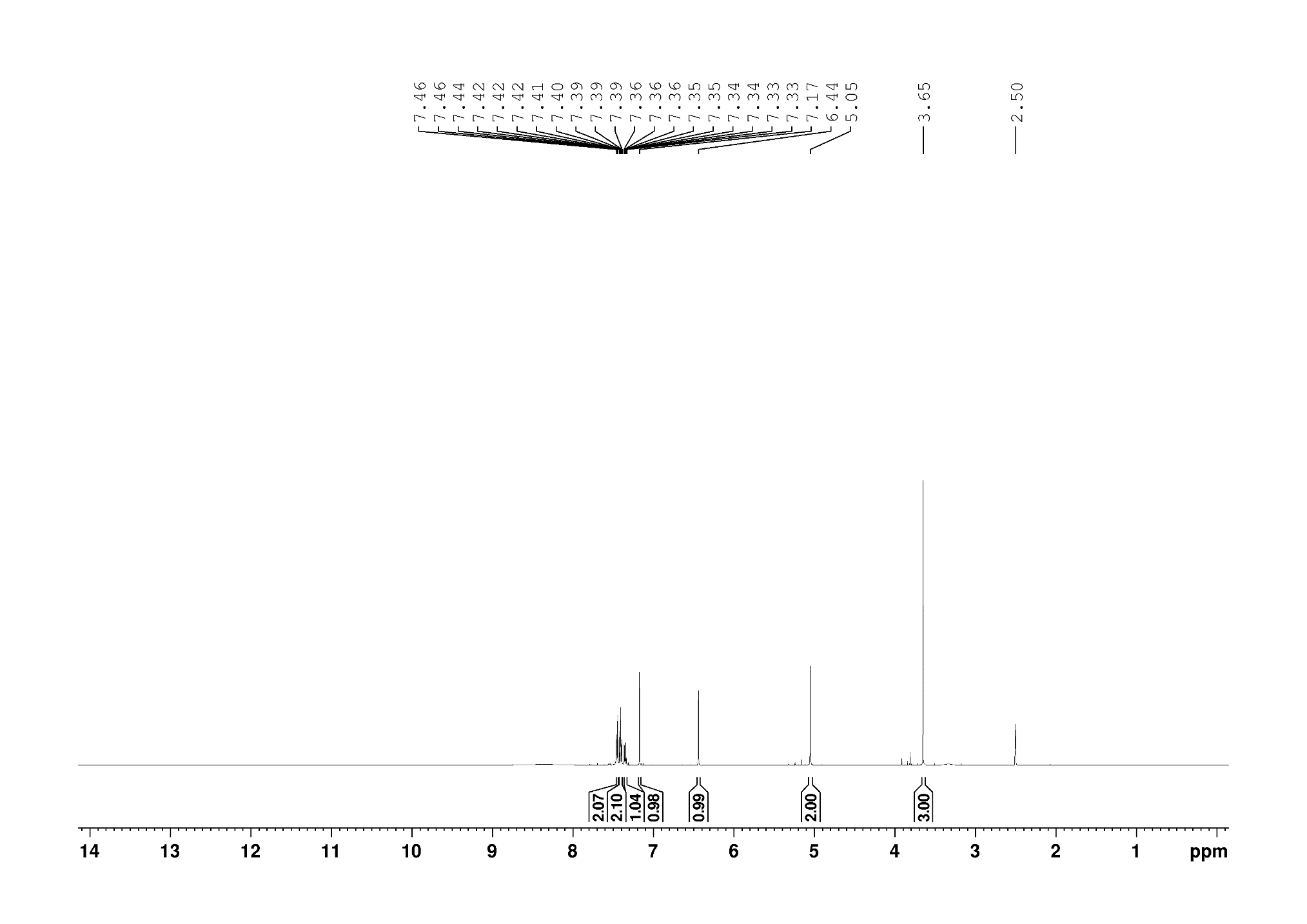


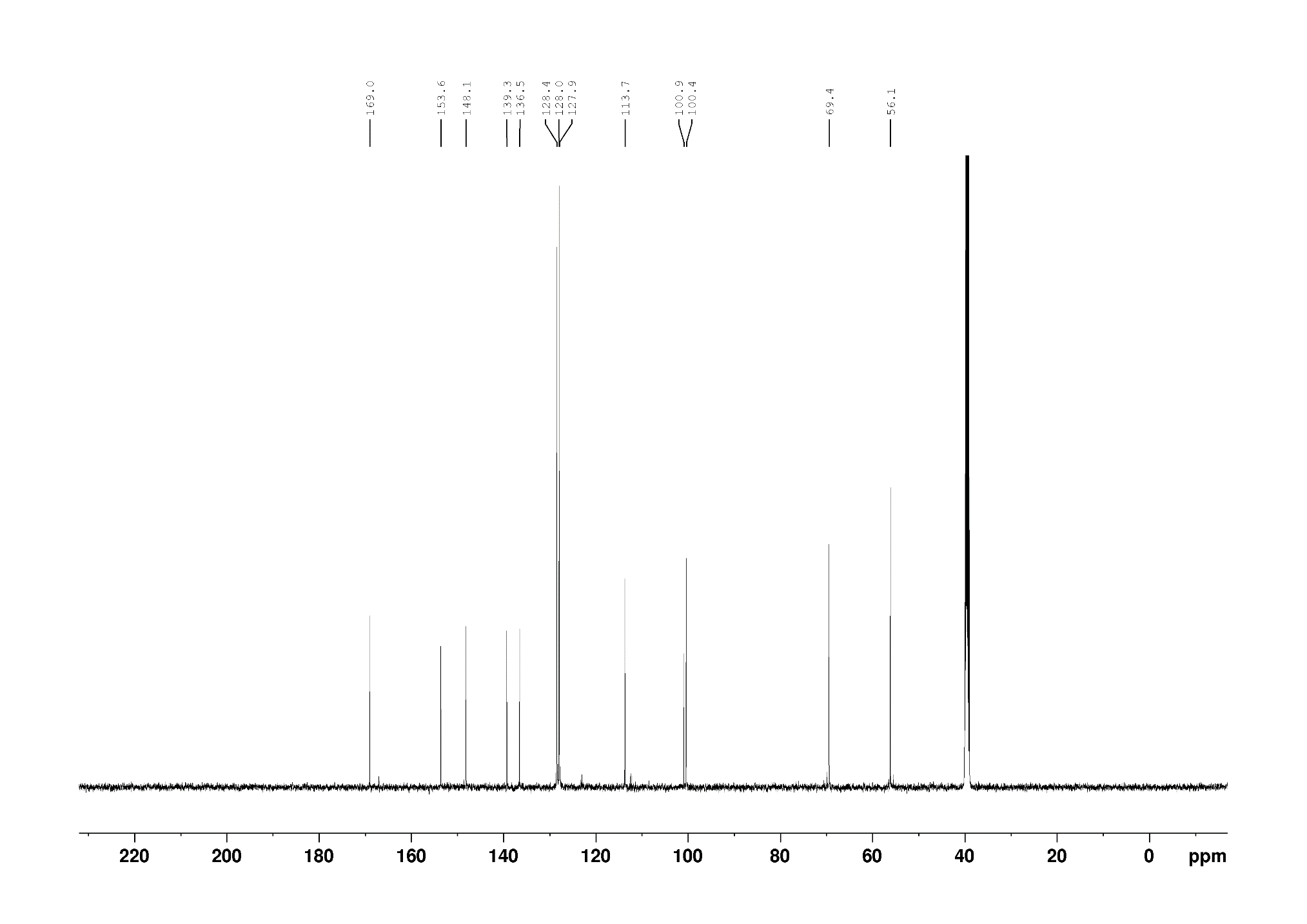


**4-(benzyloxy)-2-((*tert*-butoxycarbonyl)amino)-5-methoxybenzoic acid (9)**


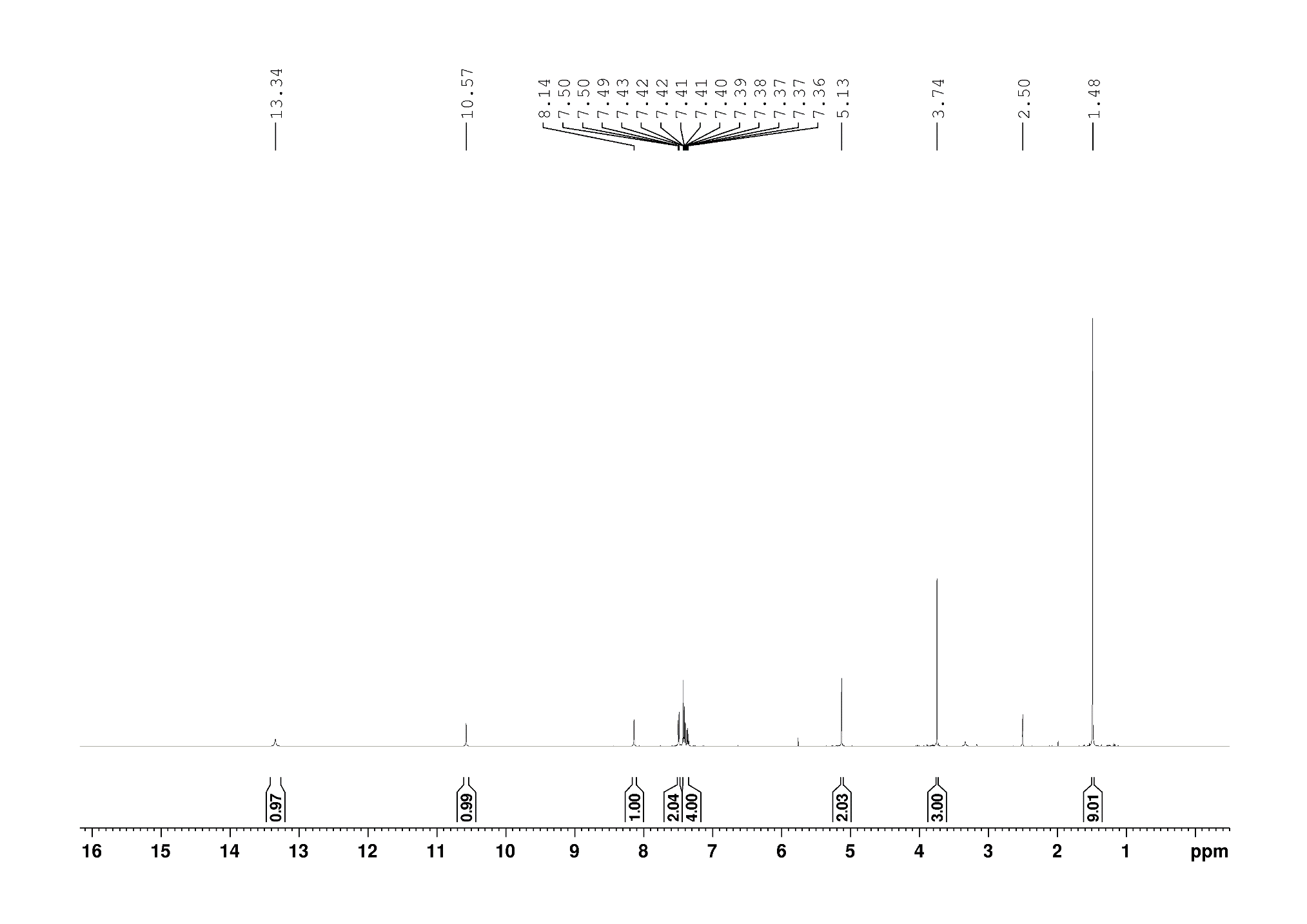


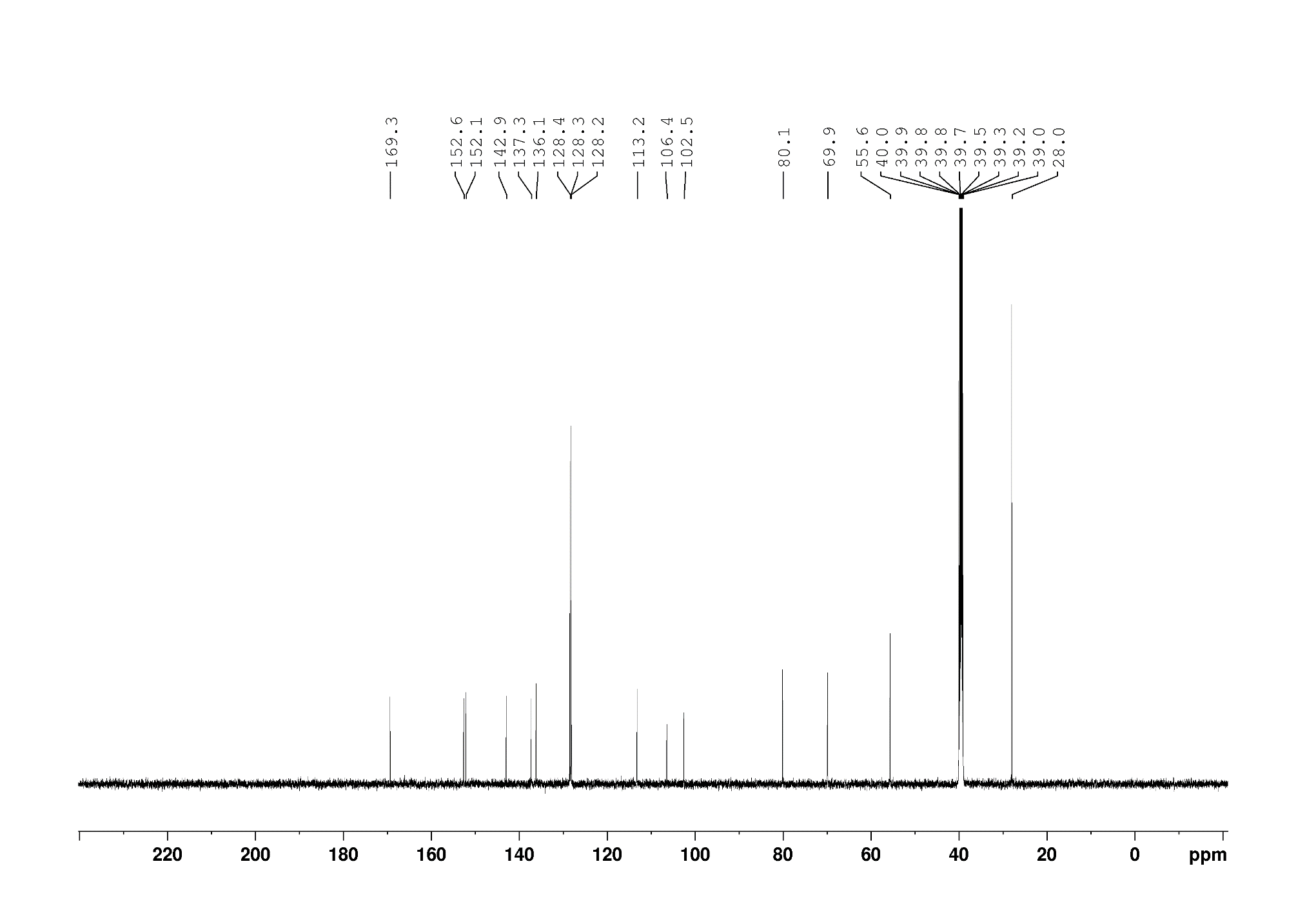


**methyl(*S*)-1-(4-(benzyloxy)-2-((*tert*-butoxycarbonyl)amino)-5-methoxybenzoyl)-4-methylenepyrrolidine-2-carboxylate (10)**


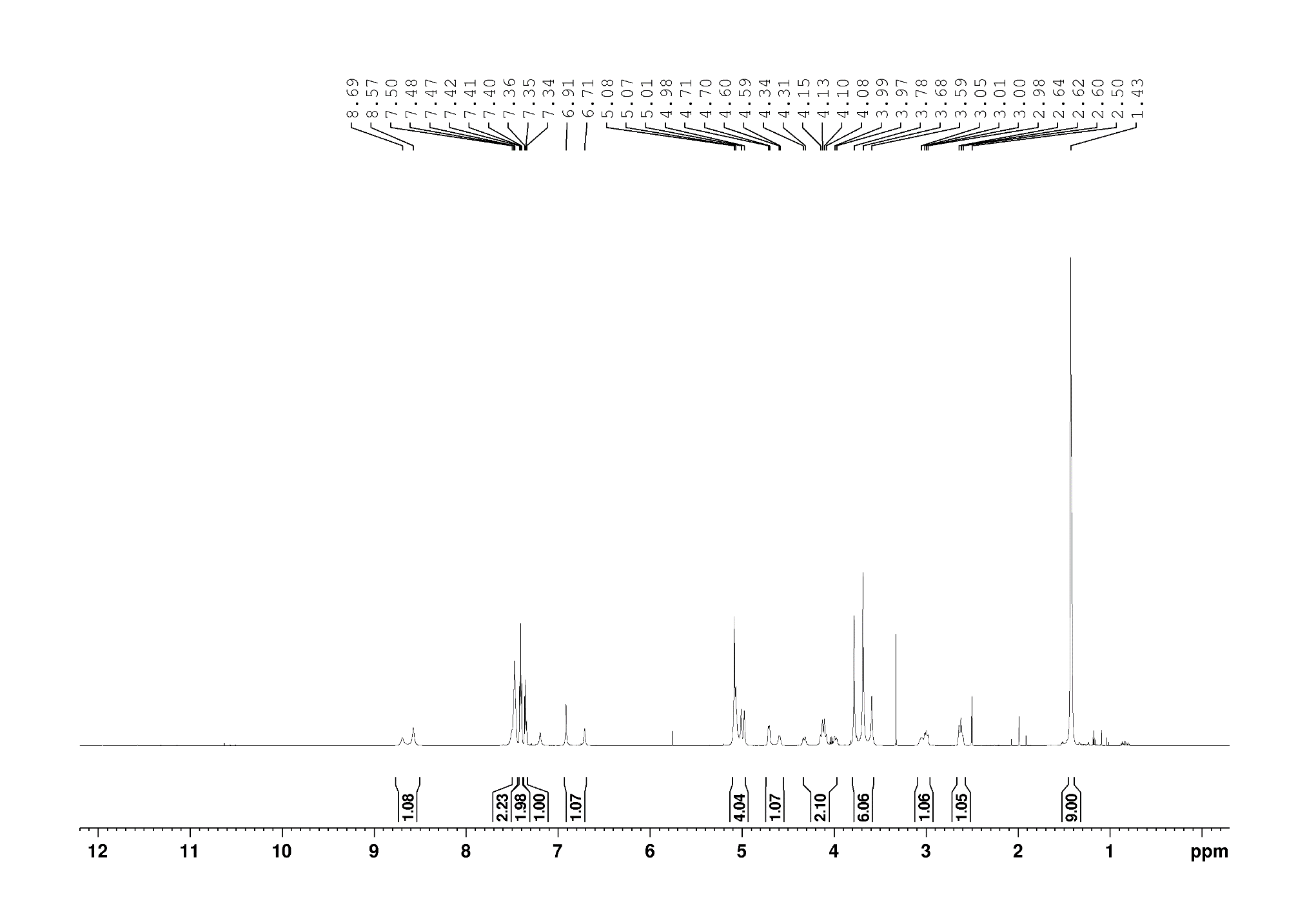


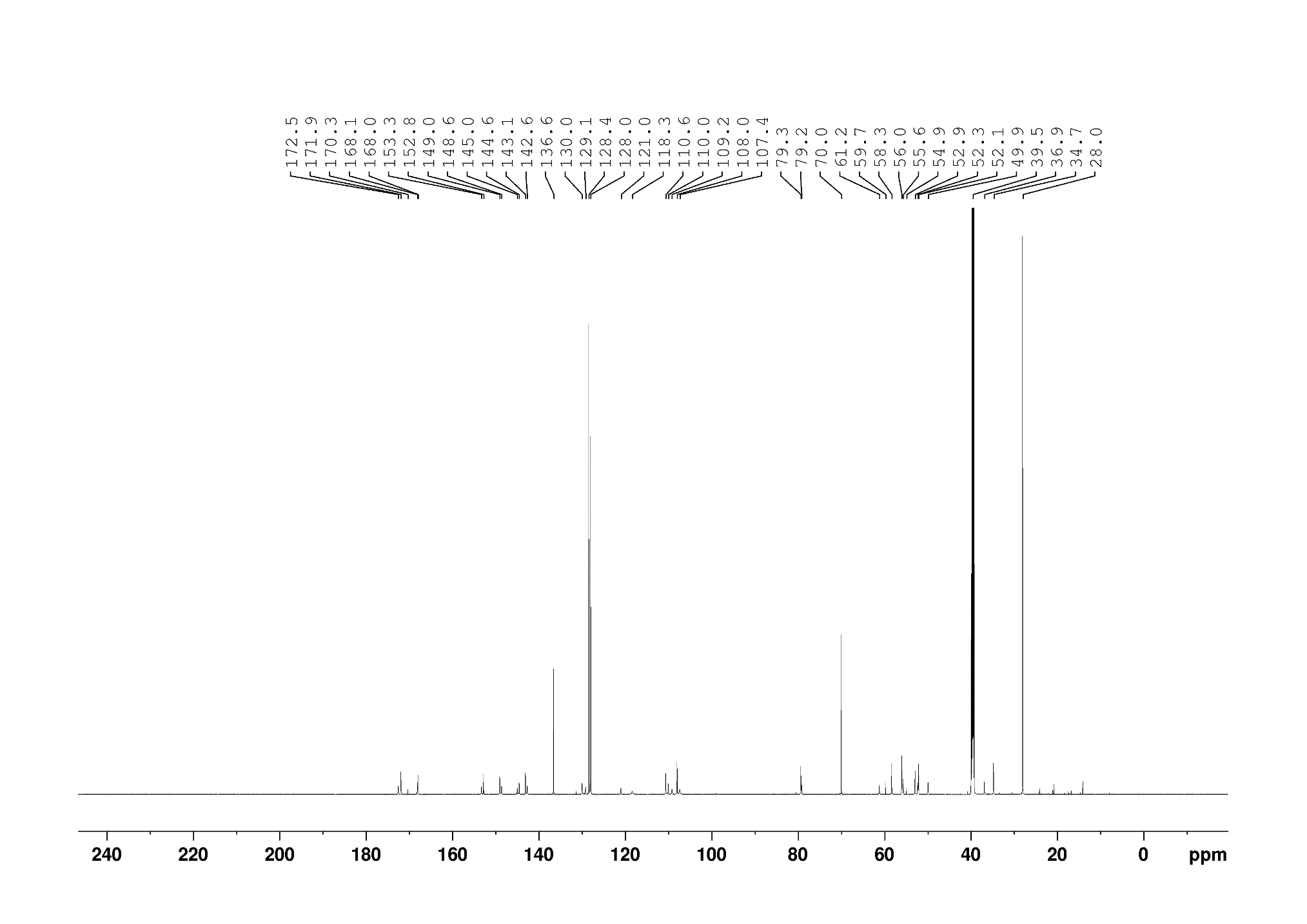


***tert*-butyl(*S*)-(5-(benzyloxy)-2-(2-formyl-4-methylenepyrrolidine-1-carbonyl)-4-methoxyphenyl)carbamate (11)**


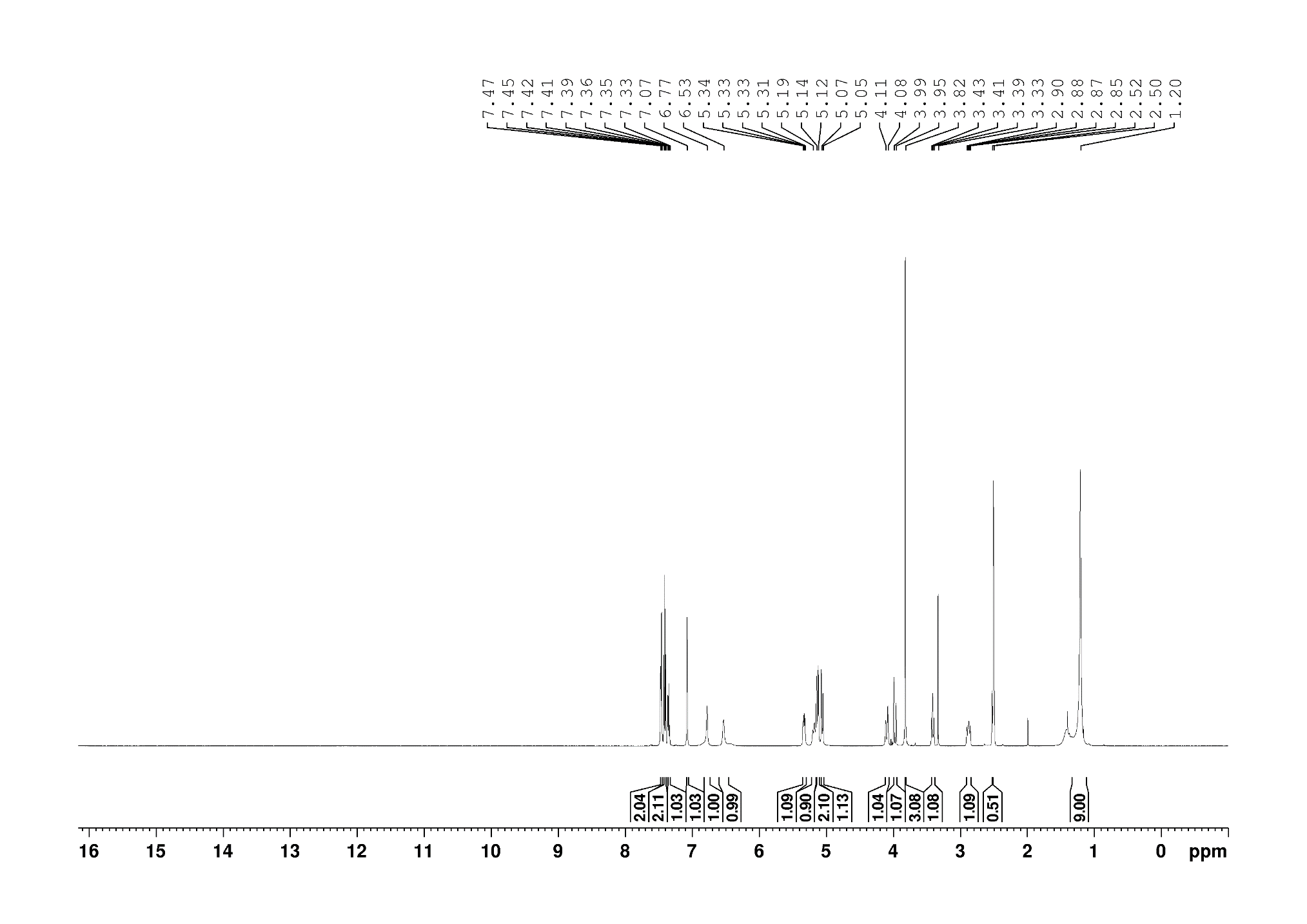


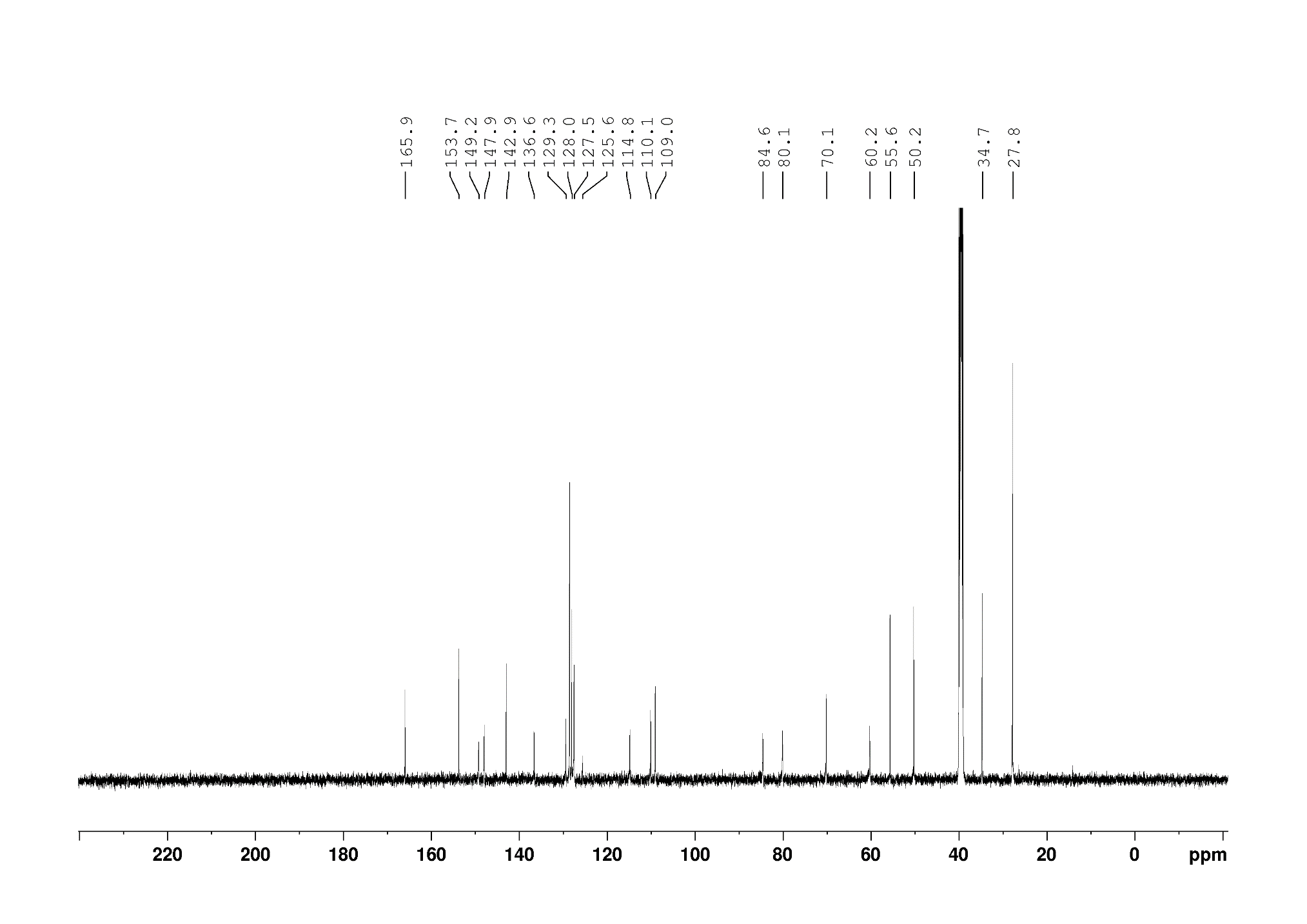


**(*S*)-8-hydroxy-7-methoxy-2-methylene-1,2,3,11a-tetrahydro-5H-benzo[e]pyrrolo[1,2-a][1,4]diazepin-5-one (13b)**


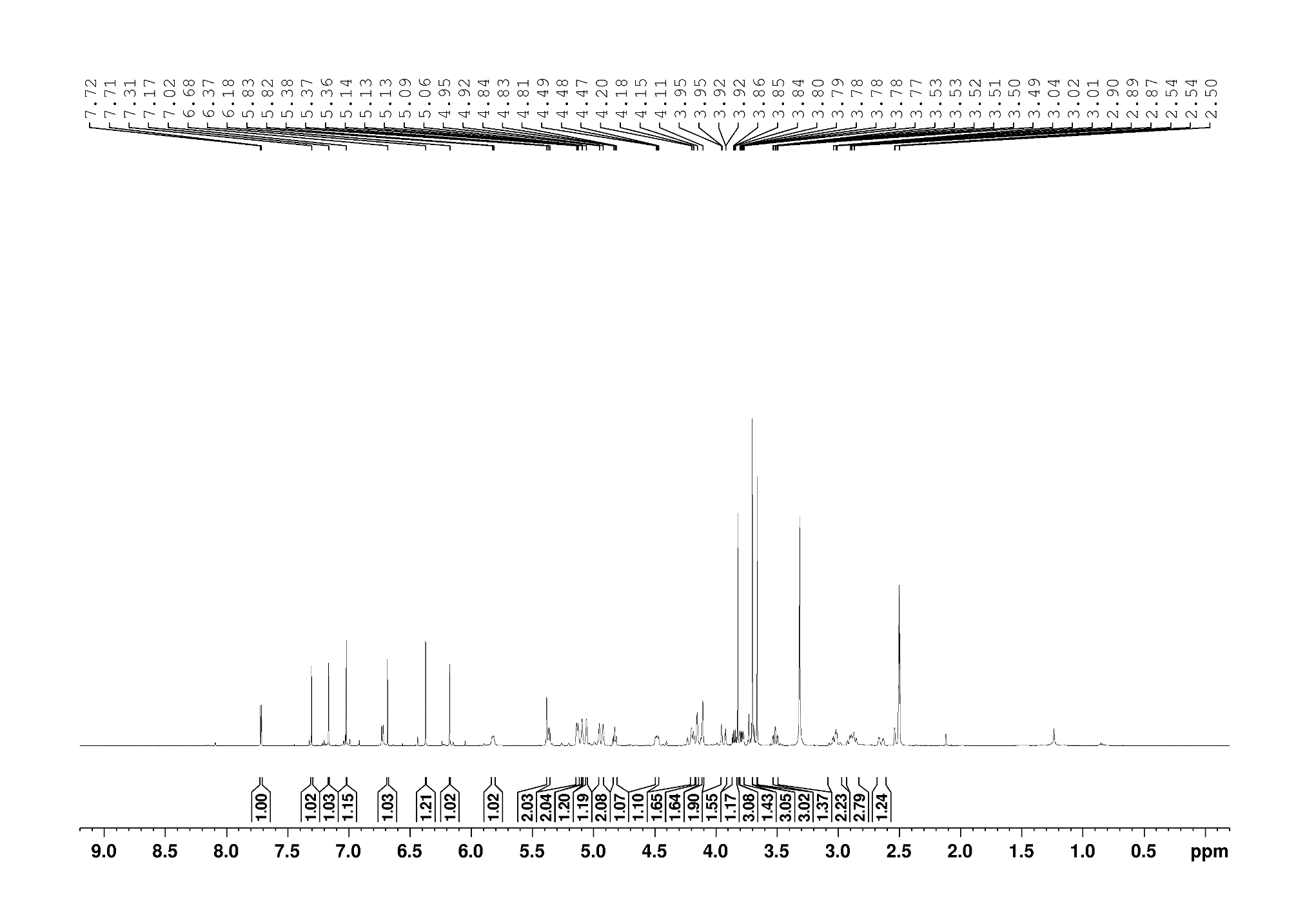


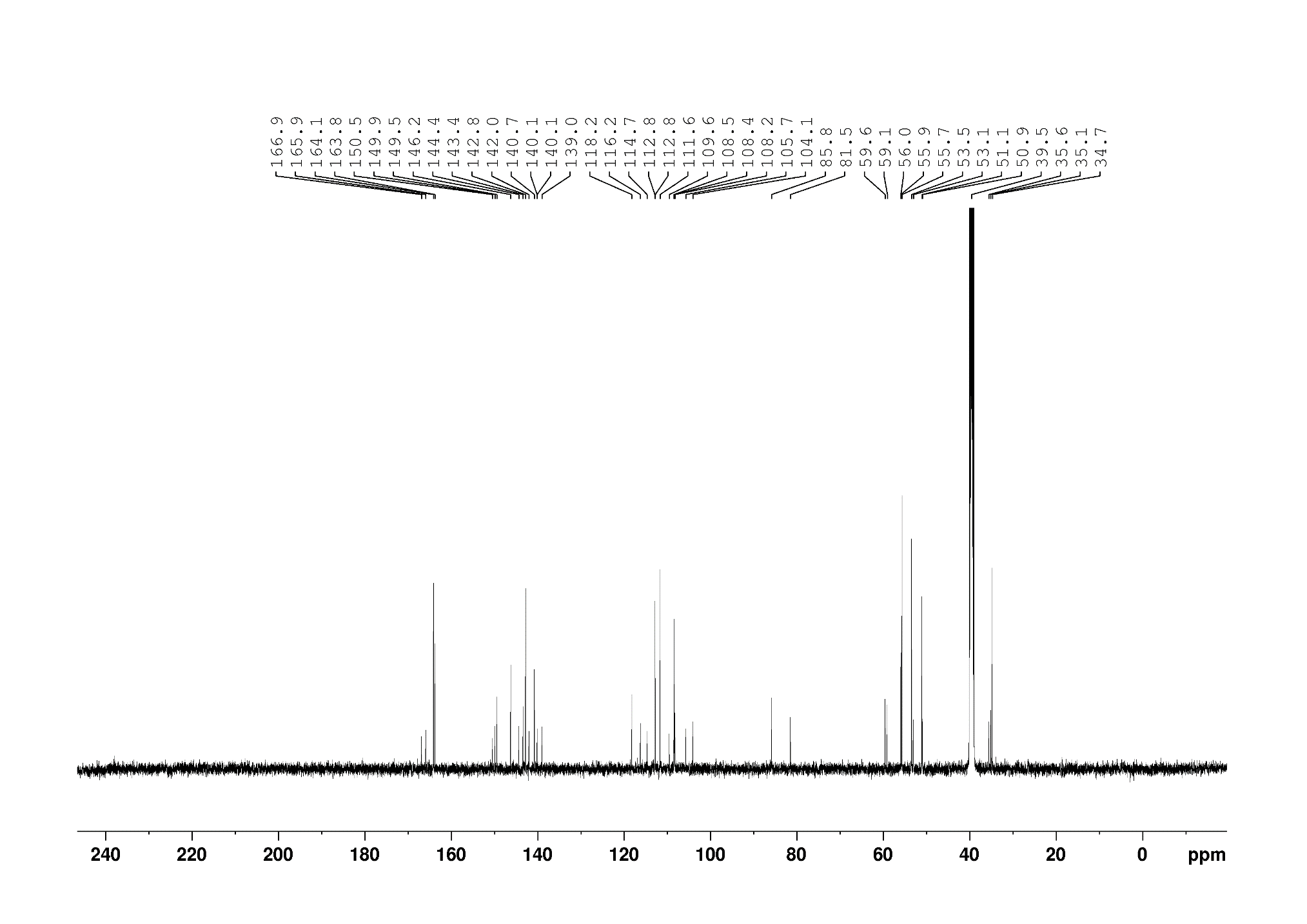


**(*S*)-8-(2-bromoethoxy)-7-methoxy-2-methylene-1,2,3,11a-tetrahydro-5H-benzo[e]pyrrolo[1,2-a][1,4]diazepin-5-one (14)**


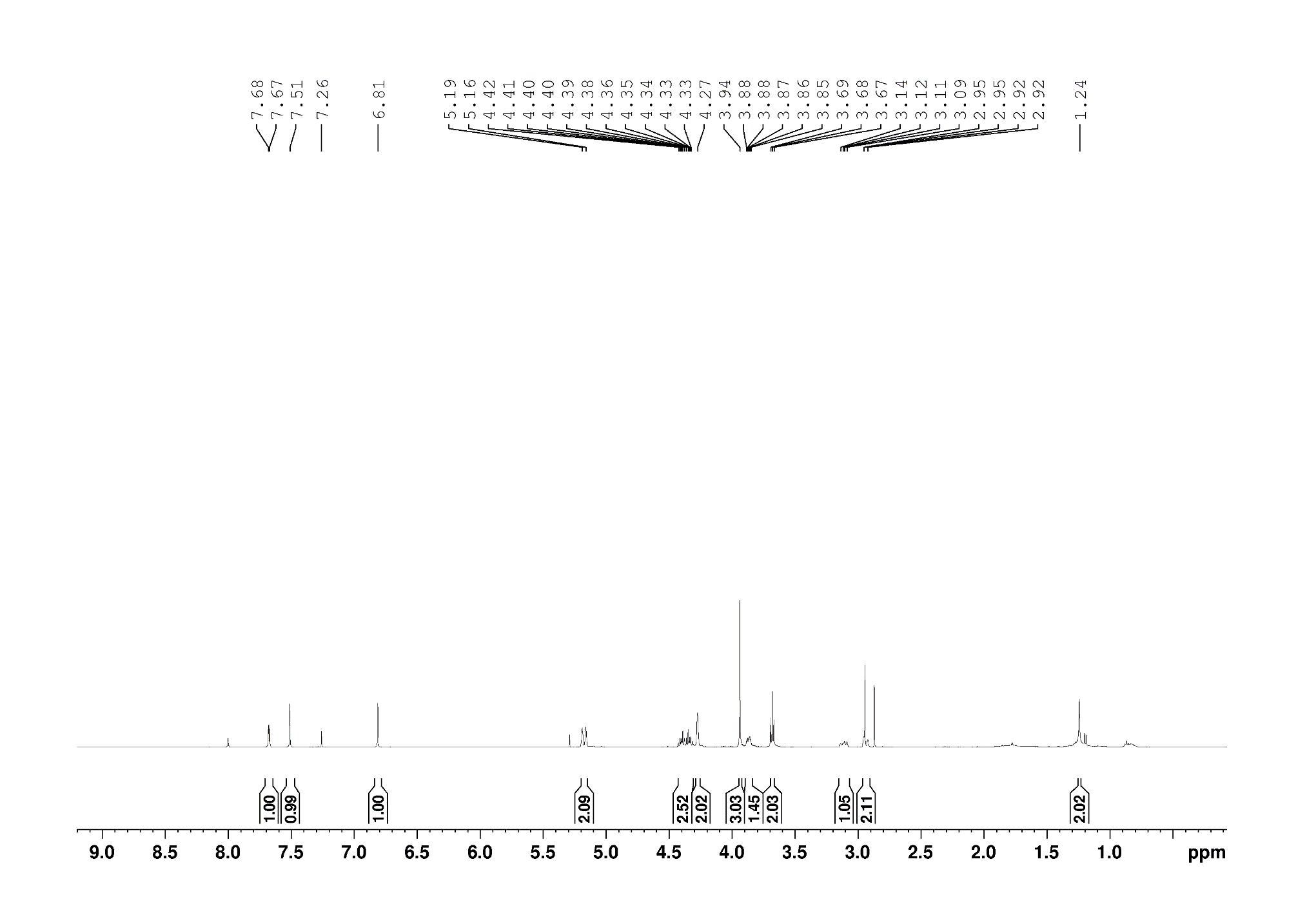


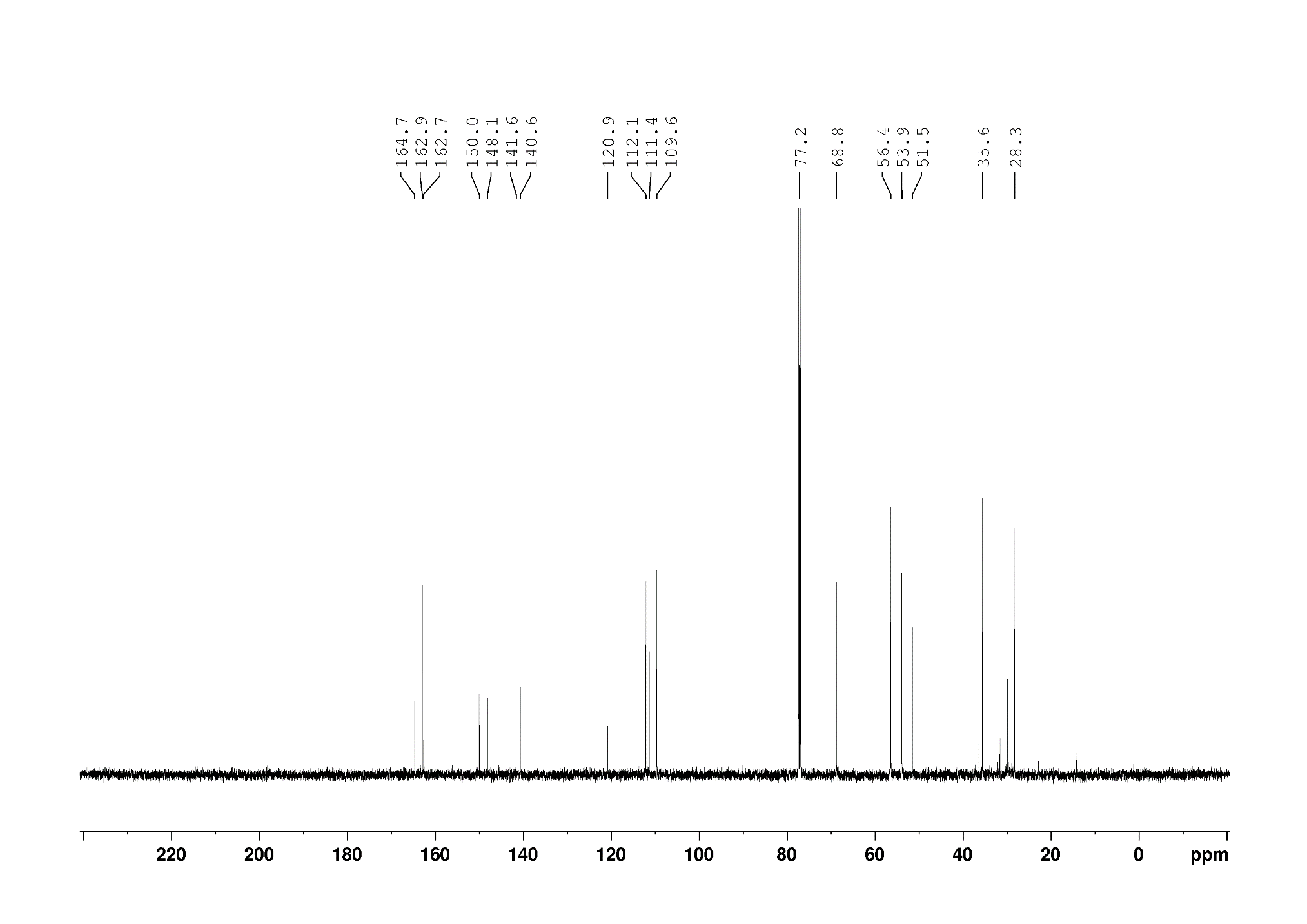


**(*S*)-8-(2-azidoethoxy)-7-methoxy-2-methylene-1,2,3,11a-tetrahydro-5H-benzo[e]pyrrolo[1,2-a][1,4]diazepin-5-one (MbA)**


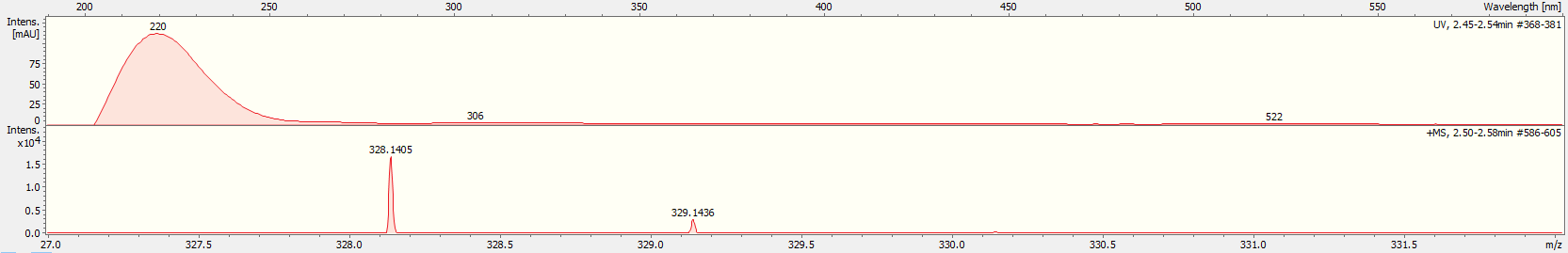


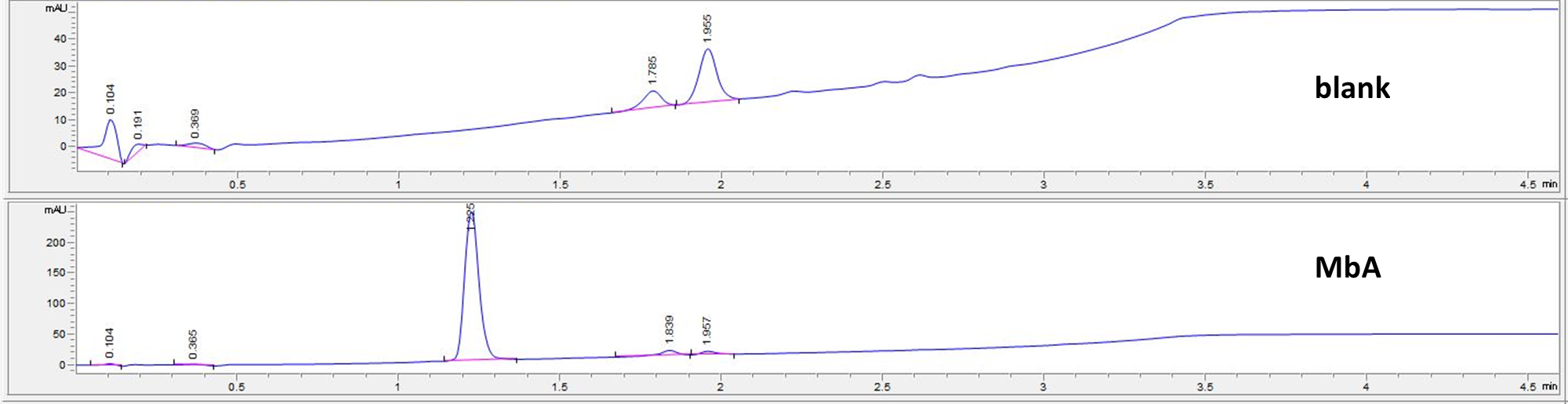


**95%** calculated purity by HPLC

//nmrxiv.org/project/zHJDzpEvl4KhtWqYMyFd63edQc3poLvawWErPIcA
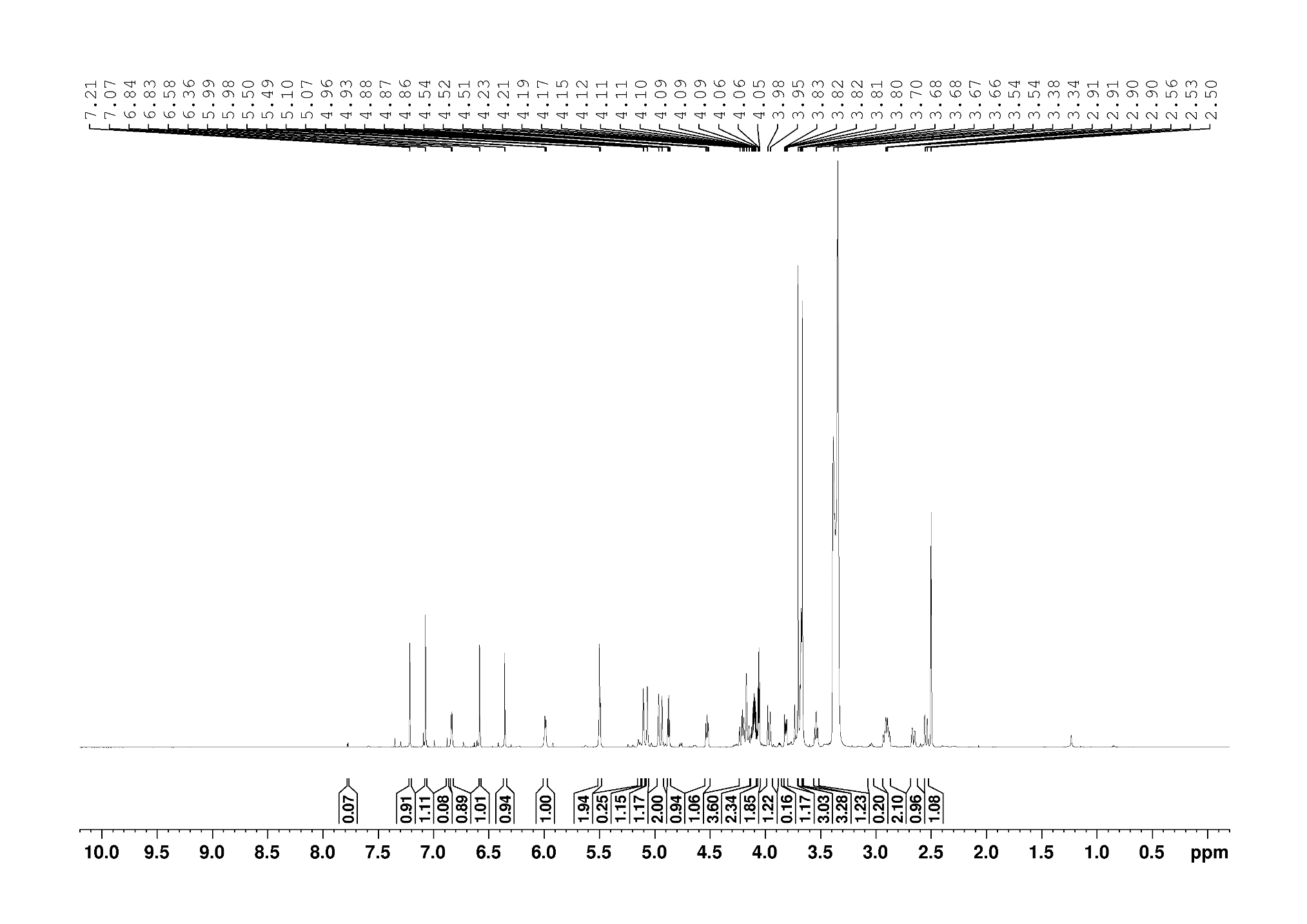


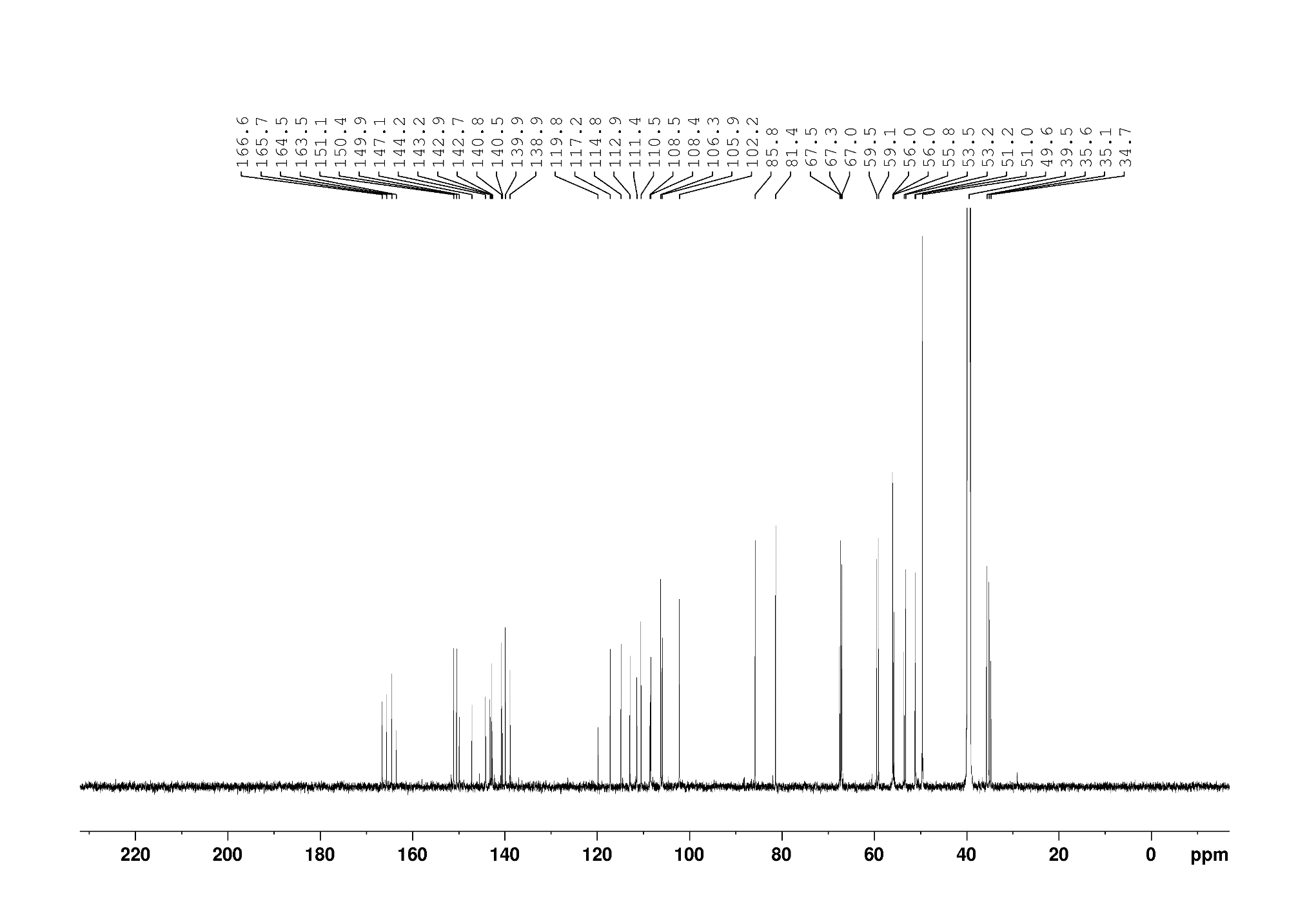


**(*S*)-7-methoxy-2-methylene-8-(prop-2-yn-1-yloxy)-1,2,3,11a-tetrahydro-5H-benzo[e]pyrrolo[1,2-a][1,4]diazepin-5-one (M1)**


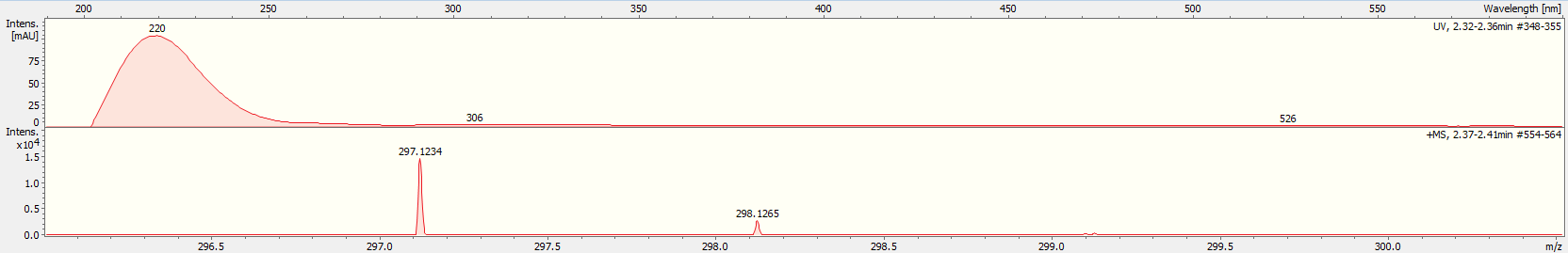


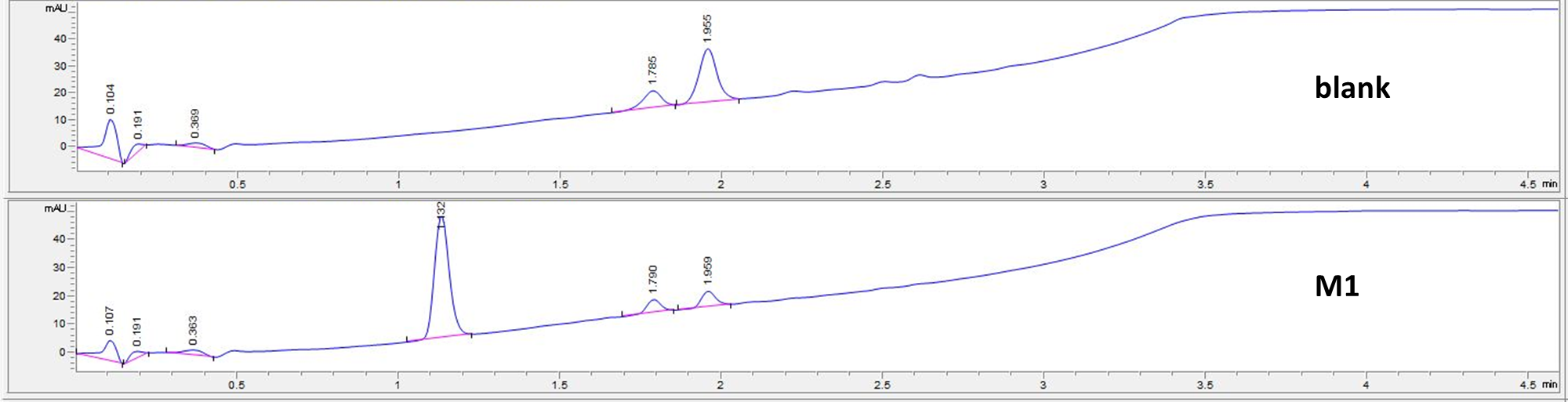


100% calculated purity by HPLC.

//nmrxiv.org/project/zHJDzpEvl4KhtWqYMyFd63edQc3poLvawWErPIcA


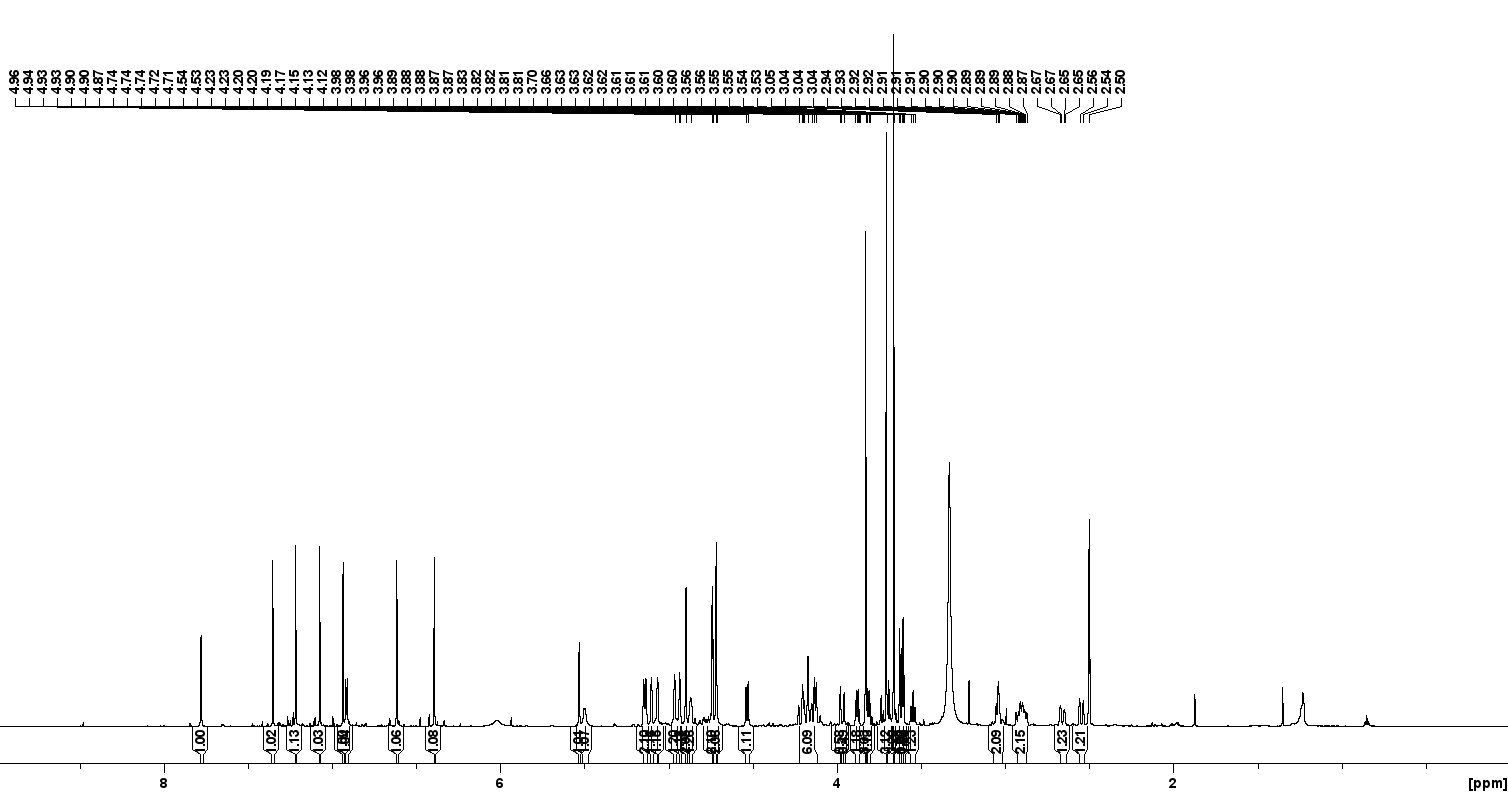


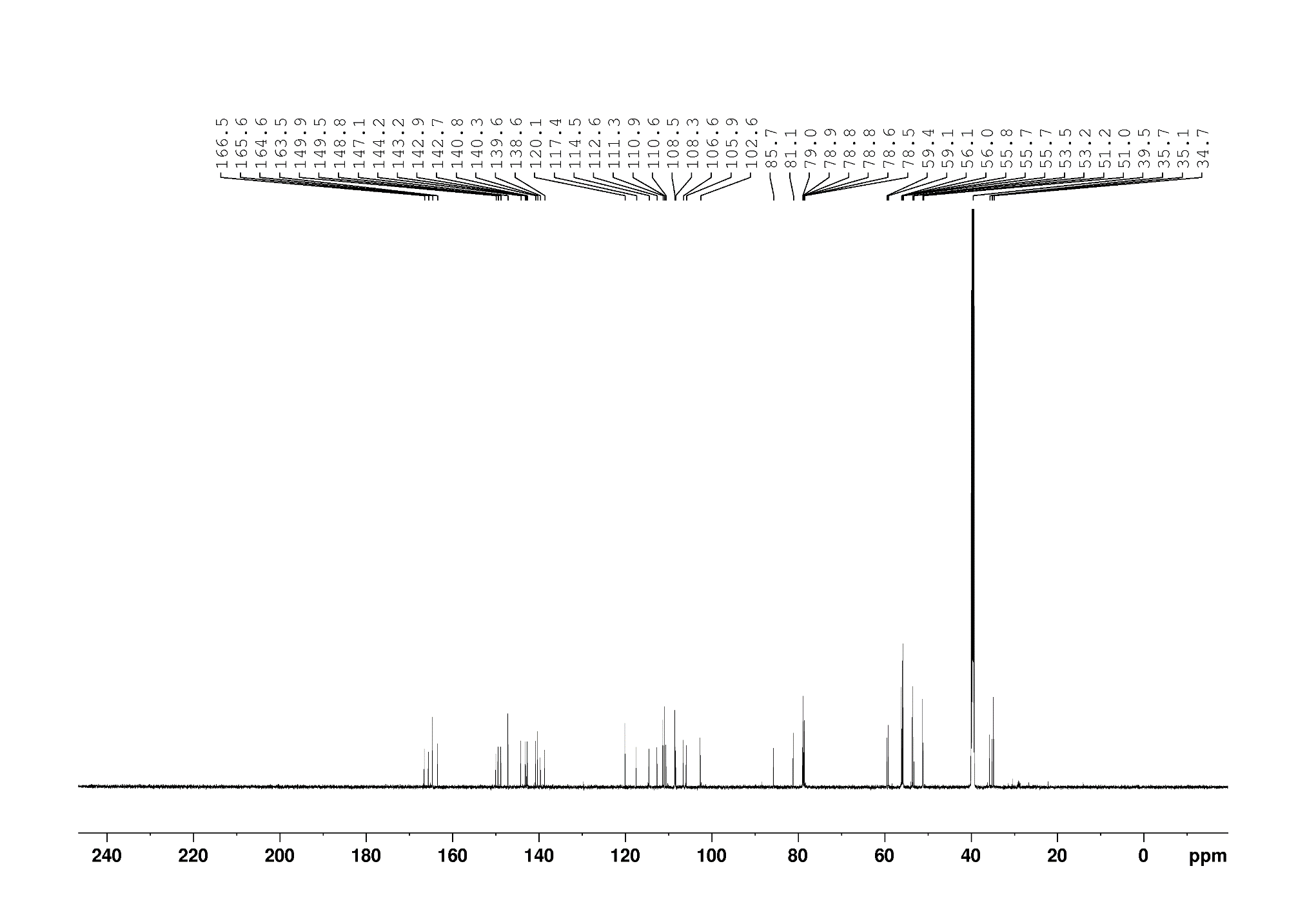


**(*S*)-8-(but-3-yn-1-yloxy)-7-methoxy-2-methylene-1,2,3,11a-tetrahydro-5H-benzo[e]pyrrolo[1,2-a][1,4]diazepin-5-one (M2)**


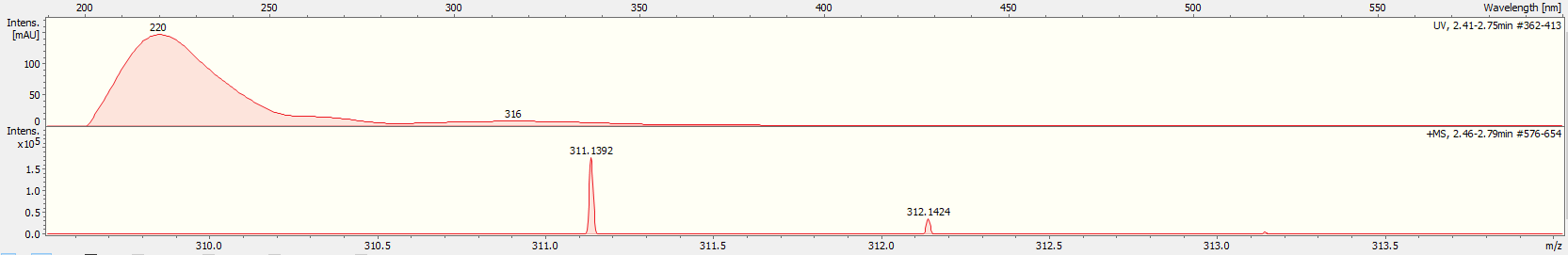


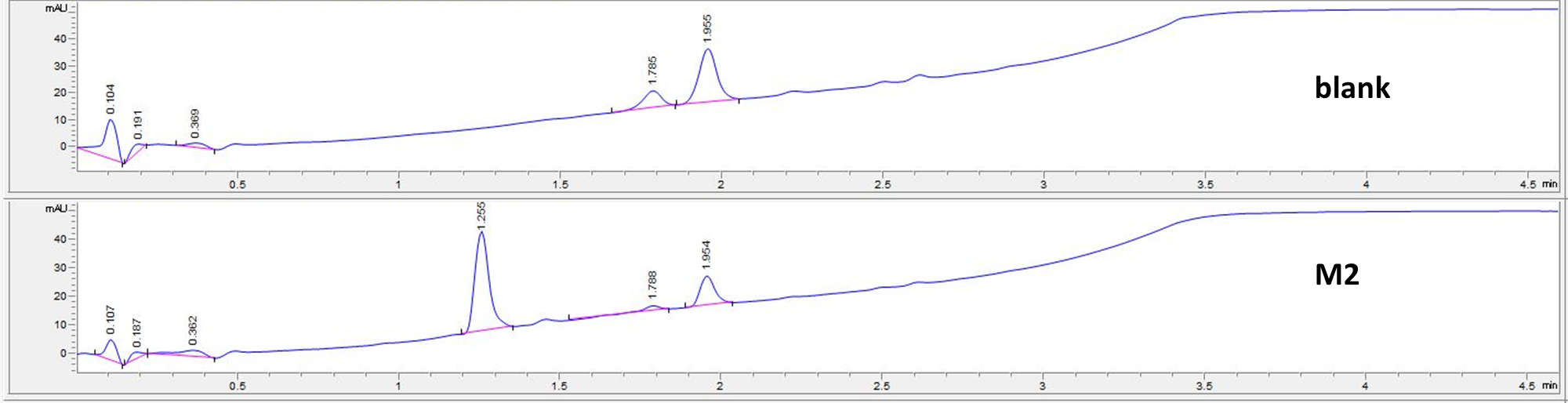


97% calculated purity by HPLC.

//nmrxiv.org/project/zHJDzpEvl4KhtWqYMyFd63edQc3poLvawWErPIcA


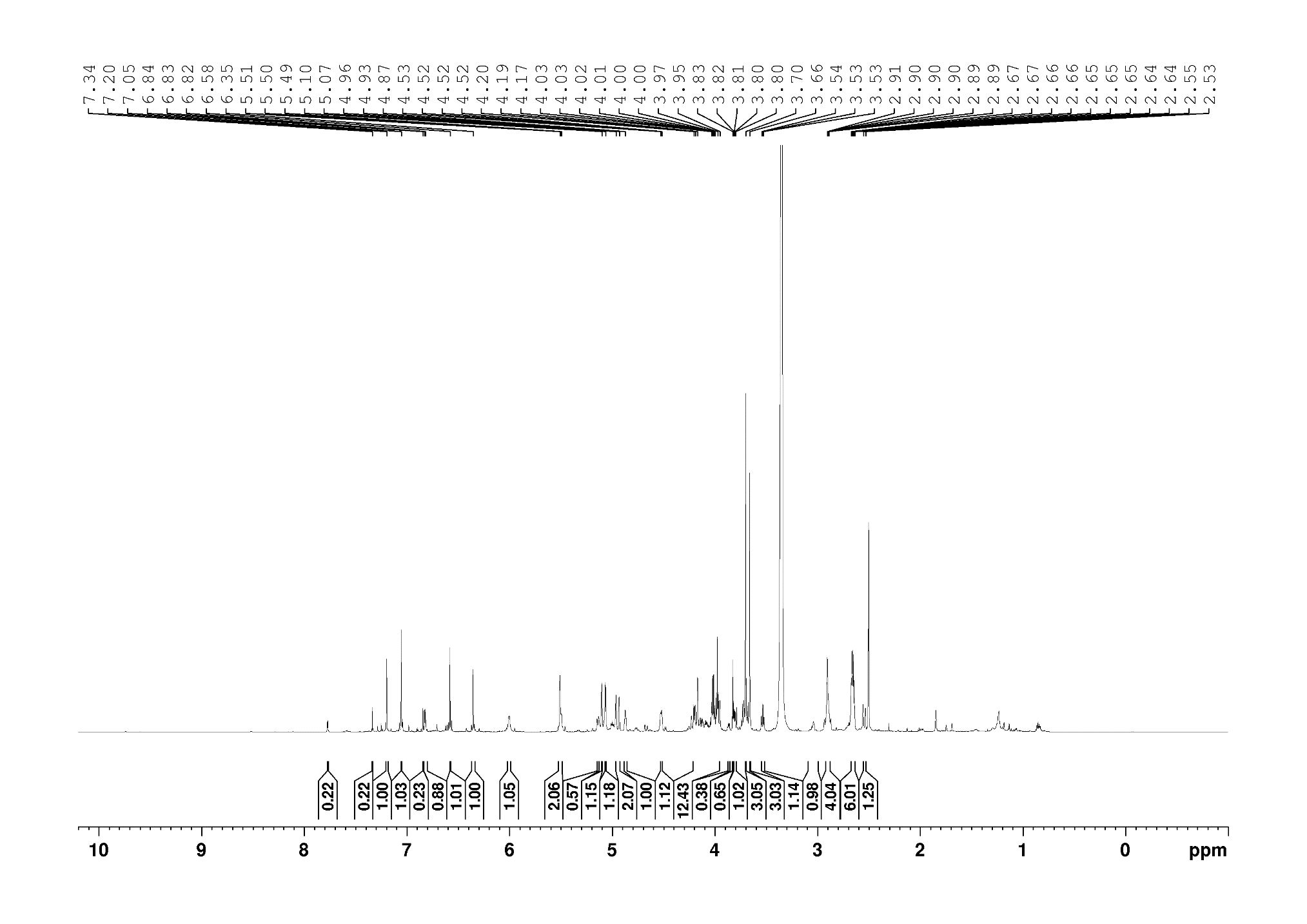


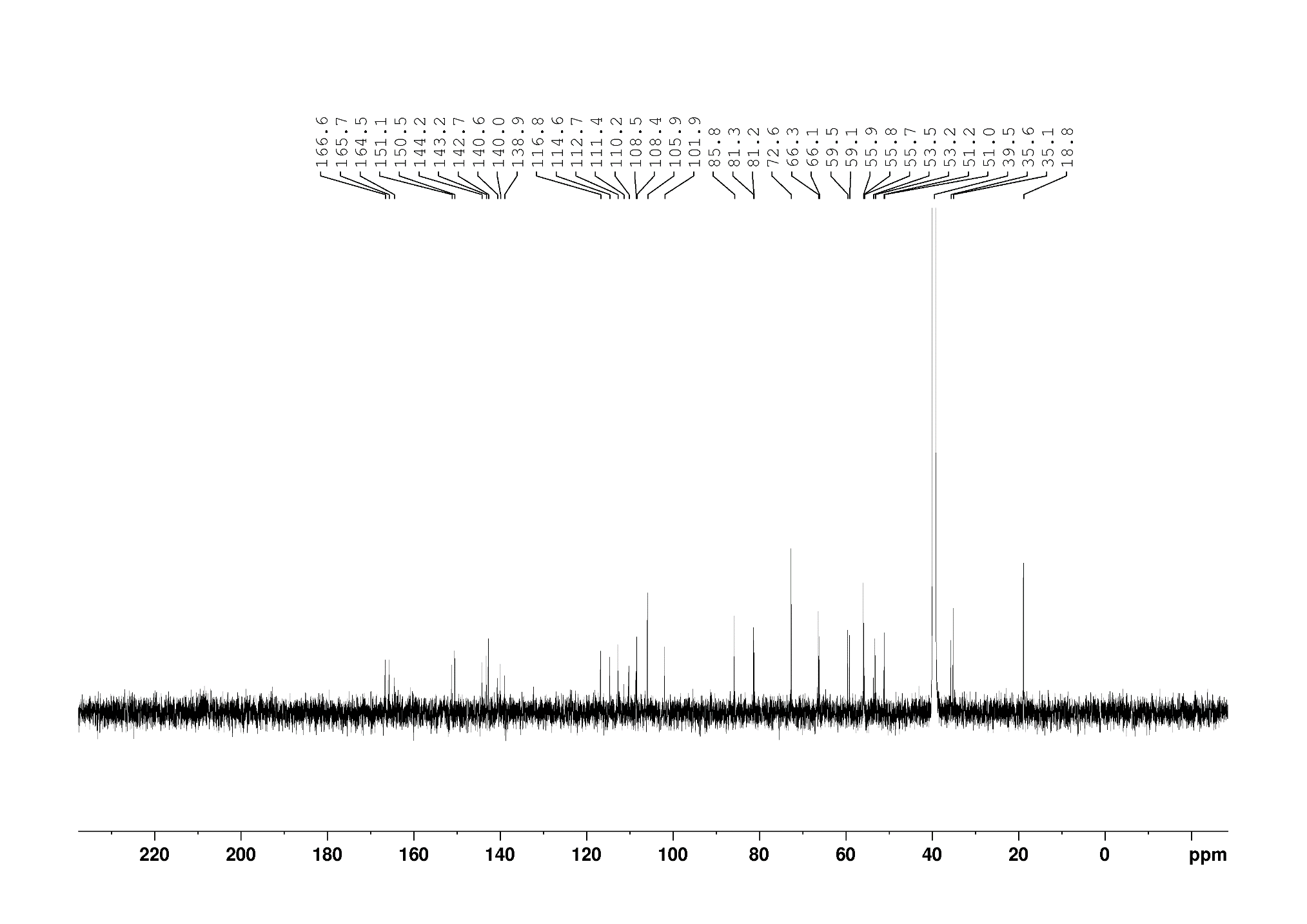


**8,8-dibromobicyclo[5.1.0]octane (16)**


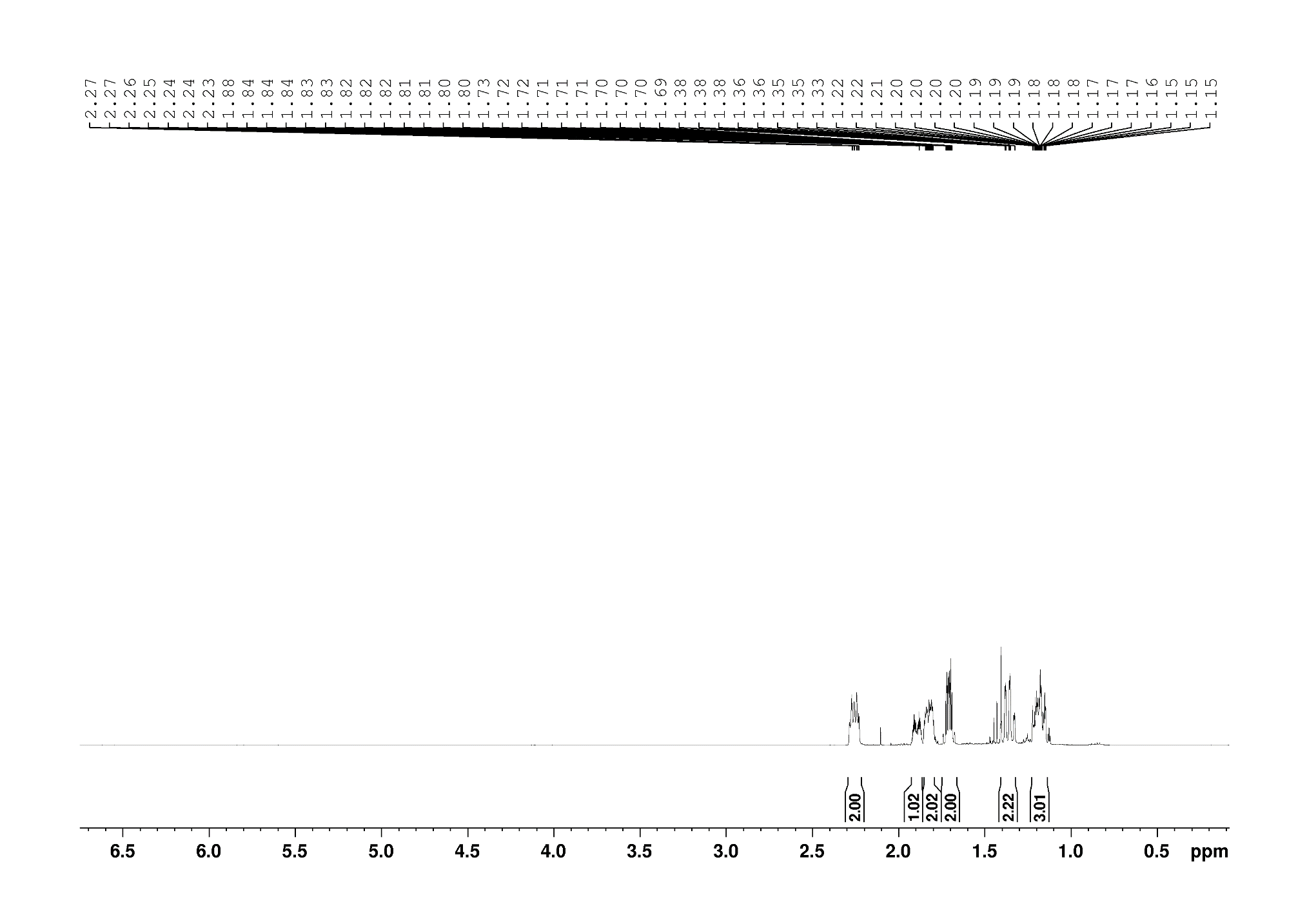


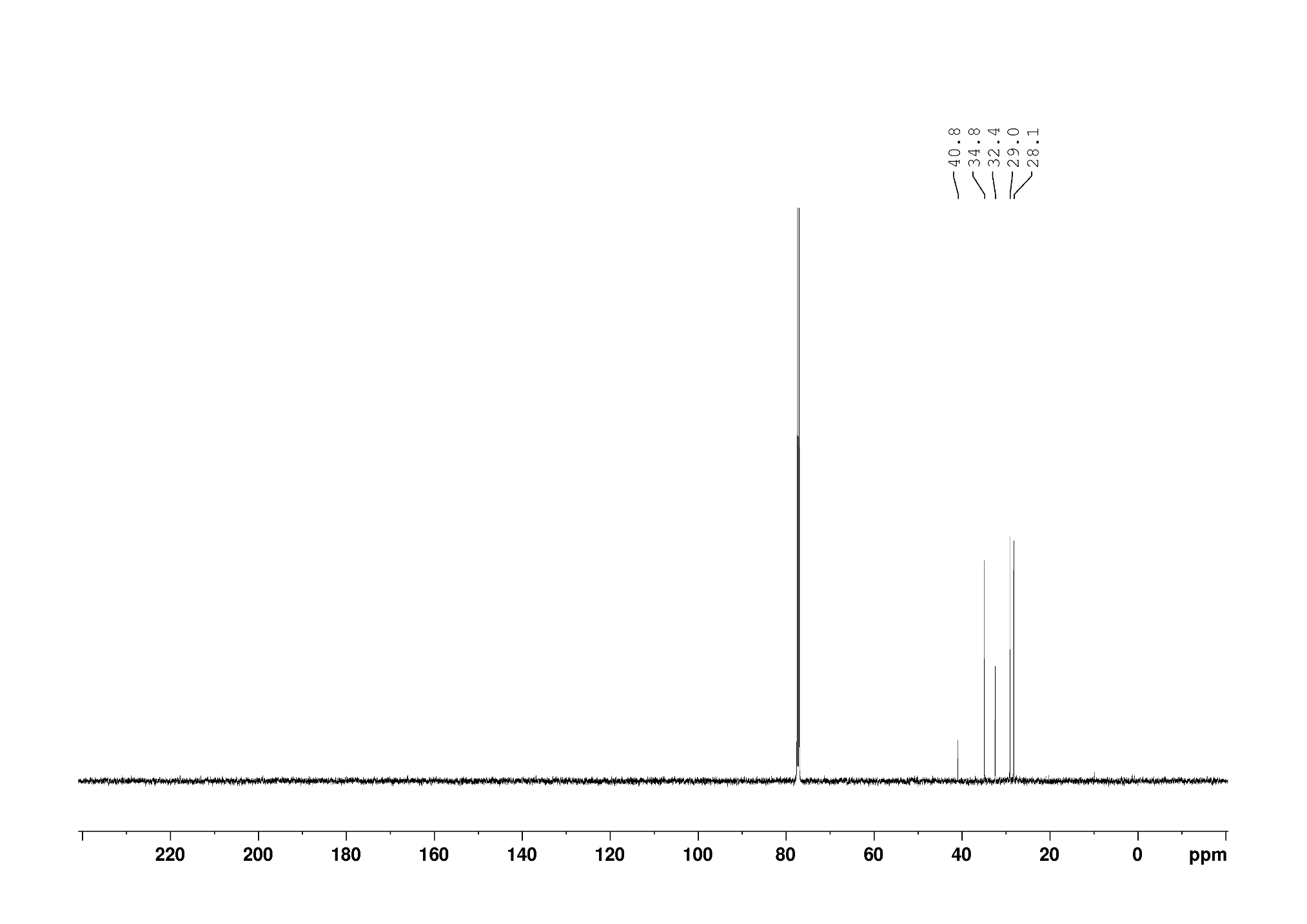


**2-(cyclooct-2-yn-1-yloxy)ethan-1-ol (18)**


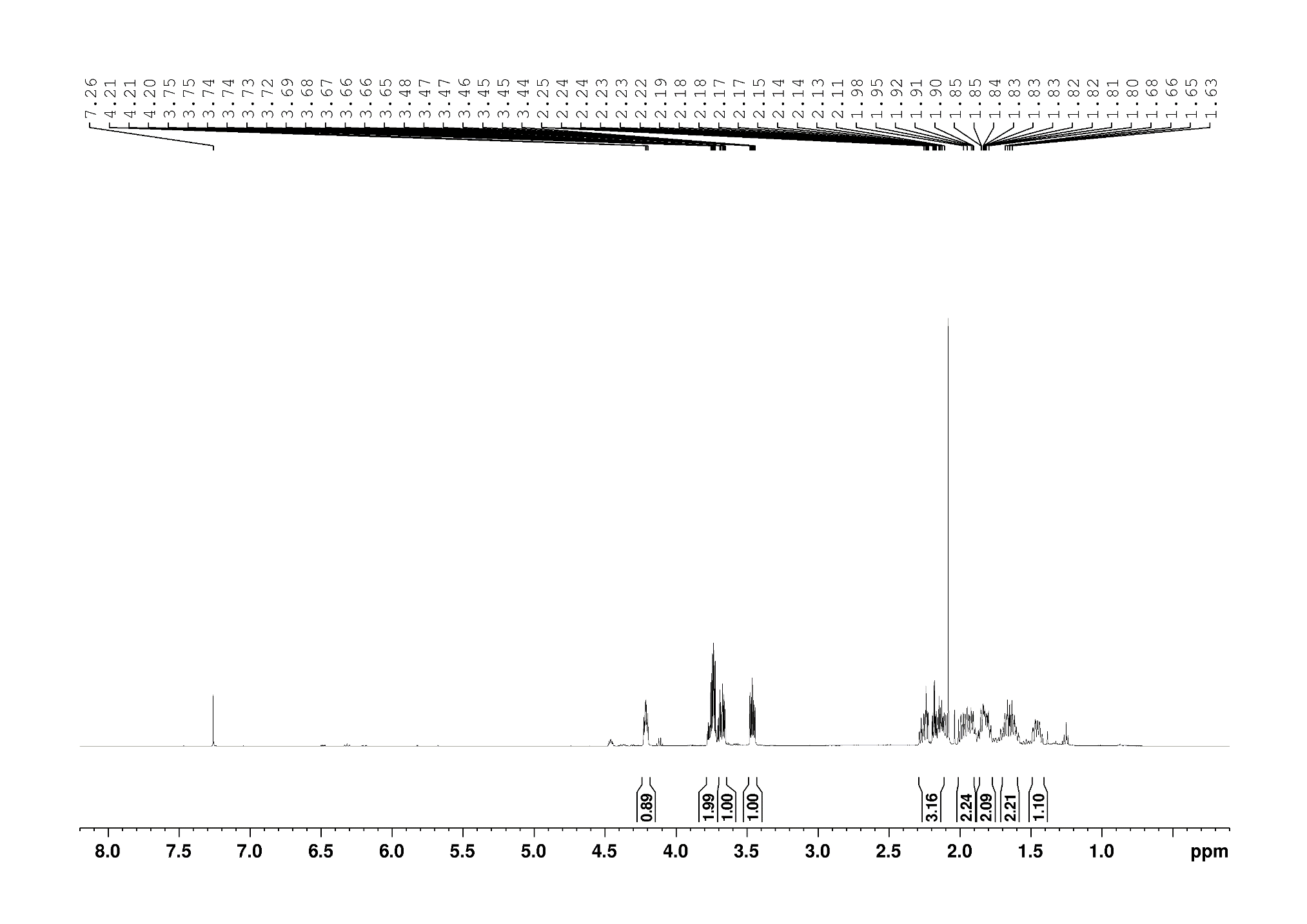


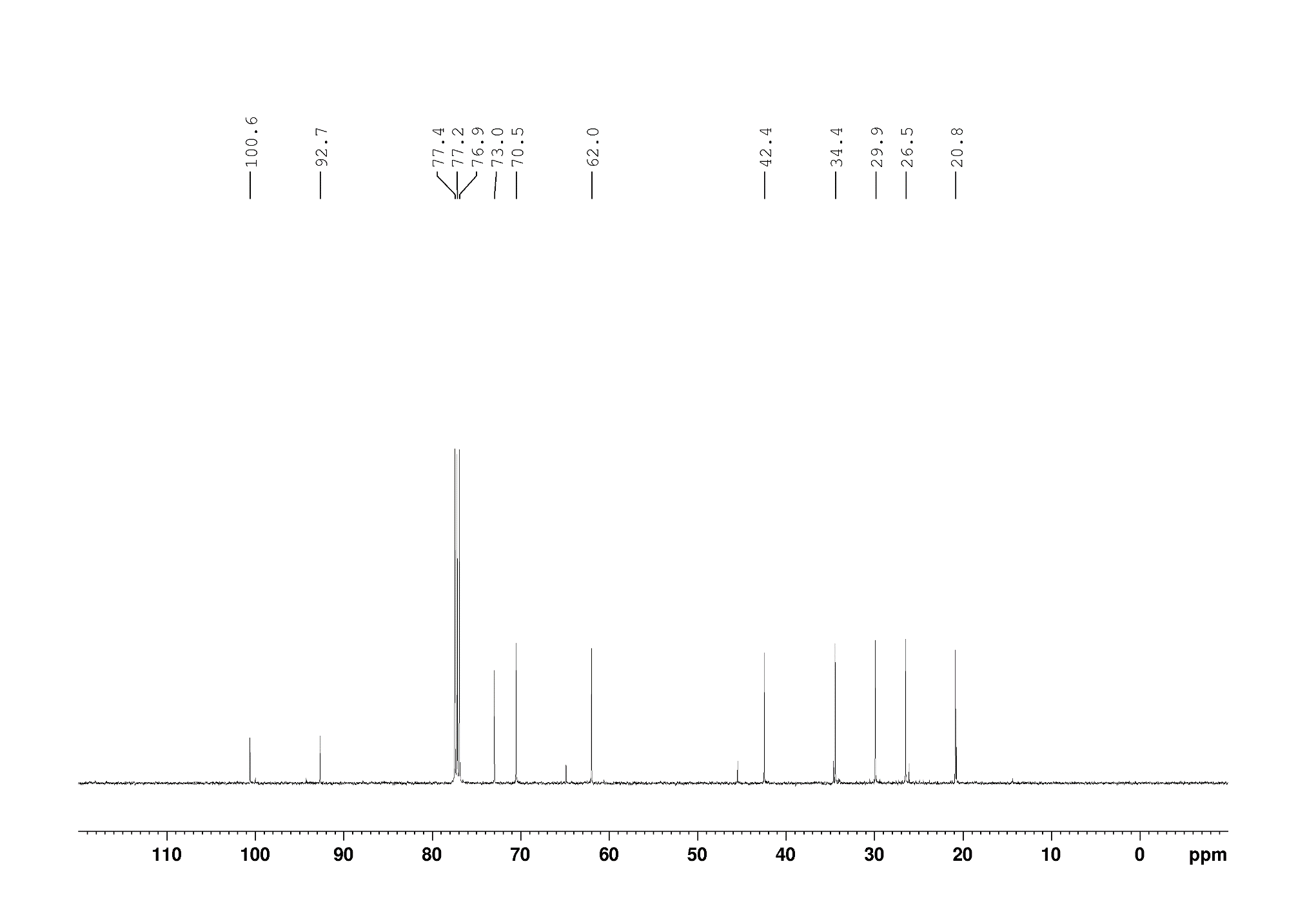


**2-(cyclooct-2-yn-1-yloxy)ethyl 4-methylbenzenesulfonate (19)**


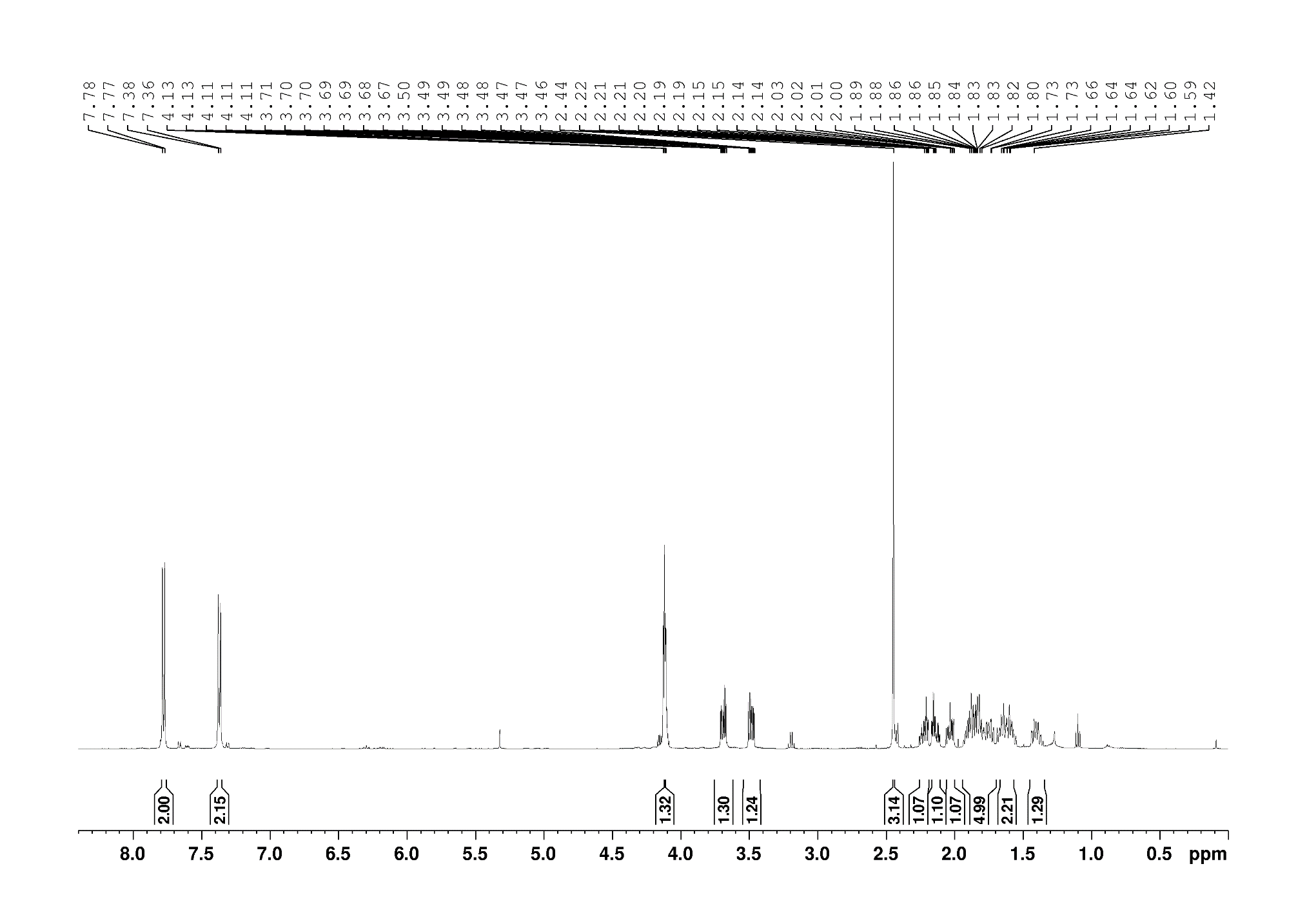


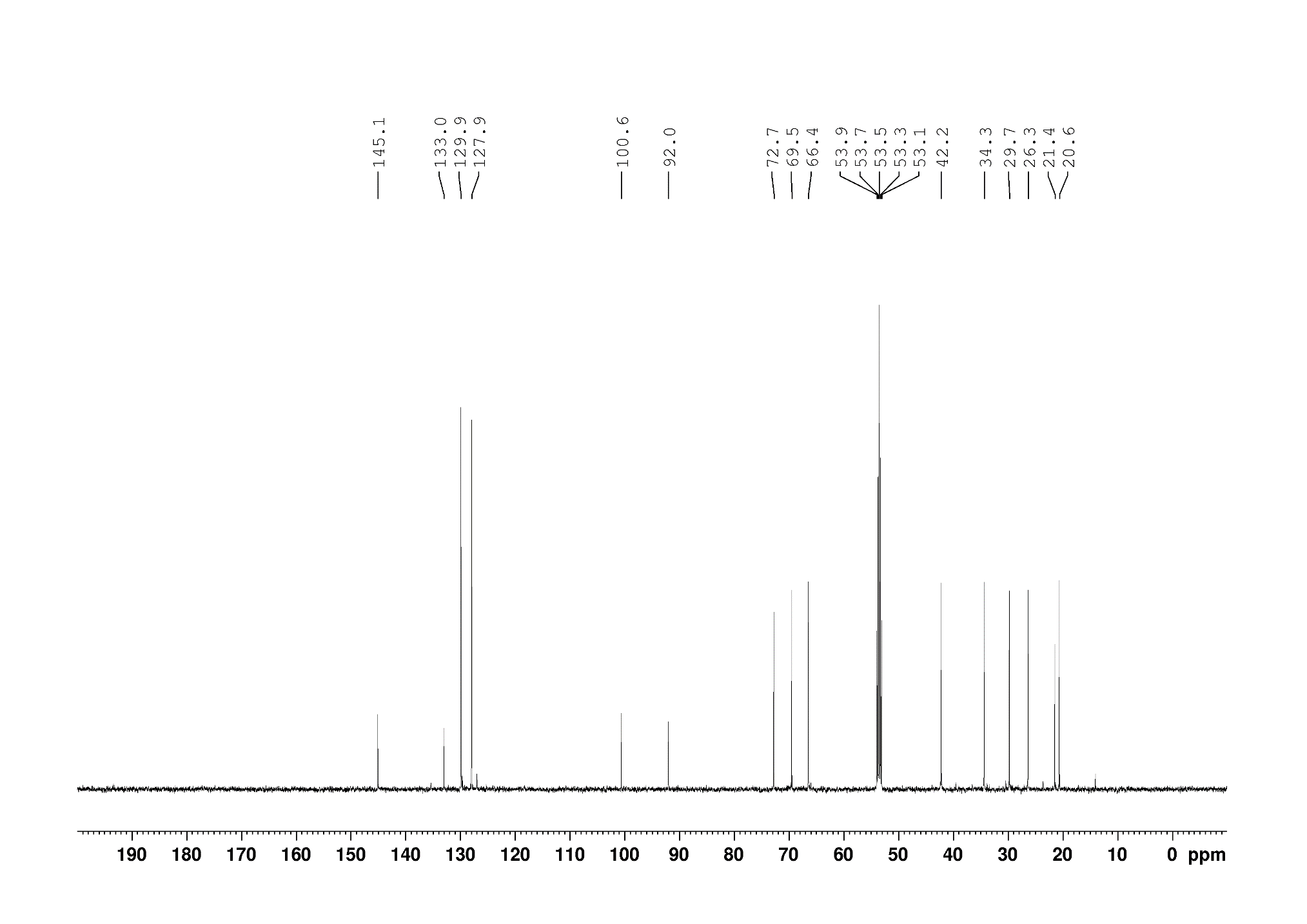


**(11a*S*)-8-(2-(cyclooct-2-yn-1-yloxy)ethoxy)-7-methoxy-2-methylene-1,2,3,11a-tetrahydro-5H-benzo[e]pyrrolo[1,2-a][1,4]diazepin-5-one (M3)**


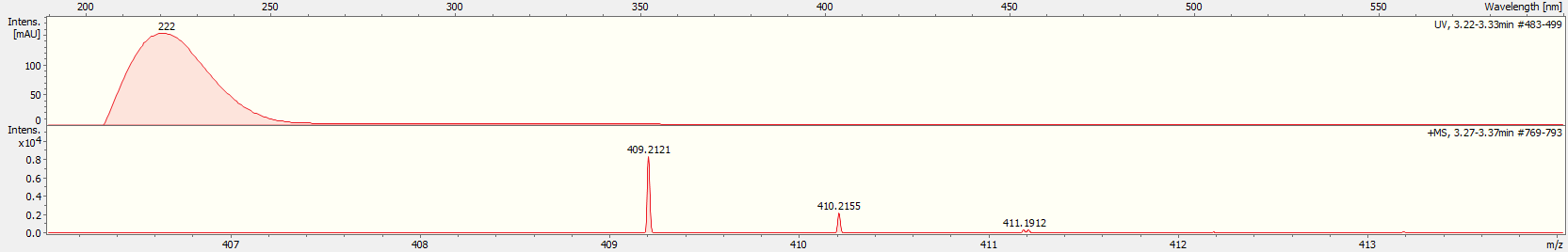


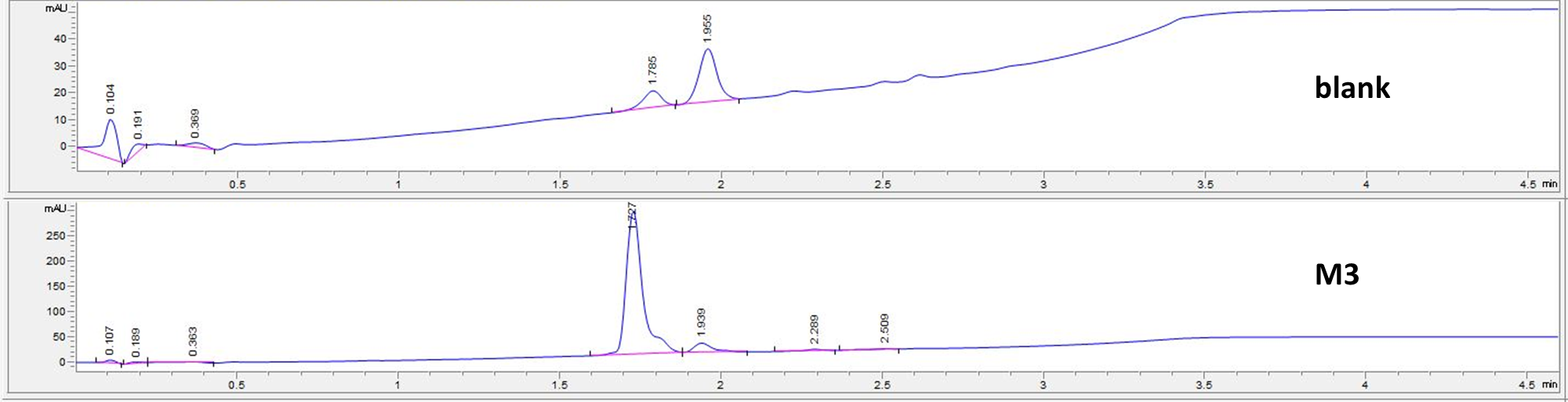


98% calculated purity by HPLC.

//nmrxiv.org/project/zHJDzpEvl4KhtWqYMyFd63edQc3poLvawWErPIcA


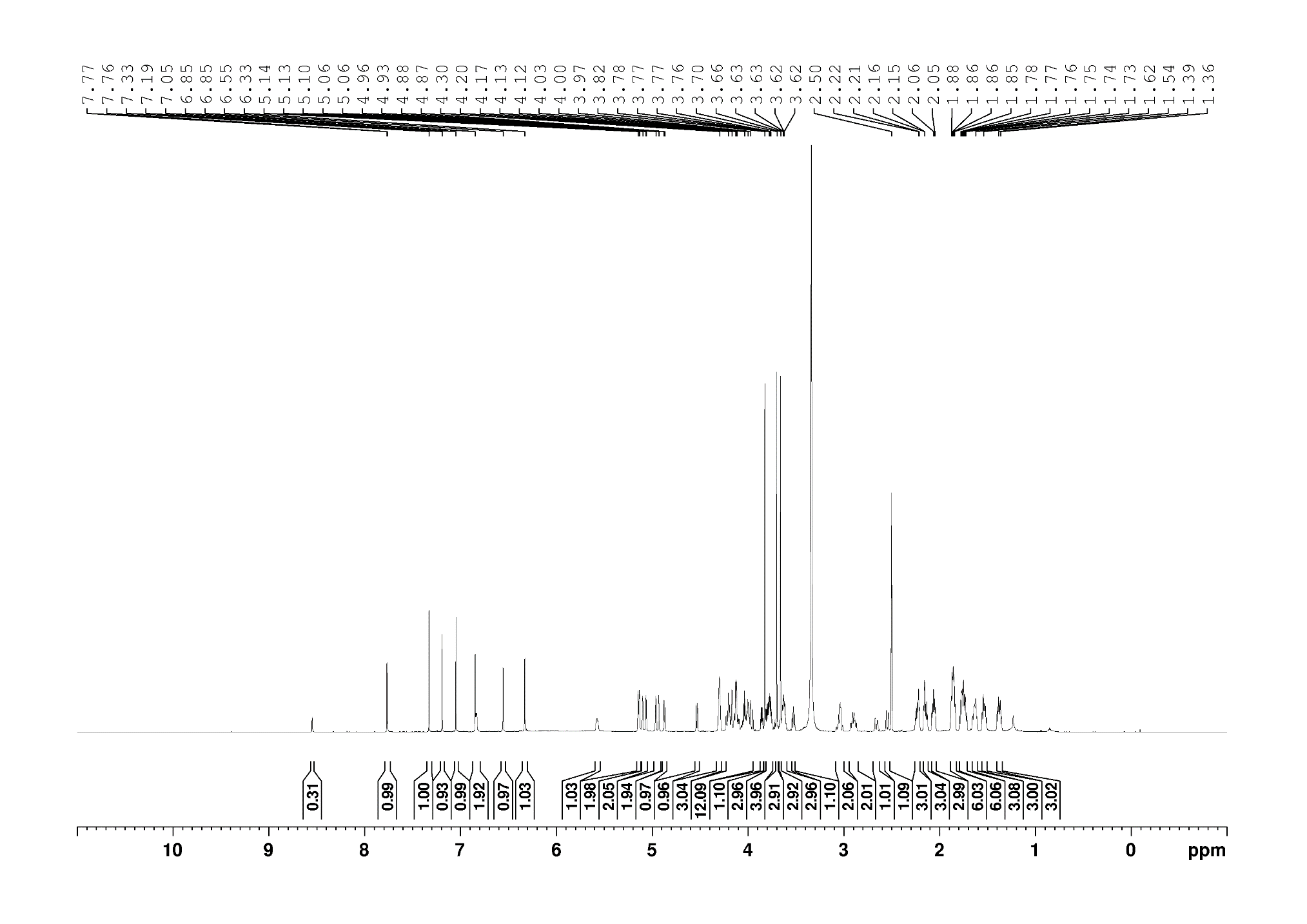


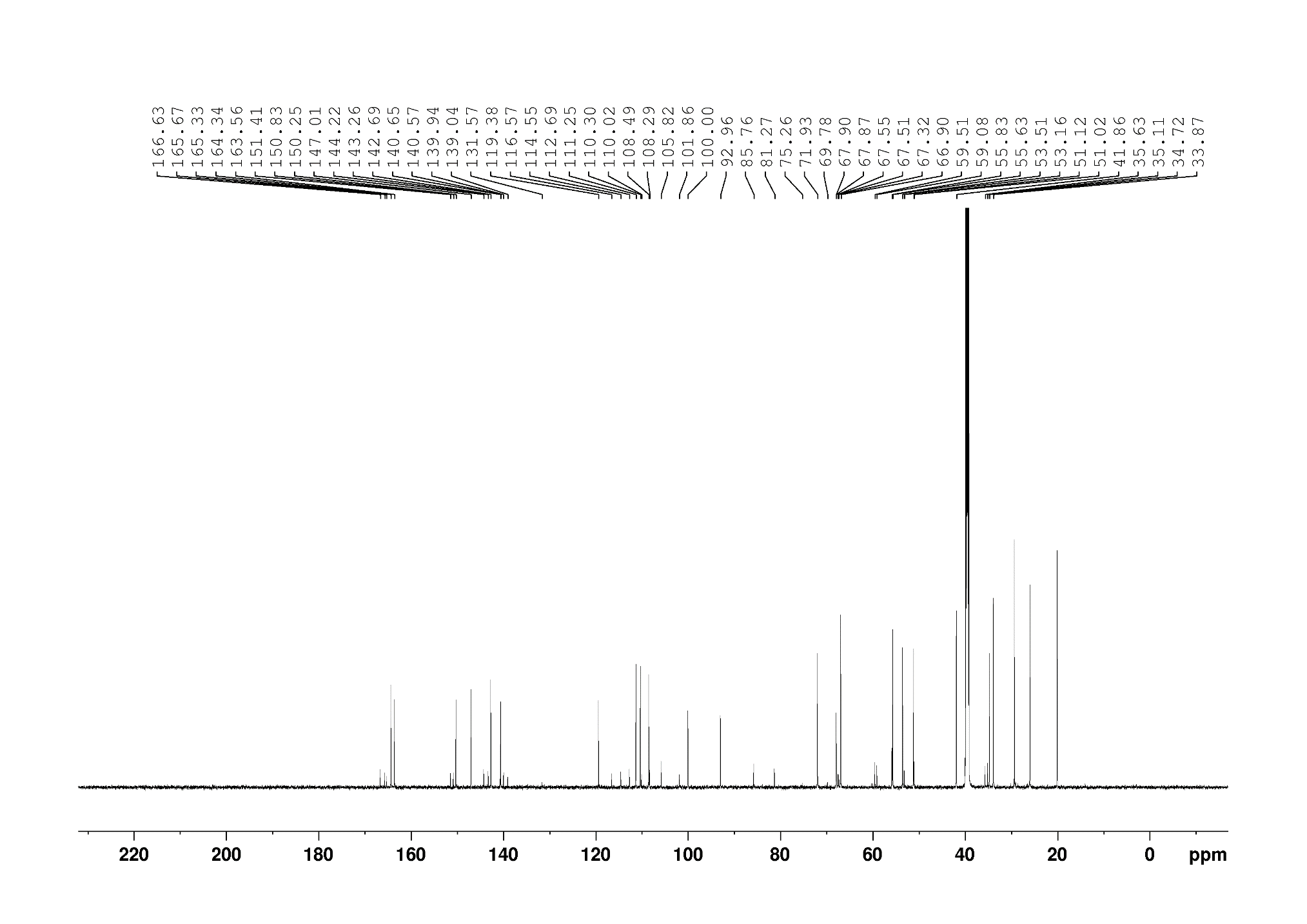


**bicyclo[6.1.0]non-4-yn-9-ylmethyl (4-nitrophenyl) carbonate (21)**


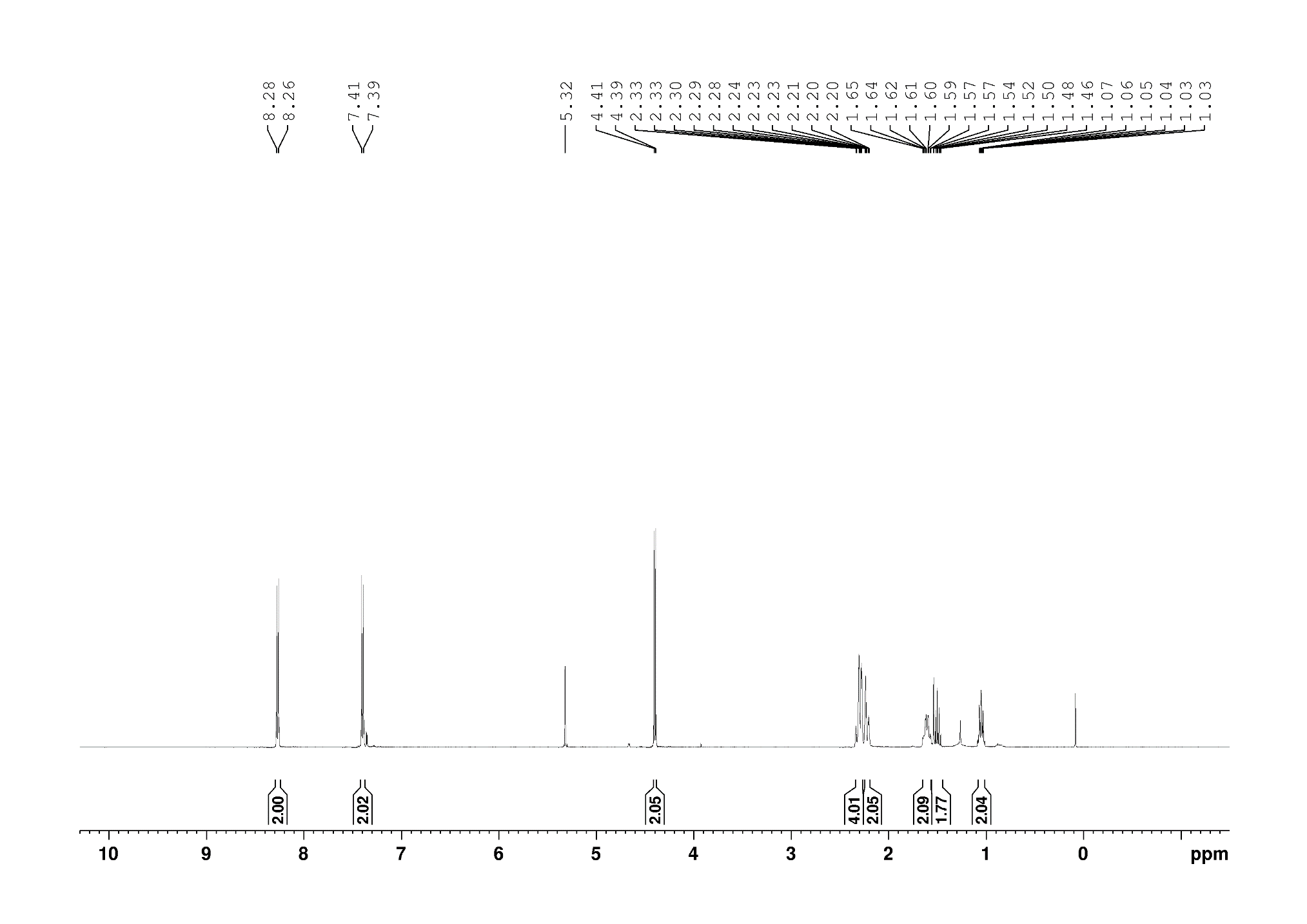


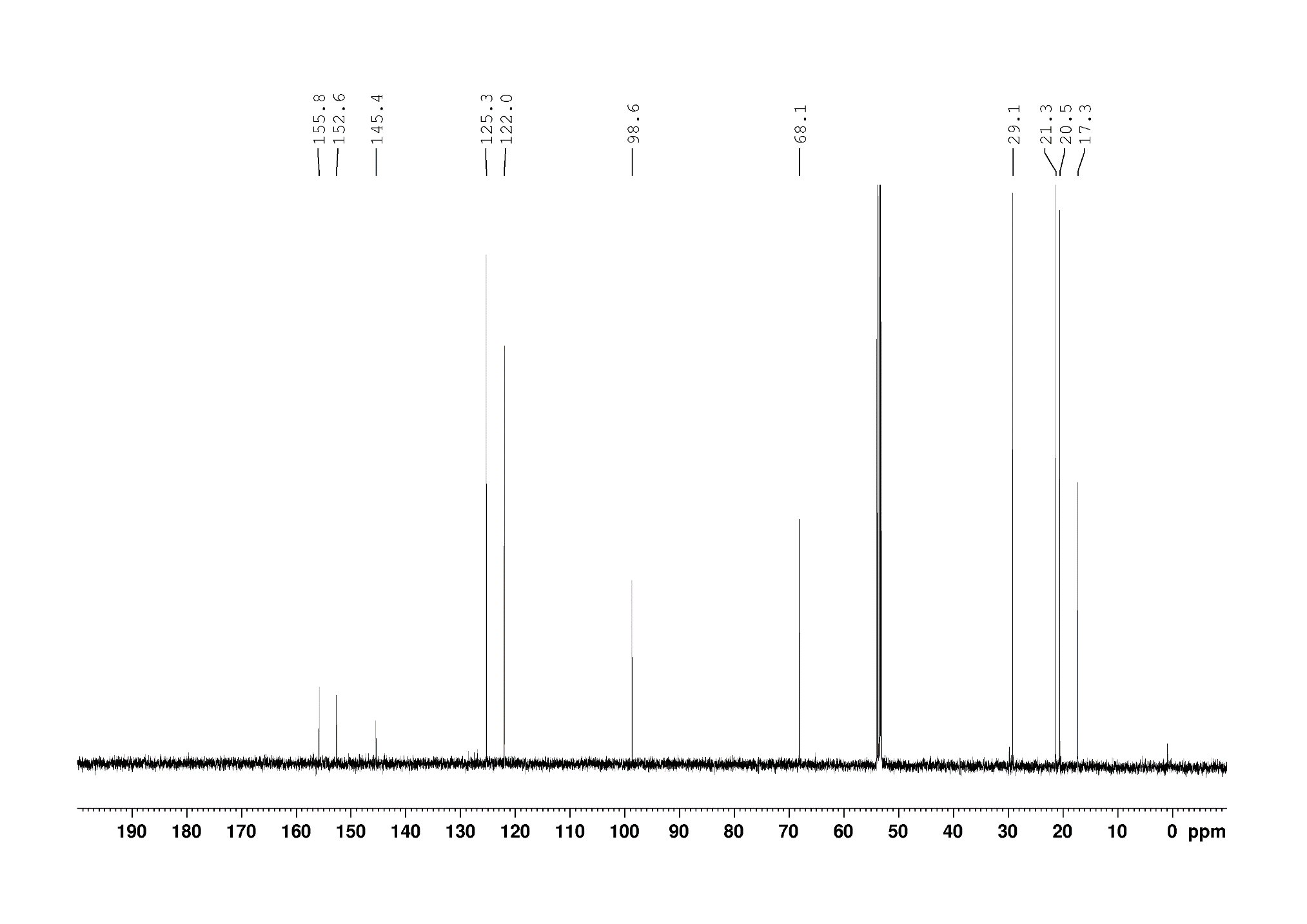


**bicyclo[6.1.0]non-4-yn-9-ylmethyl((*S*)-7-methoxy-2-methylene-5-oxo-2,3,5,11a-tetrahydro-1H-benzo[e]pyrrolo[1,2-a][1,4]diazepin-8-yl) carbonate (M4)**


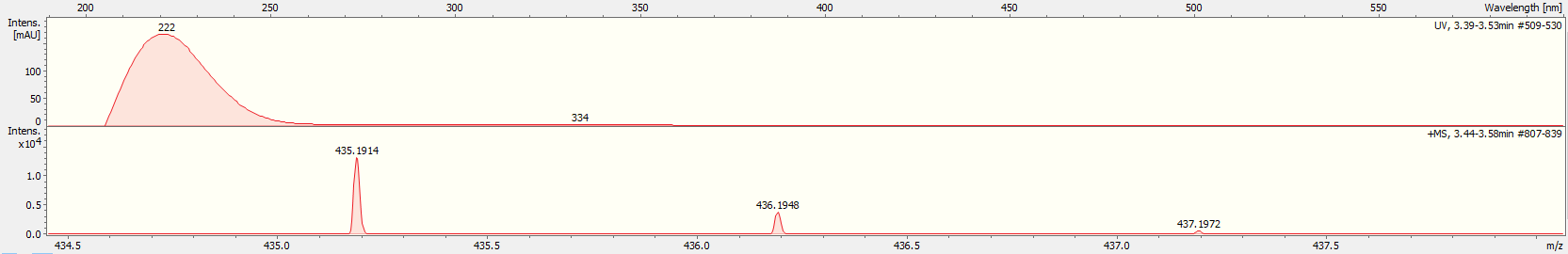


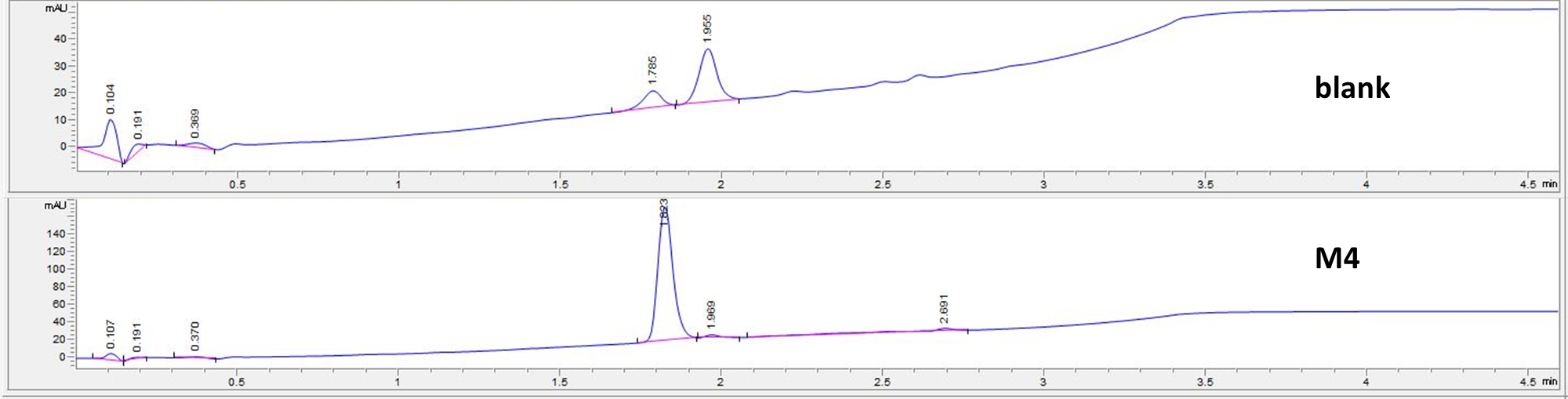


95% calculated purity by HPLC.

//nmrxiv.org/project/zHJDzpEvl4KhtWqYMyFd63edQc3poLvawWErPIcA


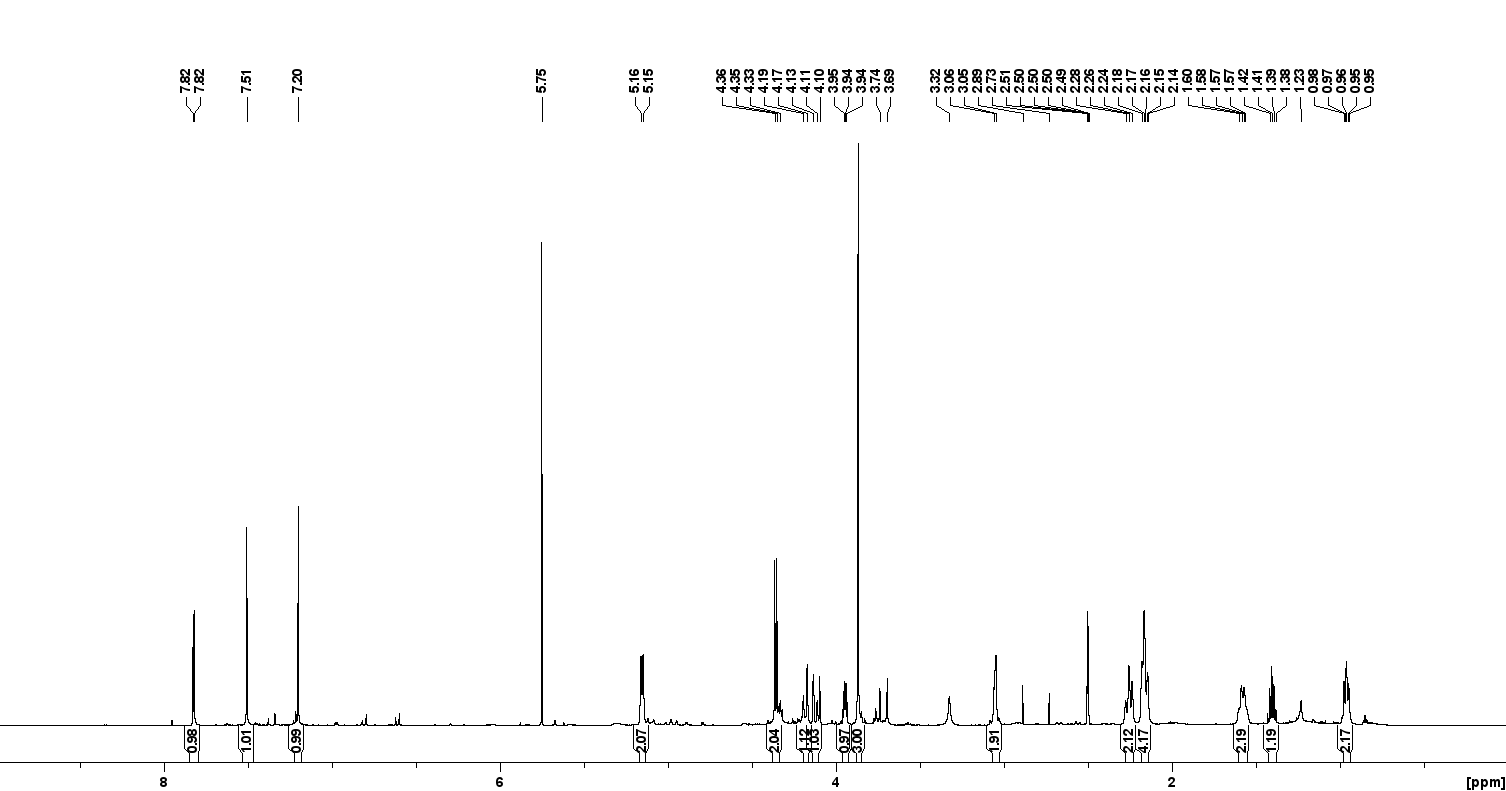


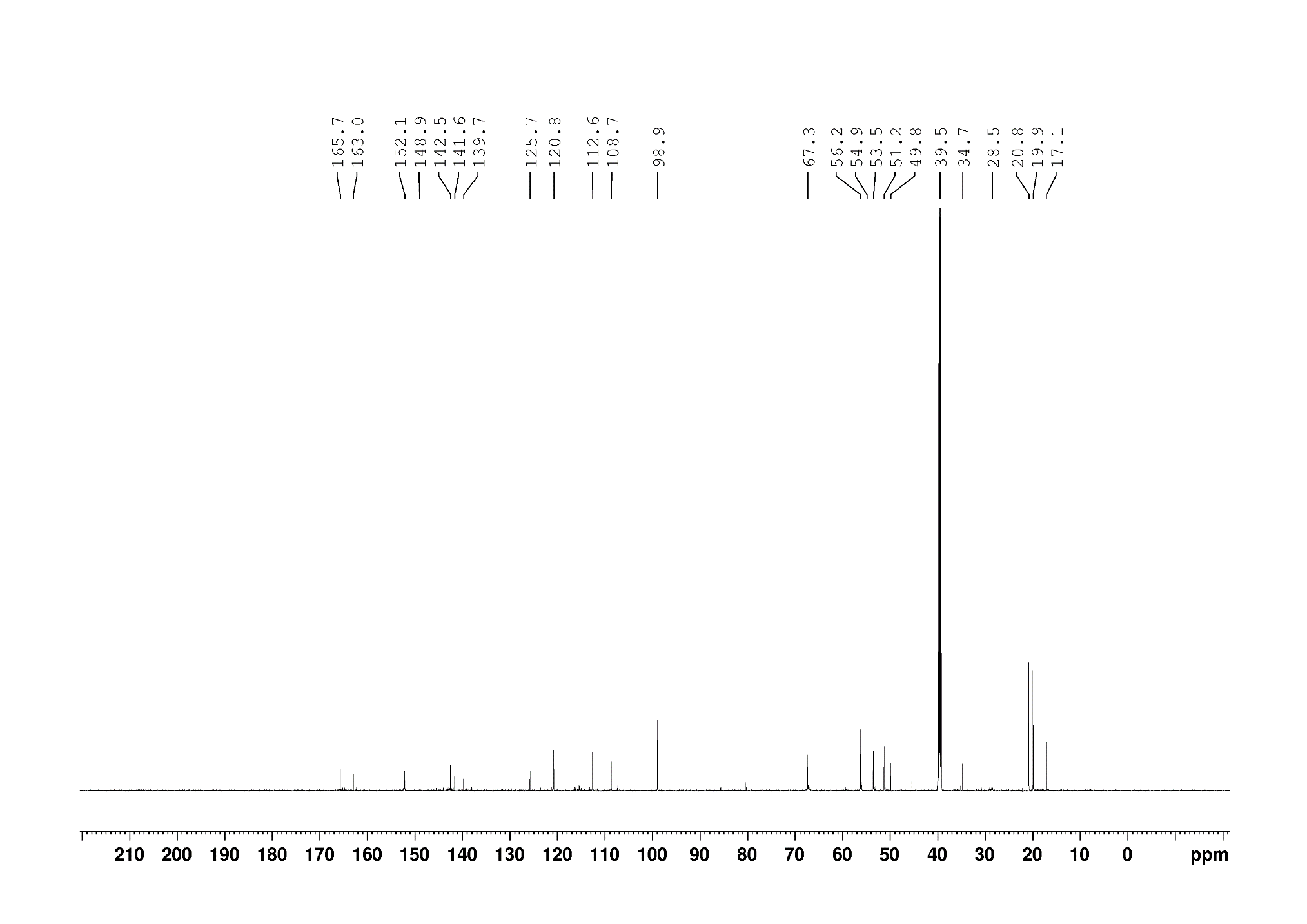


**(*S*)-7-methoxy-8-((1-(2-(((*S*)-7-methoxy-2-methylene-5-oxo-2,3,5,11a-tetrahydro-1H-benzo[e]pyrrolo[1,2-a][1,4]diazepin-8-yl)oxy)ethyl)-1H-1,2,3-triazol-4-yl)methoxy)-2-methylene-1,2,3,11a-tetrahydro-5H-benzo[e]pyrrolo[1,2-a][1,4]diazepin-5-one (D1)**


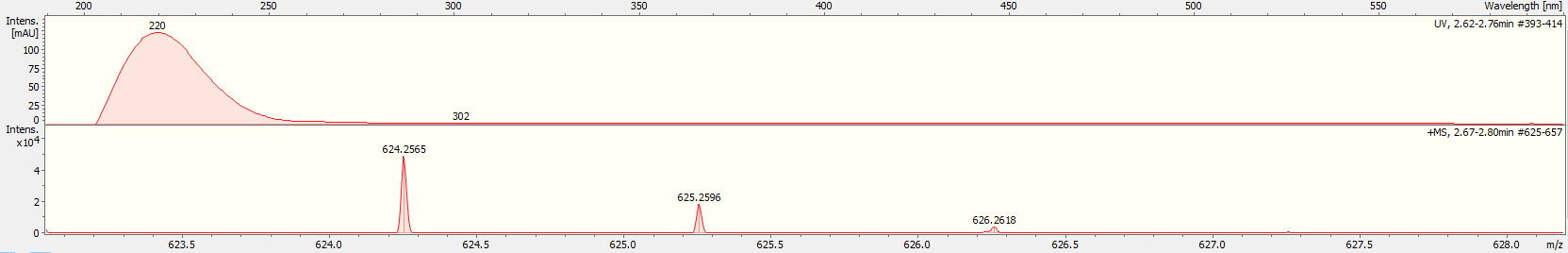


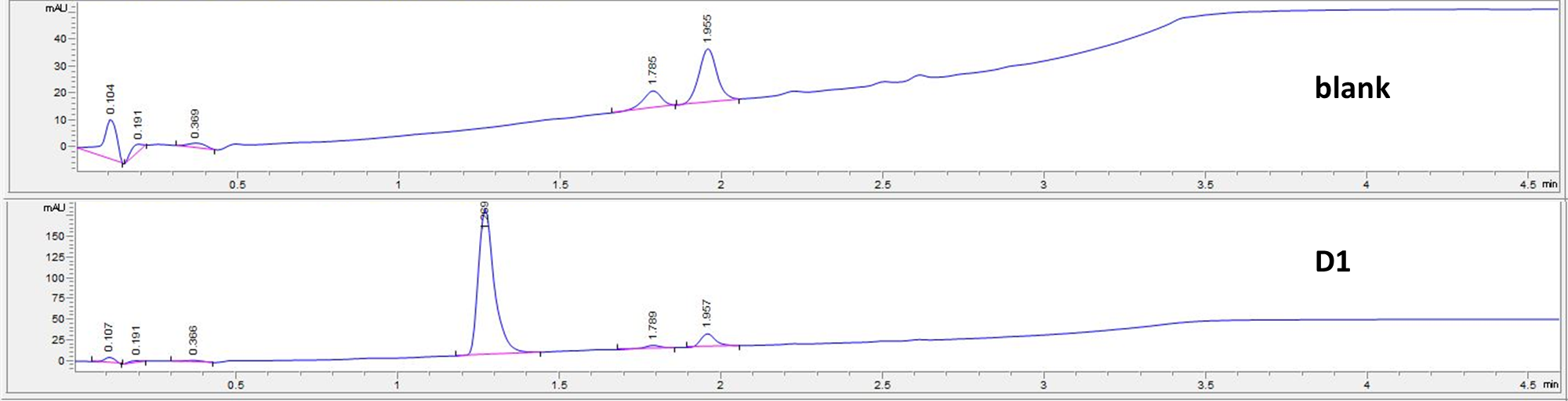


100% calculated purity by HPLC.

//nmrxiv.org/project/zHJDzpEvl4KhtWqYMyFd63edQc3poLvawWErPIcA


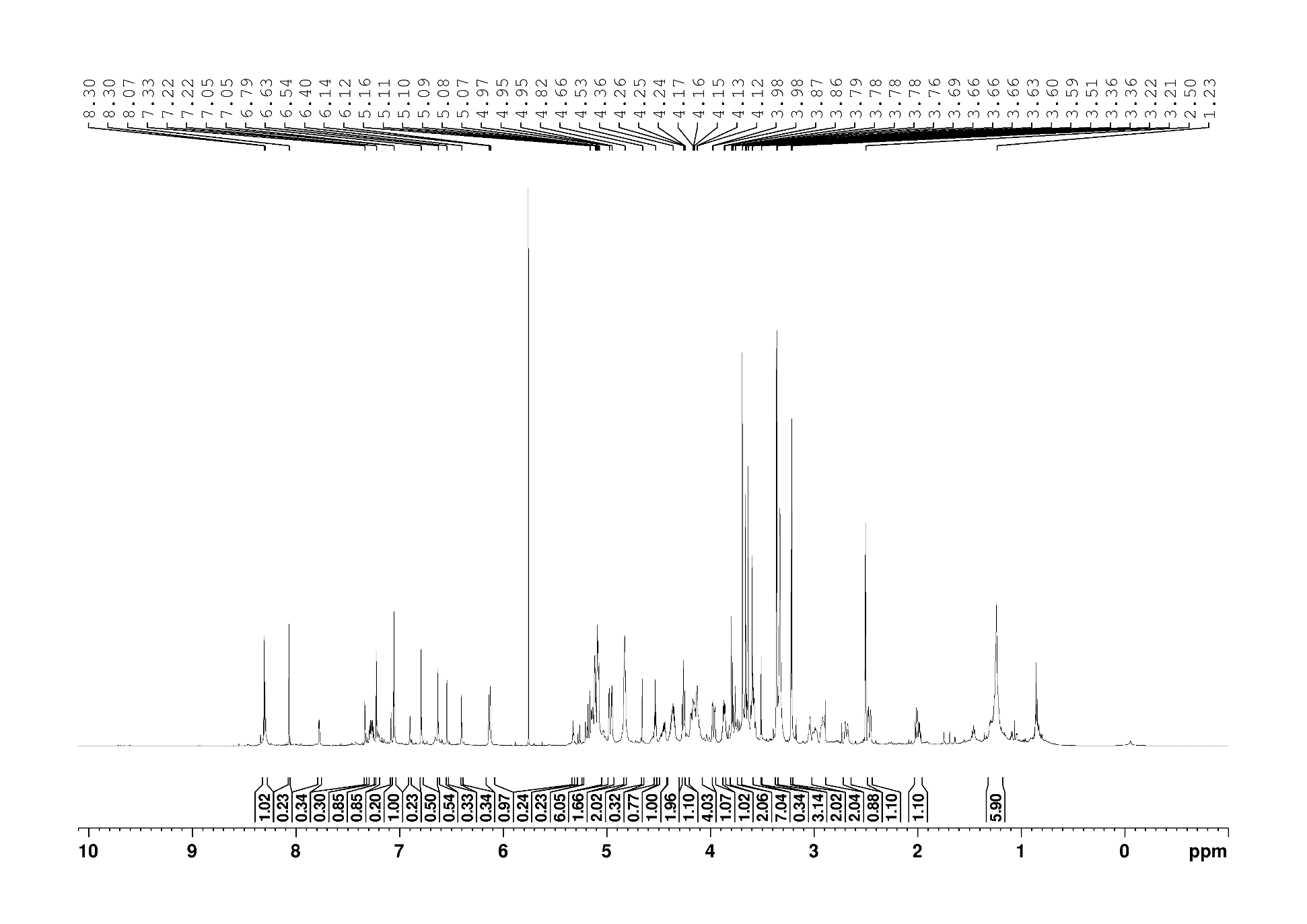


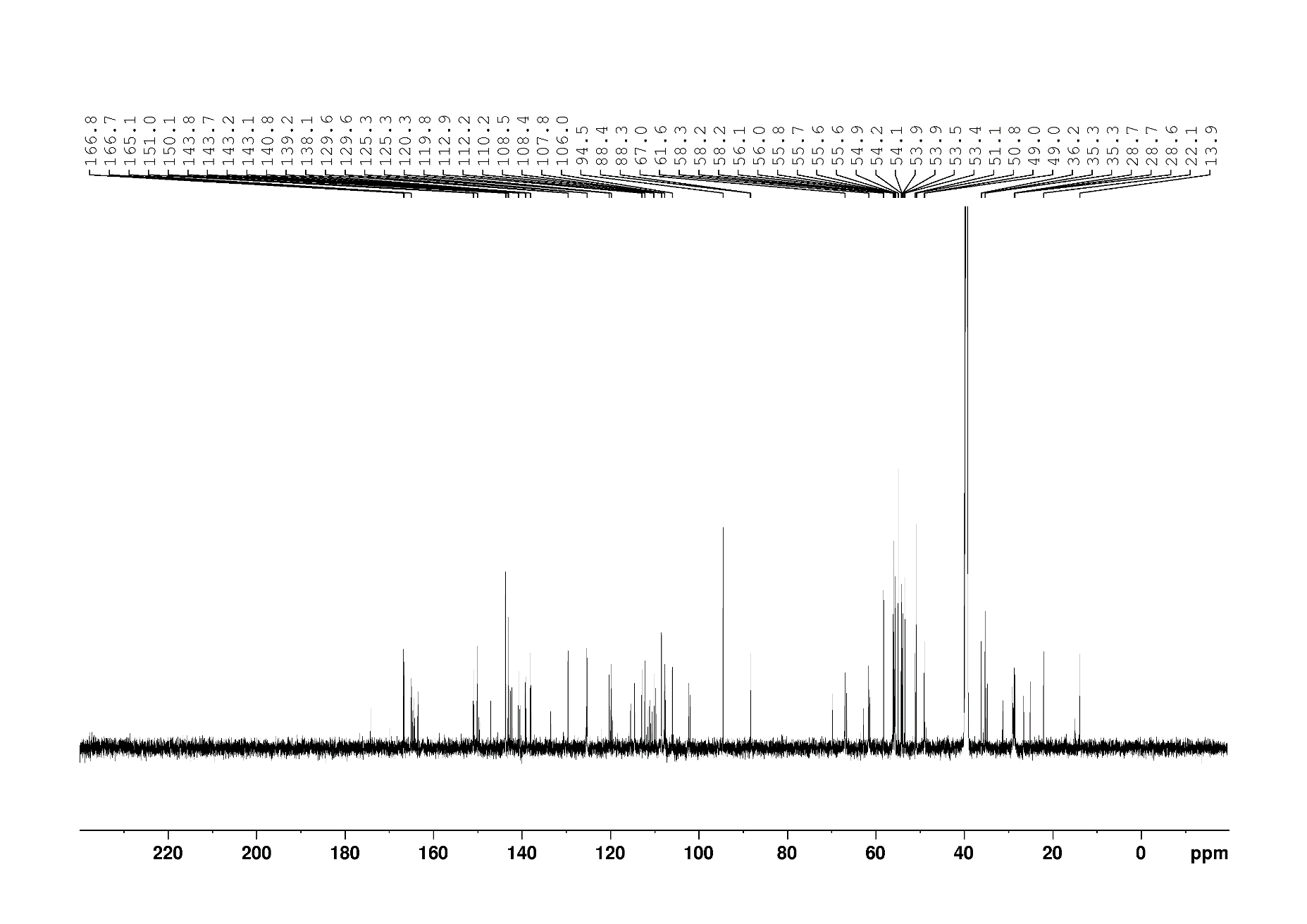


**(11a*S*,11a'*S*)-8,8'-(((1H-1,2,3-triazole-1,4-diyl)bis(ethane-2,1-diyl))bis(oxy))bis(7-methoxy-2-methylene-1,2,3,11a-tetrahydro-5H-benzo[e]pyrrolo[1,2-a][1,4]diazepin-5-one) (D2)**


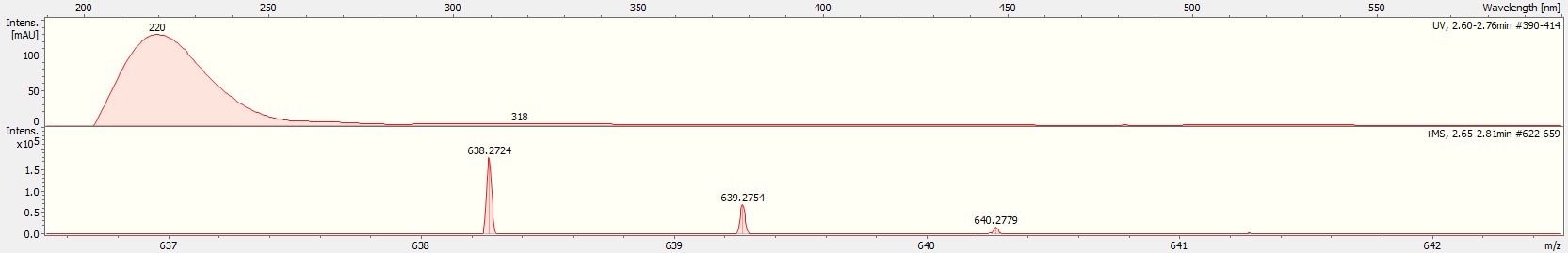


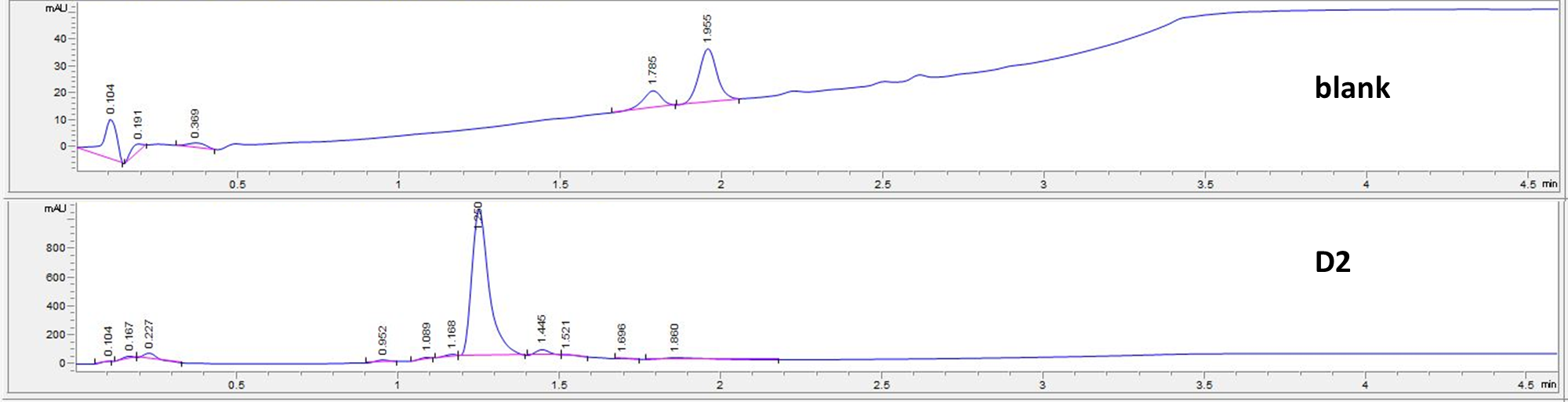


86% calculated purity by HPLC.

//nmrxiv.org/project/zHJDzpEvl4KhtWqYMyFd63edQc3poLvawWErPIcA


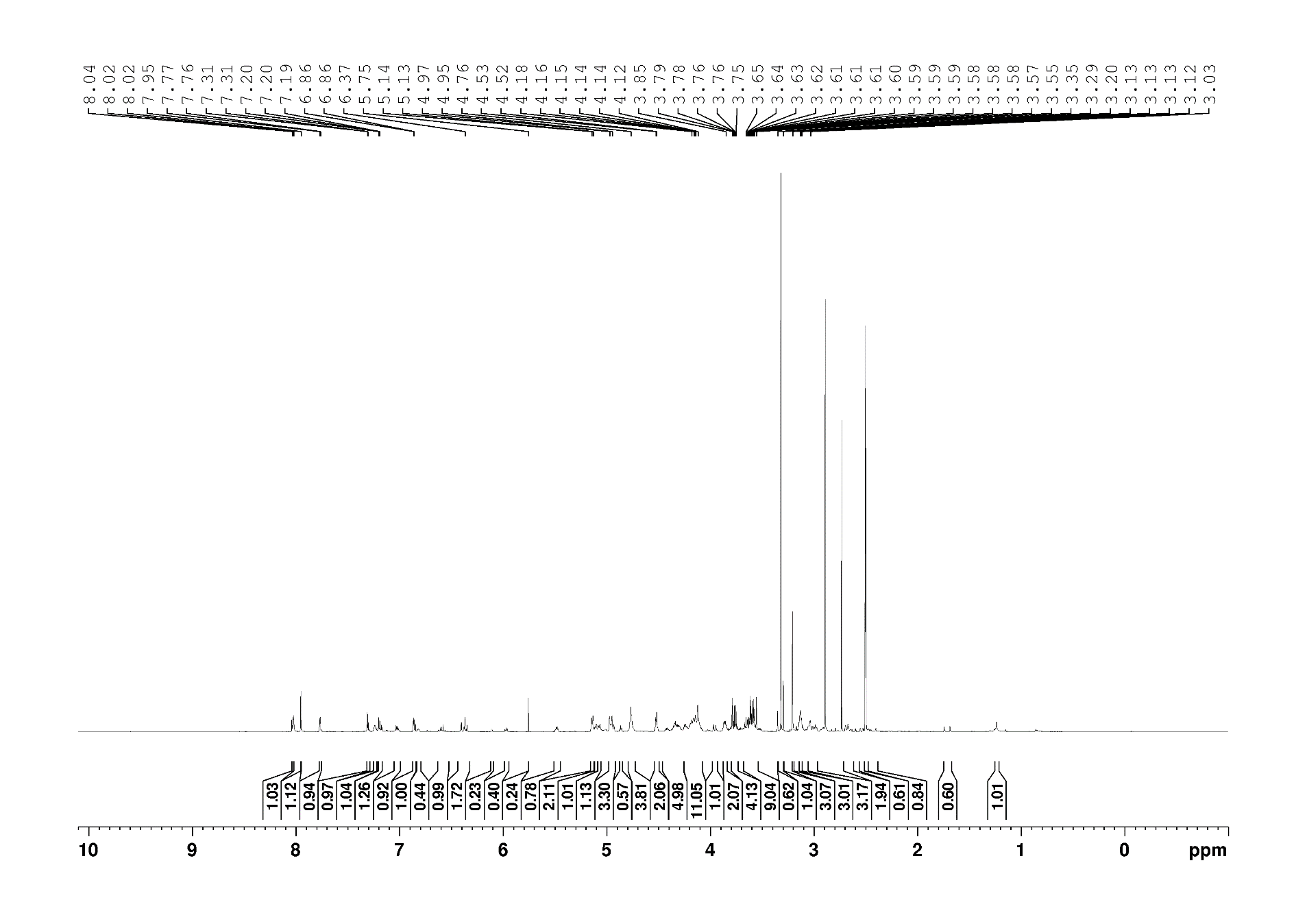


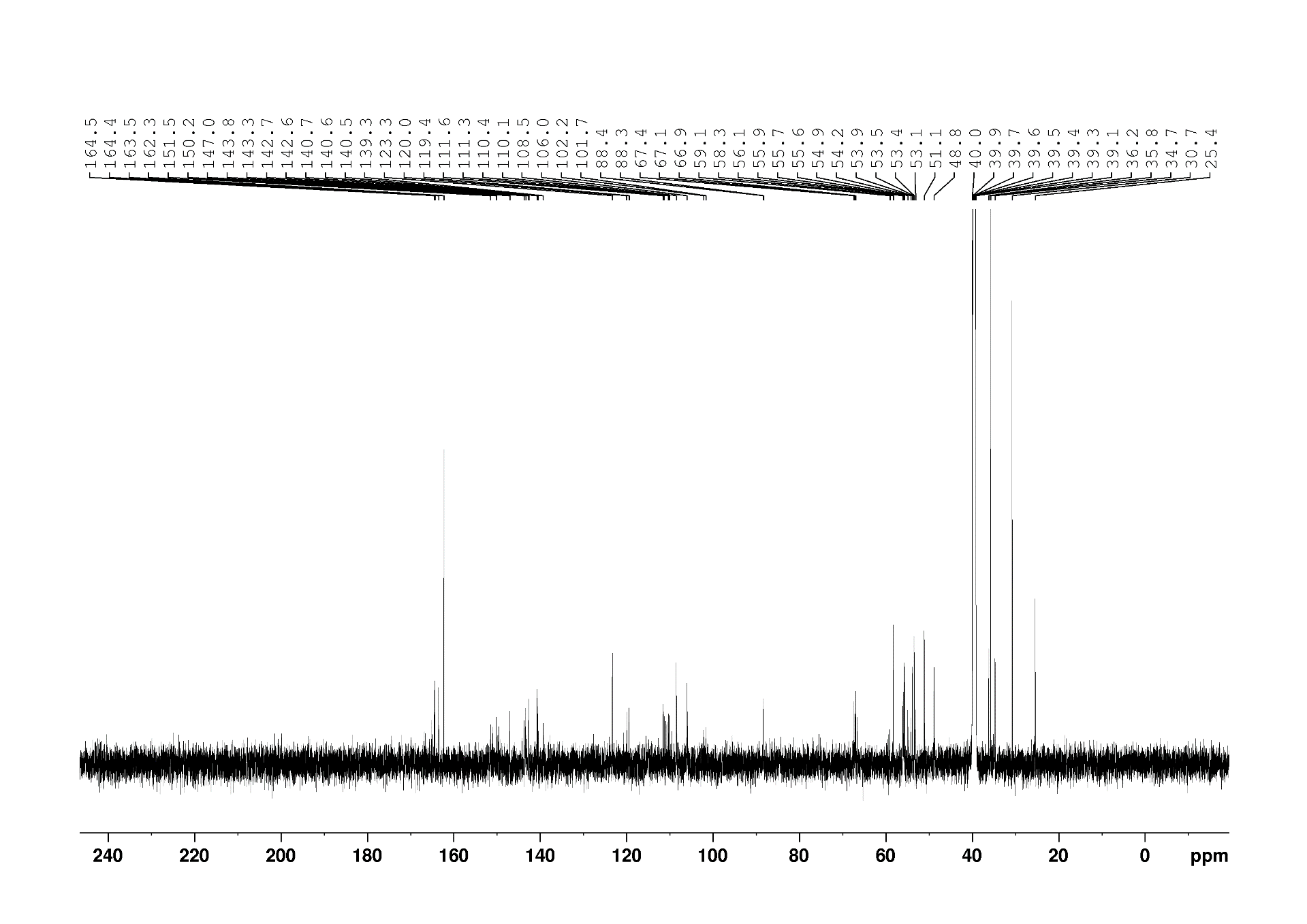


**(11a*S*)-7-methoxy-8-(2-(4-(2-(((*S*)-7-methoxy-2-methylene-5-oxo-2,3,5,11a-tetrahydro-1H-benzo[e]pyrrolo[1,2-a][1,4]diazepin-8-yl)oxy)ethoxy)-4,5,6,7,8,9-hexahydro-1H-cycloocta[d][1,2,3]triazol-1-yl)ethoxy)-2-methylene-1,2,3,11a-tetrahydro-5H-benzo[e]pyrrolo[1,2-a][1,4]diazepin-5-one (D3)**


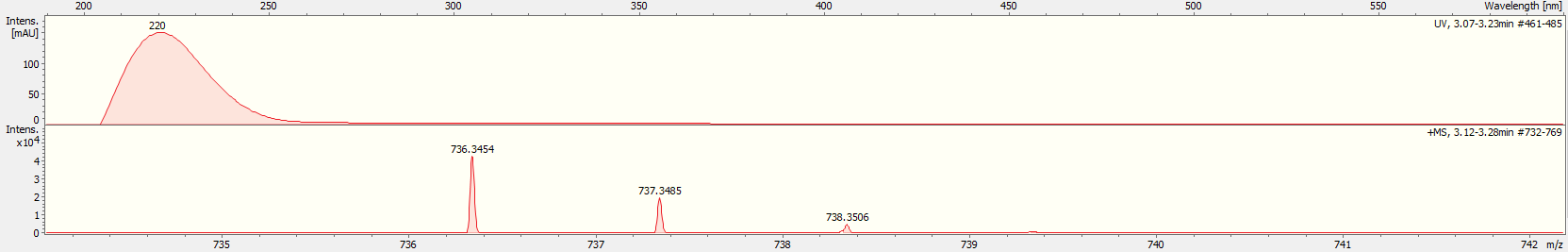


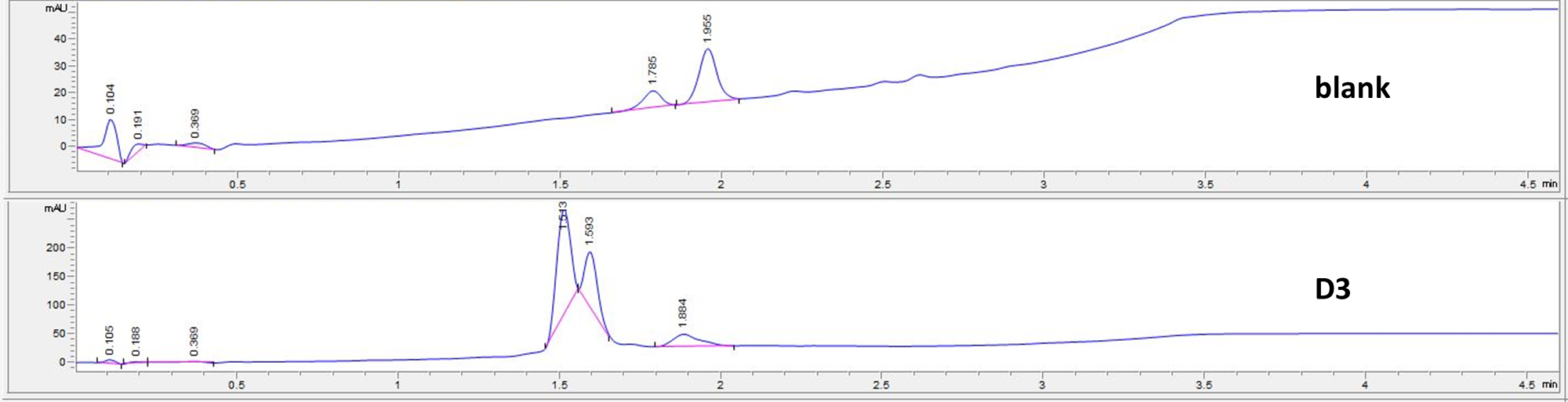


92% calculated purity by HPLC.

//nmrxiv.org/project/zHJDzpEvl4KhtWqYMyFd63edQc3poLvawWErPIcA


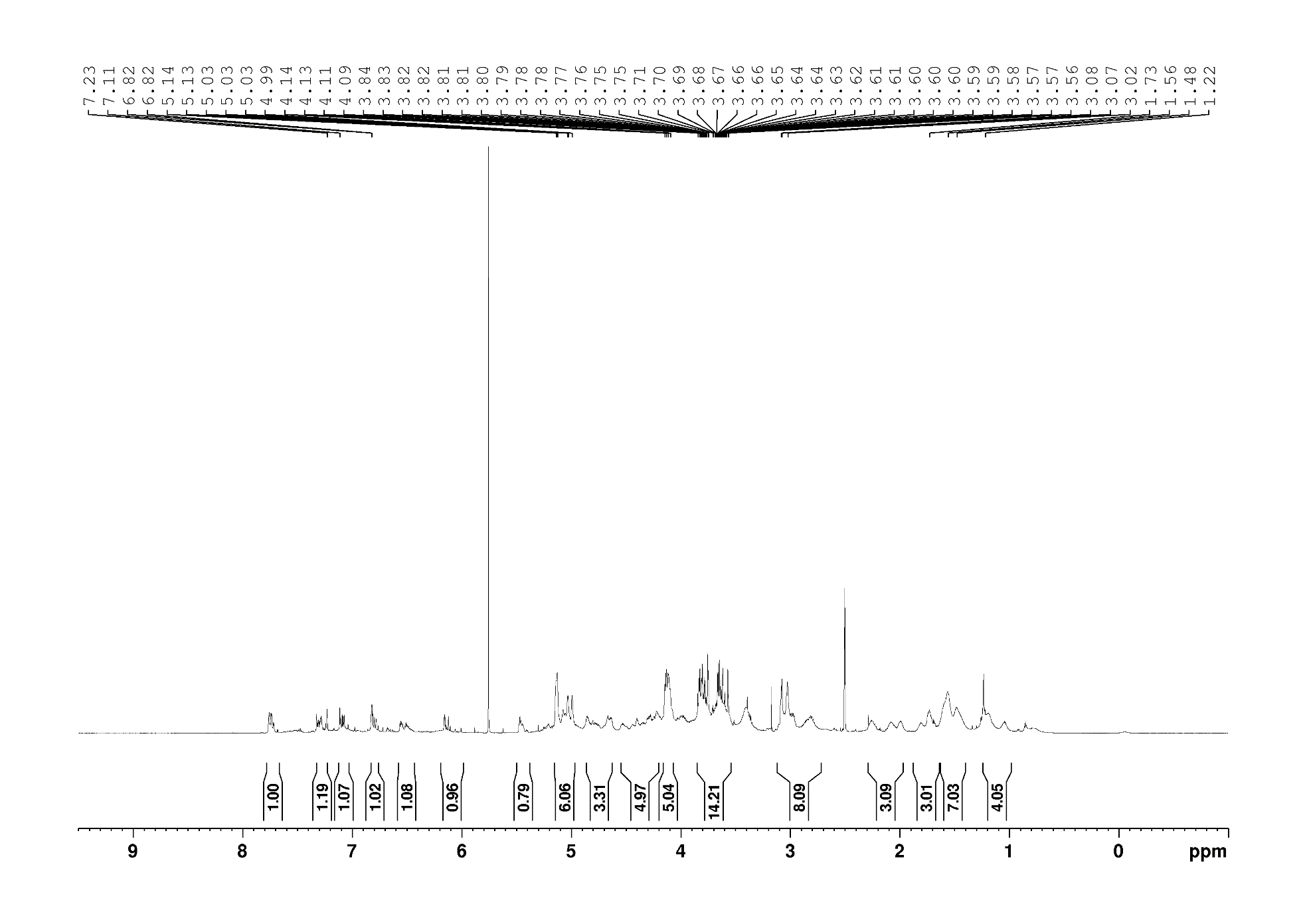


**(*S*)-7-methoxy-2-methylene-5-oxo-2,3,5,11a-tetrahydro-1H-benzo[e]pyrrolo[1,2-a] [1,4]diazepin-8-yl (((5aS,6R,6aR)-1-(2-(((*S*)-7-methoxy-2-methylene-5-oxo-2,3,5,11a-tetrahydro-1H-benzo[e]pyrrolo[1,2-a][1,4]diazepin-8-yl)oxy)ethyl)-1,4,5,5a,6,6a,7,8-octahydrocyclopropa[5,6]cycloocta[1,2-d][1,2,3]triazol-6-yl)methyl) carbonate (D4)**


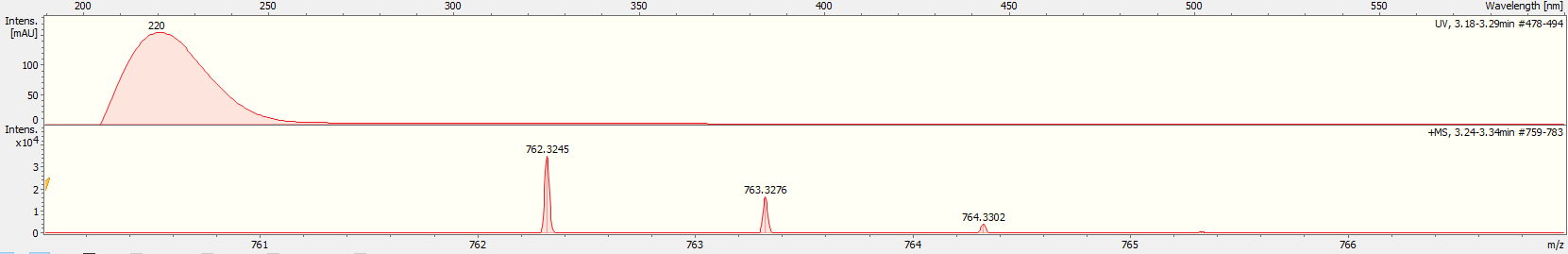


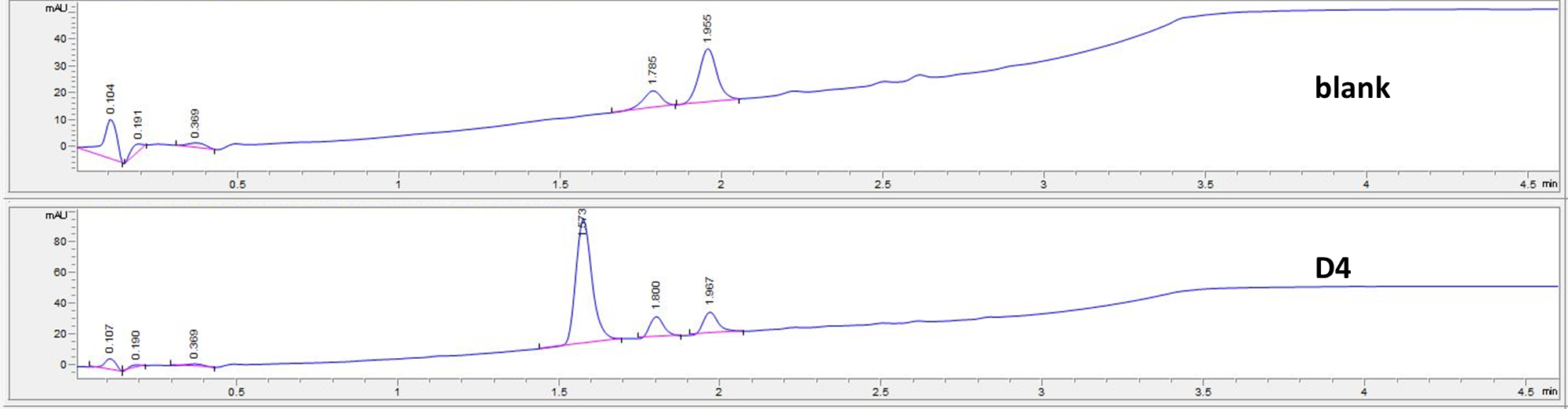


94% calculated purity by HPLC.

//nmrxiv.org/project/zHJDzpEvl4KhtWqYMyFd63edQc3poLvawWErPIcA


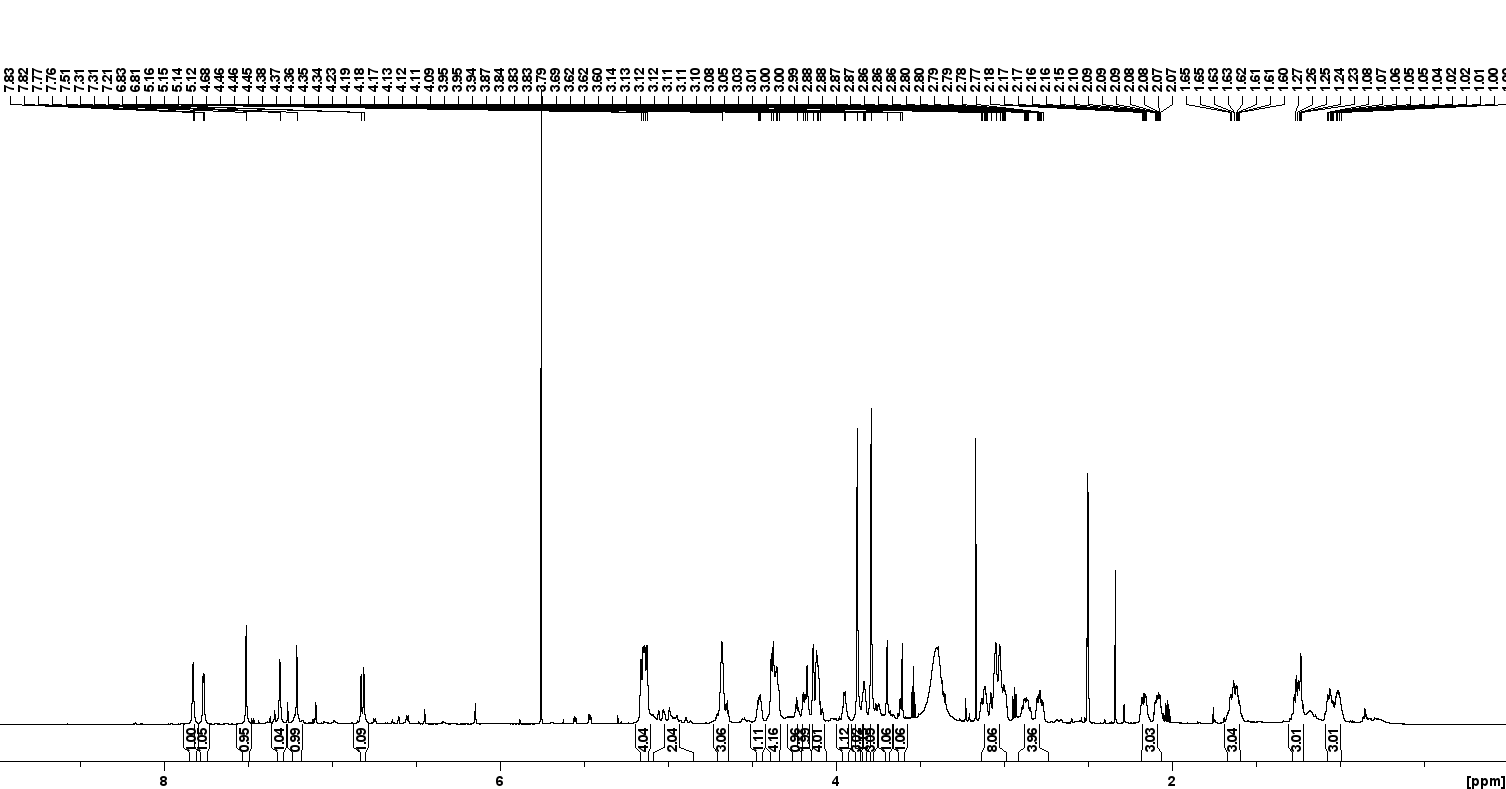


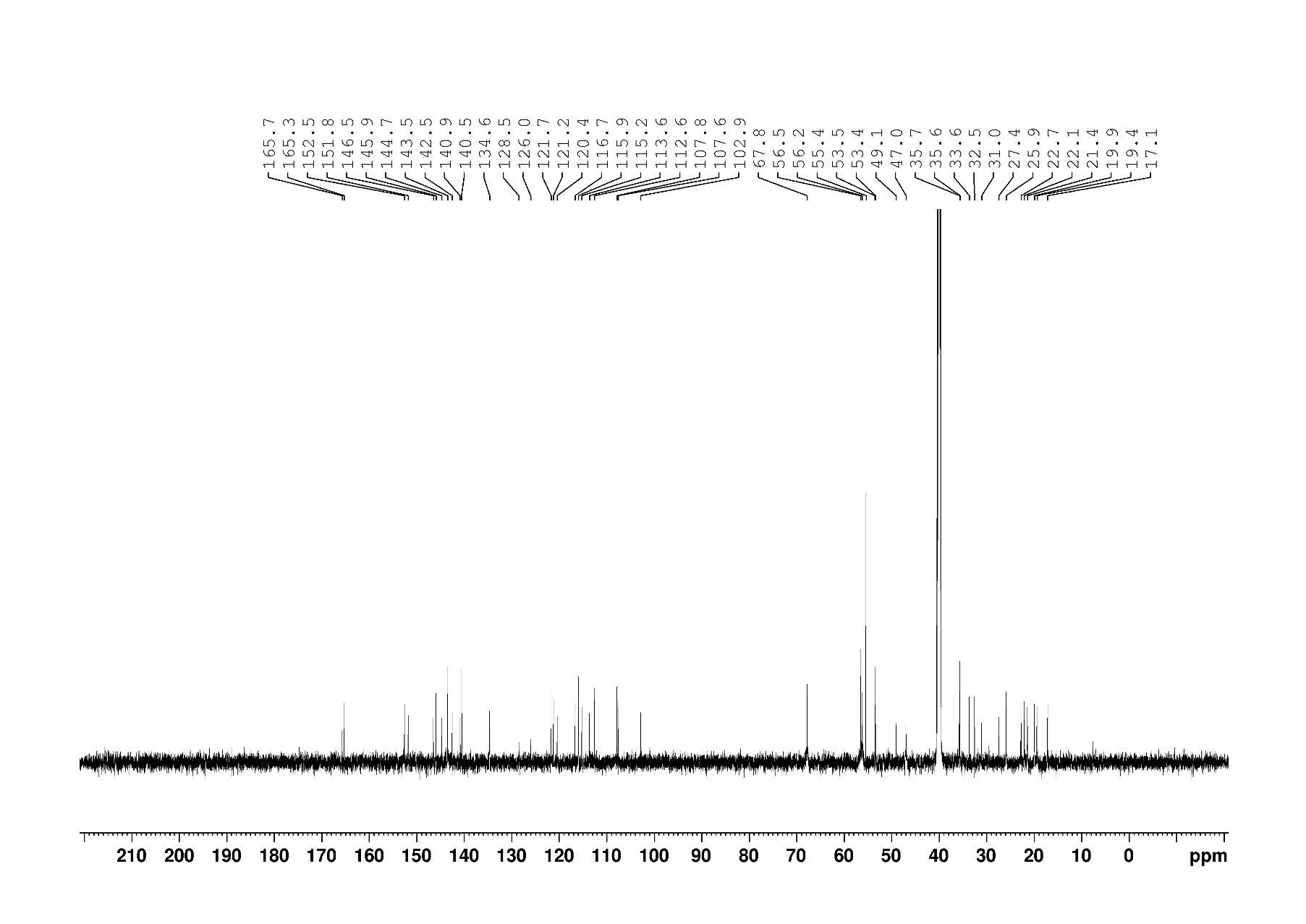

Supplement: Supplementary file 1 — Supporting Information [file CHEM-31-e01797-s001.docx]
